# *N*1-acetylspermidine is a determinant of hair follicle stem cell fate

**DOI:** 10.1242/jcs.252767

**Published:** 2021-05-11

**Authors:** Kira Allmeroth, Christine S. Kim, Andrea Annibal, Andromachi Pouikli, Janis Koester, Maxime J. Derisbourg, Carlos Andrés Chacón-Martínez, Christian Latza, Adam Antebi, Peter Tessarz, Sara A. Wickström, Martin S. Denzel

**Affiliations:** 1 Max Planck Institute for Biology of Ageing, Joseph-Stelzmann-Str. 9b, D-50931 Cologne, Germany; 2 CECAD - Cluster of Excellence, University of Cologne, Joseph-Stelzmann-Str. 26, D-50931 Cologne, Germany; 3 Helsinki Institute for Life Science, Biomedicum Helsinki, Haartmaninkatu 8, FI-00290 Helsinki, Finland; 4 Wihuri Research Institute, Biomedicum Helsinki, Haartmaninkatu 8, FI-00290 Helsinki, Finland; 5 Stem Cells and Metabolism Research Program, Faculty of Medicine, Biomedicum Helsinki, Haartmaninkatu 8, FI-00290 Helsinki, Finland; 6 Center for Molecular Medicine Cologne (CMMC), University of Cologne, Robert-Koch-Str. 21, D-50931 Cologne, Germany

**Keywords:** Hair follicle stem cells, Polyamines, Cell fate, mRNA translation

## Abstract

Stem cell differentiation is accompanied by increased mRNA translation. The rate of protein biosynthesis is influenced by the polyamines putrescine, spermidine and spermine, which are essential for cell growth and stem cell maintenance. However, the role of polyamines as endogenous effectors of stem cell fate and whether they act through translational control remains obscure. Here, we investigate the function of polyamines in stem cell fate decisions using hair follicle stem cell (HFSC) organoids. Compared to progenitor cells, HFSCs showed lower translation rates, correlating with reduced polyamine levels. Surprisingly, overall polyamine depletion decreased translation but did not affect cell fate. In contrast, specific depletion of natural polyamines mediated by spermidine/spermine *N*1-acetyltransferase (SSAT; also known as SAT1) activation did not reduce translation but enhanced stemness. These results suggest a translation-independent role of polyamines in cell fate regulation. Indeed, we identified *N*1-acetylspermidine as a determinant of cell fate that acted through increasing self-renewal, and observed elevated *N*1-acetylspermidine levels upon depilation-mediated HFSC proliferation and differentiation *in vivo*. Overall, this study delineates the diverse routes of polyamine metabolism-mediated regulation of stem cell fate decisions.

This article has an associated First Person interview with the first author of the paper.

## Abstract (precis)

**Summary:** Reduced protein synthesis is required for stem cell functions. Here, we delineate a complex interplay of polyamines and mRNA translation that determines hair follicle stem cell fate decisions.

## INTRODUCTION

mRNA translation is one of the most complex and energy-consuming cellular processes (Roux and Topisirovic, 2018). It comprises initiation, elongation, termination and ribosome recycling, and plays a key role in gene expression (Hershey et al., 2019). The rate of protein synthesis is tightly controlled by signaling pathways that sense various internal and external stimuli (Roux and Topisirovic, 2018). Deregulation of translation can manifest in a variety of diseases, including neurodegeneration and cancer (Tahmasebi et al., 2018). The regulation of mRNA translation has also been implicated in early cell fate transitions (Ingolia et al., 2011; Kristensen et al., 2013; Lu et al., 2009). Several studies in both embryonic and somatic stem cells demonstrate that global translation is suppressed in stem cells to retain them in an undifferentiated state, whereas translation is increased in progenitor cells upon differentiation (Baser et al., 2019; Sampath et al., 2008; Signer et al., 2014; Zismanov et al., 2016).

Hair follicle stem cells (HFSCs) represent an excellent paradigm to study adult somatic stem cell fate decisions, since they fuel cyclical rounds of hair follicle regeneration during the natural hair cycle (Blanpain and Fuchs, 2009; Fuchs et al., 2001). Blanco et al. (2016) showed that the activation of HFSCs during the transition from the resting phase, telogen, to proliferation in anagen during the hair cycle also coincides with increased translation. These data demonstrate a functional role for increased translation during differentiation in the epidermis. However, the mechanisms that regulate translation in stem cells in general and upon differentiation remain poorly understood.

One important determinant of translation rates is the availability of the natural polyamines putrescine, spermidine and spermine. Polyamines are polycations that are essential for cell growth. They bind to a variety of negatively charged cellular molecules, including nucleic acids (Dever and Ivanov, 2018). Polyamine homeostasis is critical to cell survival and is achieved by coordinated regulation at different levels of their synthesis, degradation, uptake and excretion (Wallace et al., 2003). Interestingly, up to 15% of polyamines are stably associated with ribosomes in *Escherichia coli*, highlighting their importance in protein synthesis (Cohen and Lichtenstein, 1960). Additionally, the polyamine spermidine is converted into the amino acid hypusine, which modifies the elongation factor eIF5A (Park et al., 1981). This modification is critical for translation elongation, especially for difficult substrates like polyproline (Gutierrez et al., 2013; Saini et al., 2009).

Given that increased polyamine levels positively regulate translation and that high translation rates promote stem cell differentiation, it is surprising that previous studies have implicated elevated polyamine levels in stem cell maintenance. For example, polyamines positively influence expression of MINDY1, a deubiquitinating enzyme, which promotes embryonic stem cell (ESC) self-renewal (James et al., 2018). Additionally, forced overexpression of one of the rate-limiting enzymes in polyamine biosynthesis, namely adenosylmethionine decarboxylase (AMD1) and ornithine decarboxylase (ODC), in ESCs results in delayed differentiation upon leukemia inhibitory factor (LIF) removal and improves cellular reprogramming (Zhang et al., 2012; Zhao et al., 2012). In line with these observations, differentiation of human bone marrow-derived mesenchymal stem cells coincides with decreased polyamine levels (Tsai et al., 2015). These data suggest that changes in polyamine levels can affect cell fate. However, the link between polyamines and translation in cell fate decisions remains unclear. Do low polyamine levels endogenously reduce translation rates in stem cells? Does reduction of polyamine levels, and translation, affect stem cell maintenance and function? Might distinct polyamine species differ in their effects on stem cells through translation or other mechanisms?

We addressed these questions in a HFSC organoid culture. Surprisingly, low translation rates achieved by changes in polyamine availability did not correlate with stemness in the organoids. Instead, accumulation of acetylated polyamines favored the stem cell state. Intriguingly, we identified *N*1-acetylspermidine (*N*1-AcSpd) as a determinant of HFSC fate, affecting cell proliferation. Forced HFSC proliferation and differentitation upon depliation in mice led to elevated *N*1-AcSpd levels, suggesting that acetylated polyamines play a functional role in cell fate decisions *in vivo*.

## RESULTS

### Low translation rates and enhanced protein quality control mark the HFSC state in 3D–3C organoids

**Fig. 1.**
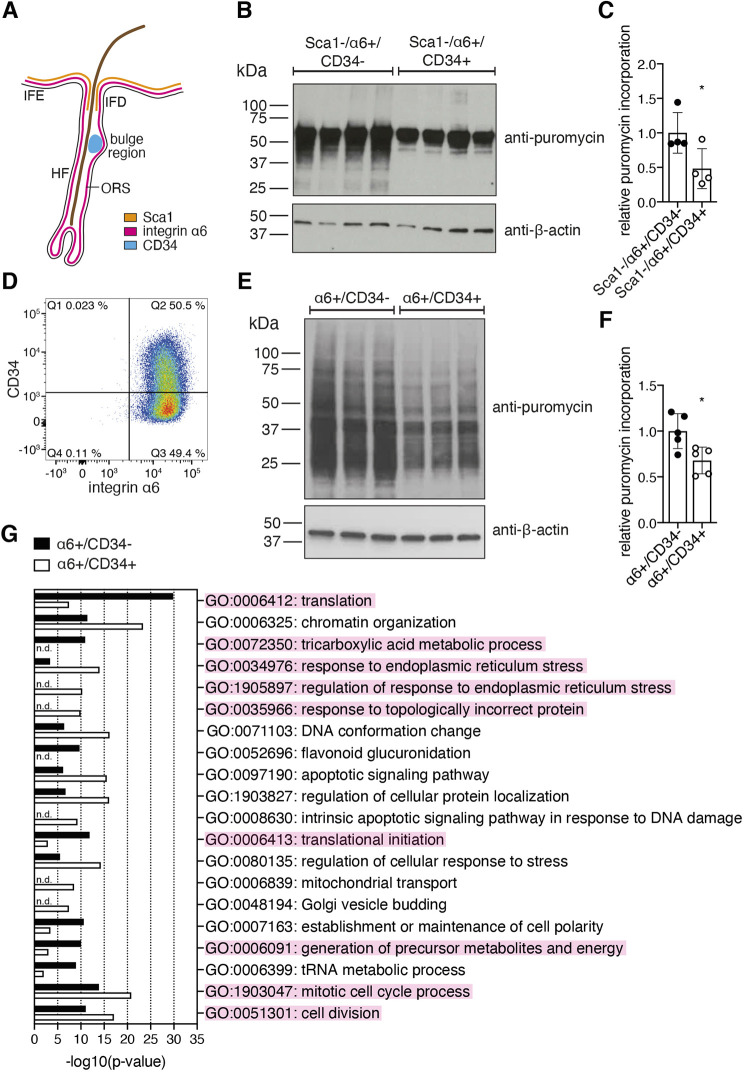
**Low translation rates and elevated protein quality control mark the HFSC state in the 3D–3C organoids.** (A) Schematic of marker expression in the hair follicle (HF). The interfollicular epidermis (IFE) and the infundibulum (IFD) region of the HF are marked by Sca1 expression. Integrin α6 is expressed in the basal layer of the IFE and the HF. CD34 expression is restricted to the bulge region. ORS, outer root sheath. (B) Western blot analysis after puromycin incorporation in Sca1−/α6+/CD34− progenitor cells compared to Sca1−/α6+/CD34+ HFSCs. (C) Quantification of the western blot results from experiment as in B. Results are mean±s.d. (*n*=4). (D) Representative dot plot of 14 independent experiments showing the distribution of the populations after 2 weeks of 3D–3C culture using integrin α6 and CD34 as markers. The percentage of cells in each quadrant is indicated. (E) Representative western blot analysis after puromycin incorporation in α6+/CD34− progenitor cells compared to α6+/CD34+ HFSCs. (F) Quantification of the western blot results from experiment as in E. Results are mean±s.d. (*n*=5). (G) GO term analysis of all translated mRNAs comparing α6+/CD34− progenitors and α6+/CD34+ HFSCs (biological process, metascape.org). The 20 GO terms with the biggest difference in enrichment between the translatomes of progenitors and HFSCs are shown. n.d., not detected. Each dot in C,F represents one biological replicate (*n*). *P<0.05 [two-tailed unpaired *t*-test (C,F)].

Various types of stem cells display lower translation rates than their differentiated counterparts (Baser et al., 2019; Sampath et al., 2008; Signer et al., 2014; Zismanov et al., 2016). This concept also applies to the epidermis – HFSCs have lower protein synthesis rates than committed cells *in vivo* (Blanco et al., 2016). To test whether isolated HFSCs maintain low translation, we sorted freshly prepared mouse epidermal cells using three different markers (Fig. 1A) – α6 integrin, which is expressed in all progenitors both in the interfollicular epidermis (IFE) and the hair follicle (HF) (Li et al., 1998; Sonnenberg et al., 1991); the hematopoietic stem cell marker CD34, which marks the bulge stem cells of the HF (Trempus et al., 2003); stem cell antigen-1 (Sca1; also known as LY6A), which is expressed in the infundibulum (IFD) region of the HF and in the basal layer of the epidermis (Jensen et al., 2008). By gating for Sca1-negative cells, IFE and IFD progenitors can be separated from HF progenitors (Fig. S1A). Thus, we compared HF bulge stem cells (Sca1−/α6+/CD34+) to HF outer root sheath cells (Sca1−/α6+/CD34−). We performed puromycin incorporation assays to quantify *de novo* protein synthesis (Schmidt et al., 2009) and found a significant reduction in Sca1−/α6+/CD34+ stem cells (Fig. 1B,C), confirming reduced translation rates in HFSCs *in vivo* that are maintained upon isolation.

To understand the functional consequences of this difference in translation, we made use of an *in vitro* organoid culture system (3D–3C culture) allowing for the long-term culture and manipulation of HFSCs and their direct progeny (Chacón-Martínez et al., 2017). In this culture, cells maintain self-renewal capacity and multipotency and respond to similar differentiation signals as HFSCs *in vivo*. Analysis of the 3D–3C organoid culture is based on integrin α6 and CD34 expression – while freshly isolated epidermal cells contain ∼5% α6+/CD34+ HFSCs (Fig. S1B), a balance between α6+/CD34+ HFSCs and α6+/CD34− progenitor cells is formed in a self-driven process in the organoid culture (Fig. 1D). Based on their transcriptome and marker expression analysis, these progenitor cells represent HF outer root sheath (ORS) cells and inner bulge cells (Chacón-Martínez et al., 2017; Kim et al., 2020), both of which represent HFSC progeny and act as niche cells for HFSCs *in vivo* (Hsu et al., 2014). To confirm that α6+/CD34+ HFSCs are less differentiated compared to α6+/CD34− ORS progenitors, we sorted cells after 2 weeks of 3D–3C culture and analyzed gene expression by 3′ RNA-sequencing (RNA-seq) and quantitative (q)RT-PCR (Fig. S1C). Comparing global gene expression in the two populations, the most enriched GO term was ‘skin development’ (Fig. S1D). Moreover, expression of HFSC identity markers, as well as markers for ORS lineage progression and the inner bulge (Joost et al., 2020), were elevated in HFSCs and progenitors, respectively (Fig. S1E). As expected, gene expression was similar between our RNA-seq data and a previously published dataset (Fig. S1E) (Chacón-Martínez et al., 2017). Furthermore, we analyzed the expression of key HFSC marker genes that were previously described to be upregulated in bona fide *in vivo* HFSCs (Morris et al., 2004; Tumbar et al., 2004) by qRT-PCR; the expression of most stem cell marker genes was higher in HFSCs compared to progenitor cells (Fig. S1F). In summary, 3D–3C cultured α6+/CD34− progenitors and α6+/CD34+ HFSCs are clearly distinguishable at the gene expression level and represent distinct cellular states. Next, we investigated puromycin incorporation in the two cell populations after sorting and found reduced translation in α6+/CD34+ HFSCs (Fig. 1E,F). Thus, the 3D–3C organoids maintain this key *in vivo* property of HFSCs, making them a suitable model to study the influence of translation on cell fate.

We next aimed to mechanistically understand how *de novo* protein synthesis affects cell fate. To this end, we analyzed the translatome in sorted α6+/CD34− progenitors and α6+/CD34+ HFSCs by ribosome footprinting (Ingolia et al., 2009). Using deep sequencing of ribosome protected fragments, we identified actively translated open reading frames and calculated the translational efficiency for each transcript by normalizing the Ribo-sequencing results to RNA-seq results obtained from the same sample (Fig. S1G). Successful digestion of the mRNA upon RNase treatment was confirmed by polysome profiling (Fig. S1H) and the meta-profile revealed periodicity of the P-sites in the coding sequence with almost no signal in the untranslated regions close to the start and the stop codon (Fig. S1I), suggesting successful computation of the P-site offset (Lauria et al., 2018). Analysis of HFSC identity and linage progression markers confirmed that many of them were not only transcribed, but also translated differently in HFSCs compared to progenitors (Fig. S1J).

To identify global differences in translation, we performed functional enrichment analysis of all translated genes in α6+/CD34− progenitors and compared this to all translated genes identified in α6+/CD34+ HFSCs independently of their translational efficiency. The GO term ‘translation’ was strongly enriched in α6+/CD34− progenitors (Fig. 1G), supporting the puromycin incorporation data (Fig. 1E,F). HFSCs further show an increased response to endoplasmic reticulum (ER) stress, which elevates the protein folding capacity. Functional enrichment points to elevated self-renewal of HFSCs in the 3D–3C organoids, as previously reported (Chacón-Martínez et al., 2017). Of note, translation of genes involved in the tricarboxylic acid cycle was upregulated in progenitors. Also, the GO term ‘generation of precursor metabolites and energy’, which comprises, but is not limited to, genes involved in oxidative phosphorylation (OXPHOS) and the electron transport chain, was enriched in progenitors, supporting previous observations of a metabolic switch during HFSC lineage progression (Flores et al., 2017; Kim et al., 2020). Overall, analysis of the translatome highlights that the stem cell state in the 3D–3C organoids is marked by low mRNA translation, a high level of glycolysis and elevated protein quality control, which are well described key properties of stem cells. GO term analysis of the genes with significantly different translational efficiency in α6+/CD34- progenitors compared to α6+/CD34+ HFSCs supported the findings from the global translatome analysis: while progenitors displayed higher levels of translation (Fig. S1K), the ER stress response was specifically elevated in HFSCs (Fig. S1L).

### Depletion of natural polyamines is a feature of stem cells but fails to drive stemness *in vitro*

**Fig. 2.**
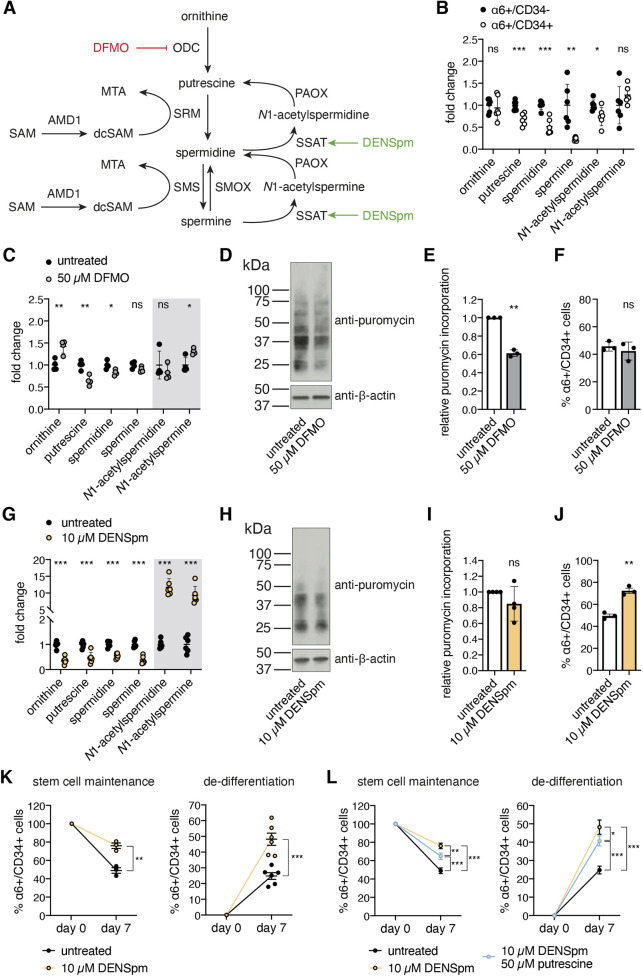
**Changes in polyamine availability enhance stemness independent from reduced mRNA translation.** (A) Schematic representation of the polyamine pathway. The effect of DENSpm is marked in green and DFMO is depicted in red. (B) Polyamine levels in sorted α6+/CD34− and α6+/CD34+ cells after 3D–3C culture. Results are mean±s.e.m. (*n*=6). (C) Polyamine levels in 3D–3C cultured cells with and without DFMO treatment. Results are mean±s.e.m. (*n*=4). (D) Representative western blot analysis after puromycin incorporation in 3D–3C cultured cells with or without DFMO treatment for the last 72 h of culture. (E) Quantification of western blot analysis from experiment as shown in D. Results are mean±s.d. (*n*=3). (F) Proportion (%) of α6+/CD34+ cells after 2 weeks of 3D–3C culture with or without DFMO treatment for 14 days. Results are mean±s.e.m. (*n*=3). (G) Polyamine levels in 3D–3C cultured cells with and without DENSpm treatment. Results are mean±s.e.m. (*n*=6). (H) Representative western blot analysis after puromycin incorporation in 3D–3C cultured cells with or without DENSpm treatment for the last 72 h of culture. (I) Quantification of western blot analysis from experiment as shown in H. Results are mean±s.d. (*n*=4). (J) Proportion (%) of α6+/CD34+ cells after 2 weeks of 3D–3C culture with or without DENSpm treatment for 14 days. Results are mean±s.e.m. (*n*=3). (K) Proportion (%) of α6+/CD34+ cells at day 0 and day 7 post-sorting starting from 100% α6+/CD34+ cells (left; *n*=3) or 100% α6+/CD34− cells (right; *n*=6) with or without DENSpm treatment. Results are mean±s.e.m. (L) Proportion (%) of α6+/CD34+ cells at day 0 and day 7 post-sorting starting from 100% α6+/CD34+ cells (left; *n*=3) or 100% α6+/CD34− cells (right; *n*=6) with or without DENSpm or additional putrescine treatment. Results are mean±s.e.m. For C,G, the gray box highlights the acetylated polyamines. For B–K, each dot represents one biological replicate (*n*). For F,J,K,L, analysis was performed in technical duplicates. For K,L, the treatment was performed for the last 5 days of culture. For L, dots represent the mean of the biological replicates (*n*). ***P<0.001; **P<0.01; *P<0.05; ns, not significant [two-tailed unpaired *t*-test (B,C,G,K); two-tailed paired *t*-test (E,F,I,J); two-way ANOVA Tukey's post-test (L)].

Since the availability of the natural polyamines is one important determinant of translation rates, we aimed to investigate the role of polyamines (Fig. 2A) in translational control of cell fate. To this end, we asked whether low polyamine levels are responsible for limited translation rates in stem cells. Indeed, when we sorted cells after 2 weeks of 3D–3C culture and measured the levels of polyamines by liquid chromatography–mass spectrometry (LC-MS), we found reduced levels of putrescine, spermidine and spermine in α6+/CD34+ HFSCs compared to α6+/CD34− progenitors (Fig. 2B).

To investigate the potential consequences of the low polyamine levels found in HFSCs (Fig. 2B) on cell fate decisions, we used 2-difluoromethylornithine (DFMO), which is an enzyme-activated, irreversible inhibitor of ODC (Metcalf et al., 1978), the first enzyme in the polyamine pathway (Fig. 2A). We measured polyamine levels in the 3D–3C organoids upon DFMO treatment and found that putrescine and spermidine levels were reduced, while ornithine accumulated (Fig. 2C). Of note, DFMO treatment recapitulated the low levels of natural polyamines seen in HFSCs compared to progenitors (Fig. 2B).

Depletion of the natural polyamines has been previously linked to decreased protein synthesis (Dever and Ivanov, 2018). Accordingly, puromycin incorporation was reduced by ∼40% in the organoid culture upon short-term DFMO treatment (72 h) (Fig. 2D,E). Although this reduction in mRNA translation mimicked the low translation rates seen in HFSCs (Fig. 1E,F), DFMO treatment was not sufficient to affect HFSC fate (Fig. 2F; Fig. S2A). To corroborate these results, we also delineated the effect of DFMO on the self-organizing plasticity of the organoid culture that results in a balance between α6+/CD34− and α6+/CD34+ cells (Chacón-Martínez et al., 2017). This balance is influenced by self-renewal and differentiation of HFSCs but also by proliferation and de-differentiation of progenitors. To investigate these processes separately, we sorted the cells after 2 weeks of 3D–3C culture to generate pure populations of either α6+/CD34− or α6+/CD34+ cells. These pure populations were cultured for 1 week with or without DFMO treatment for the last 5 days before analysis. Stem cell maintenance and de-differentiation were not affected by DFMO treatment (Fig. S2B). While DFMO slightly decreased the live cell number of α6+/CD34− progenitors, it did not affect α6+/CD34+ HFSC viability (Fig. S2C,D). Together, these data show that reduced mRNA translation by decreased polyamine availability does not enhance stemness in the 3D–3C organoids.

### DENSpm treatment regulates HFSC fate without reducing mRNA translation

To further probe polyamine function in stem cell fate decisions, we manipulated their levels using the spermine analog *N*1,*N*11-diethylnorspermine (DENSpm), which increases spermidine/spermine *N*1-acetyltransferase (SSAT; also known as SAT1) expression (Coleman et al., 1995; Fogel-Petrovic et al., 1996; Parry et al., 1995). During polyamine catabolism, SSAT acetylates spermidine and spermine using acetyl-CoA to form *N*1-acetylspermidine and *N*1-acetylspermine, respectively (Fig. 2A). Thus, DENSpm depletes natural polyamines, while the acetylated forms accumulate (Uimari et al., 2009). LC-MS analysis confirmed these changes upon DENSpm treatment in the organoids (Fig. 2G). The depletion of natural polyamines by DENSpm or SSAT overexpression (OE) has been shown to decrease mRNA translation in cultured cells (Landau et al., 2010; Mandal et al., 2013). However, in the organoid culture DENSpm did not affect puromycin incorporation (Fig. 2H,I). Yet, DENSpm significantly increased α6+/CD34+ stem cells after 2 weeks of 3D–3C culture (Fig. 2J). The balance between α6+/CD34− progenitors and α6+/CD34+ HFSCs is first established around day 9 in the 3D–3C organoids (Chacón-Martínez et al., 2017). To exclude that DENSpm increased the proportion of HFSCs through their specific selection from the mixed starting population (Fig. S1B), we initiated treatment on day 9. Remarkably, DENSpm elevated the number of α6+/CD34+ stem cells also when supplemented for the last 5 days of culture only (Fig. S2E).

To test for the functional relevance of the DENSpm-induced increase in stem cells, we analyzed the proliferative potential of the cells after 3D–3C culture in a colony formation assay. Importantly, α6+/CD34+ HFSCs display higher proliferative potential than α6+/CD34− progenitors when freshly isolated, but also after 3D–3C organoid culture (Kim et al., 2020; Trempus et al., 2003). Thus, this assay is a suitable read-out to confirm cell fate changes. For the colony formation assay, 3D–3C cultured cells were seeded at clonal density on a feeder layer and cultured for 2 to 3 weeks without treatment before emerging colonies were analyzed (Fig. S2F). Quantification of total colony number revealed a significant increase upon DENSpm treatment in the organoid culture, suggesting higher proliferative potential, and confirming an increase in functional stem cells (Fig. S2G,H). To investigate stem cell maintenance and de-differentiation separately, pure populations were sorted and cultured for 1 week with or without DENSpm for the last 5 days before analysis. Both stem cell maintenance and de-differentiation were elevated, resulting in increased α6+/CD34+ HFSCs on day 7 (Fig. 2K). To rule out that the changes in stem cell number and proliferative potential were secondary to an effect on cell proliferation, we measured EdU incorporation in both α6+/CD34+ and α6+/CD34− cells. EdU incorporation was not affected by DENSpm treatment in either of the cell populations (Fig. S2I). DENSpm treatment has been shown to induce cell detachment and apoptosis in glioblastoma cells (Tian et al., 2012). Therefore, we analyzed apoptosis by annexin V staining including detached cells from the cell culture supernatant in our analysis. While annexin V staining was generally higher in α6+/CD34− progenitors, DENSpm treatment did not affect apoptosis in any of the cell types (Fig. S2J). Thus, our data indicate that the promotion of the stem cell state by DENSpm occurred through a direct effect on stem cell fate.

DENSpm supplementation resulted in a depletion of the natural polyamines and an accumulation of the acetylated forms in the 3D–3C organoids (Fig. 2G). To elucidate which of these effects was responsible for the increase in stemness, we supplemented DENSpm-treated cells with putrescine to rescue the reduction of natural polyamines. When starting from pure populations the double treatment showed a partial rescue compared to DENSpm alone; stem cell maintenance and de-differentiation were decreased (Fig. 2L). These data indicated that the depletion of the natural polyamines partially contributed to the observed cell fate changes. However, stem cell maintenance and de-differentiation were still significantly affected upon the double treatment compared to untreated cells (Fig. 2L). There are three possible explanations for this result: first, the putrescine concentration used was too low to replenish the natural polyamines; second, putrescine itself affected cell fate; third, DENSpm treatment determined HFSC fate by accumulating acetylated polyamines.

Spermine analogs, like DENSpm, have been described to inhibit cell growth owing to the depletion of the natural polyamines (Libby et al., 1989; Porter et al., 1991). Accordingly, DENSpm treatment reduced the live-cell number of α6+/CD34− progenitors and α6+/CD34+ HFSCs (Fig. S2K,L). However, additional putrescine supplementation rescued this defect in cell growth (Fig. S2K,L), suggesting that the putrescine concentration used was sufficient to replenish the natural polyamines. Collectively, our data demonstrate that α6+/CD34+ stem cells have lower polyamine levels. However, the reduction of mRNA translation through decreased polyamine levels was not sufficient to enhance stemness. Instead, our data suggest a translation-independent role of polyamine availability in cell fate decisions; DENSpm supplementation, which did not affect translation, favored the HFSC state.

### *N*1-acetylspermidine is a determinant of HFSC fate

**Fig. 3.**
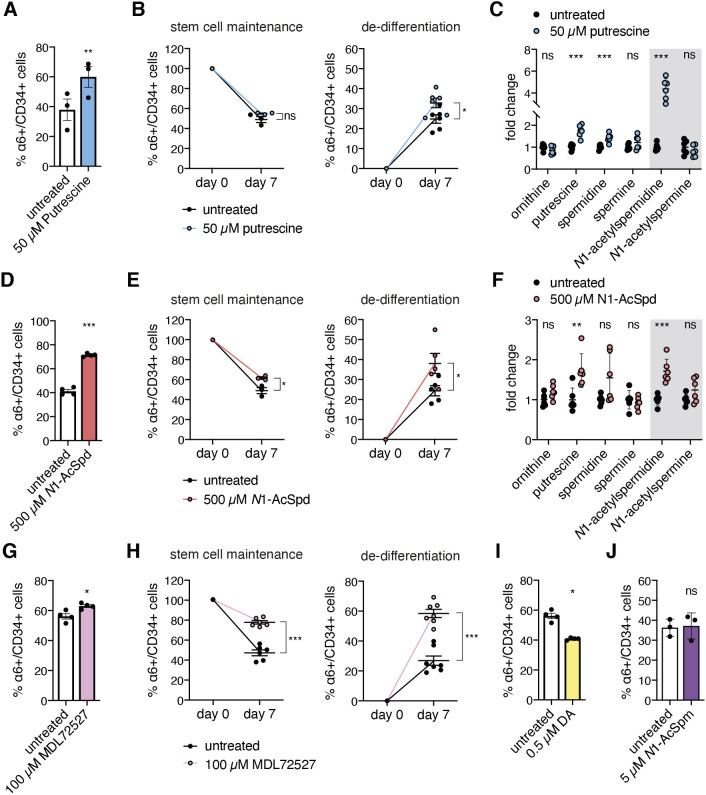
***N*1-acetylspermidine is a novel regulator of HFSC fate.** (A) Proportion (%) of α6+/CD34+ cells after 2 weeks of 3D–3C culture with or without putrescine treatment for 14 days. Results are mean±s.e.m. (*n*=3). (B) Proportion (%) of α6+/CD34+ cells at day 0 and day 7 post-sorting starting from 100% α6+/CD34+ cells (left; *n*=3) or 100% α6+/CD34− cells (right; *n*=6) with or without putrescine treatment. Results are mean±s.e.m. (C) Polyamine levels in 3D–3C cultured cells with and without putrescine treatment. Results are mean±s.e.m. (*n*=6). (D) Proportion (%) of α6+/CD34+ cells after 2 weeks of 3D–3C culture with or without *N*1-AcSpd treatment. Results are mean±s.e.m. (*n*=3). (E) Proportion (%) of α6+/CD34+ cells at day 0 and day 7 post-sorting starting from 100% α6+/CD34+ cells (left; *n*=3) or 100% α6+/CD34− cells (right; *n*=5) with or without *N*1-AcSpd treatment. Results are mean±s.e.m. (F) Polyamine levels in 3D–3C cultured cells with and without *N*1-AcSpd treatment. Results are mean±s.e.m. (*n*=6). (G) Proportion (%) of α6+/CD34+ cells after 2 weeks of 3D–3C culture with or without MDL72527 treatment for 14 days. Results are mean±s.e.m. (*n*=4). (H) Proportion (%) of α6+/CD34+ cells at day 0 and day 7 post-sorting starting from 100% α6+/CD34+ cells (left; *n*=6) or 100% α6+/CD34− cells (right; *n*=7) with or without MDL72527 treatment. Results are mean±s.e.m. (I) Proportion (%) of α6+/CD34+ cells after 2 weeks of 3D–3C culture with or without diminazene aceturate (DA) treatment for 14 days. mean±s.e.m. (*n*=4). (J) Ratio of α6+/CD34+ cells after two weeks of 3D–3C culture with or without *N*1-AcSpm treatment for 14 days. Results are mean±s.e.m. (*n*=3). For C,F, the gray box highlights the acetylated polyamines. For A–J, each dot represents one biological replicate (*n*). For A,B,D,E,G–J, analysis was performed in technical duplicates. For B,E,H, the treatment was performed for the last 5 days of culture. ***P<0.001; **P<0.01; *P<0.05; ns, not significant [paired two-tailed *t*-test (A,D,G,I,J); unpaired two-tailed *t*-test (B,C,E,F,H)].

We next tested whether putrescine supplementation itself affects cell fate in the organoid culture. Remarkably, putrescine treatment in the 3D–3C organoids resulted in an increase in the number of α6+/CD34+ stem cells, when supplemented for the whole duration of the culture (Fig. 3A). In contrast, putrescine treatment for the last 5 days of culture was not sufficient to enhance stemness (Fig. S3A). Increased stemness was confirmed by elevated colony-forming ability, showing enhanced proliferative potential (Fig. S3B,C). Surprisingly, putrescine supplementation specifically enhanced de-differentiation of α6+/CD34− progenitors to α6+/CD34+ HFSCs, while stem cell maintenance was not affected (Fig. 3B). Putrescine supplementation did not affect the live-cell number of progenitors or HFSCs (Fig. S3D,E). In addition, puromycin incorporation was unchanged upon short-term putrescine addition (72 h) (Fig. S3F,G). To further investigate the effect of putrescine supplementation, we measured intracellular polyamine levels in the 3D–3C organoids. Spermidine and *N*1-acetylspermidine (*N*1-AcSpd) levels were increased in response to putrescine treatment (Fig. 3C). In addition, an elevation of putrescine confirmed the effectiveness of our treatment. In sum, increased polyamine availability upon putrescine addition is sufficient to enhance de-differentiation of progenitors, while stem cell maintenance is not affected. Thus, the putrescine-mediated effect cannot account for the changes seen with the double treatment.

Since both DENSpm and putrescine supplementation led to elevated *N*1-AcSpd levels (Figs 2G and 3C), we speculated that *N*1-AcSpd accumulation might cause the observed cell fate changes. Intriguingly, *N*1-AcSpd treatment significantly increased the number of α6+/CD34+ stem cells in the 3D–3C organoids (Fig. 3D). Supplementation for the last 5 days of culture was sufficient to favor the stem cell state (Fig. S3H). Stemness was confirmed by enhanced proliferative potential in the colony formation assay (Fig. S3I,J). In contrast to what was seen with putrescine addition, *N*1-AcSpd treatment enhanced not only de-differentiation, but also stem cell maintenance (Fig. 3E). While the viability of α6+/CD34− progenitors was not affected by *N*1-AcSpd treatment, the live cell number was increased in α6+/CD34+ stem cells (Fig. S3K,L). This increase might contribute to the shift in the stem cell ratio; however, since *N*1-AcSpd supplementation also enhanced de-differentiation without affecting progenitor viability, it likely influenced cell fate decisions.

Next, we measured intracellular polyamine levels upon *N*1-AcSpd treatment and found that, apart from *N*1-AcSpd, putrescine levels were also increased (Fig. 3F). Thus, the effect of *N*1-AcSpd treatment on intracellular polyamines resembled that with putrescine supplementation (Fig. 3C). To specifically elevate *N*1-AcSpd and to separate the impact of *N*1-AcSpd from putrescine-mediated effects, we prevented the conversion of *N*1-AcSpd into putrescine by using the polyamine oxidase (PAOX) inhibitor MDL72527 (Bey et al., 1985). Intriguingly, MDL72527 treatment significantly increased the number of α6+/CD34+ stem cells in the 3D–3C organoids (Fig. 3G). Treatment for the last 5 days of the culture was sufficient to affect cell fate (Fig. S4A). When starting from pure populations, MDL72527 addition elevated stem cell maintenance and de-differentiation (Fig. 3H). These effects were accompanied by reduced live-cell numbers of α6+/CD34− progenitors and α6+/CD34+ stem cells (Fig. S4B,C). Since the viability of α6+/CD34− progenitors was reduced to a greater extent, the increase in the proportion of HFSCs might be partially caused by selective loss of progenitor cells. Nevertheless, de-differentiation was affected by MDL72527 addition, suggesting a change in cell fate decisions. Overall, the MDL72527-mediated effects resembled those seen upon DENSpm treatment. This finding further supports the hypothesis that the accumulation of *N*1-AcSpd determines cell fate decisions in DENSpm-treated cultures.

To specifically deplete *N*1-AcSpd, we used the SSAT inhibitor diminazene aceturate (DA) (Neidhart et al., 2014; Wu et al., 1996). Remarkably, DA treatment significantly reduced the number of α6+/CD34+ stem cells in the 3D–3C organoids (Fig. 3I). Treatment for the last 5 days of the culture was sufficient to recapitulate this effect (Fig. S4D). Both stem cell maintenance and de-differentiation were reduced by DA addition (Fig. S4E). Interestingly, DA treatment decreased the live-cell number of α6+/CD34− progenitors, but did not affect the viability of α6+/CD34+ stem cells (Fig. S4F,G). Thus, the reduction of the proportion of HFSCs would be even more pronounced if the viability of the progenitor cells was not affected. Puromycin incorporation was unchanged upon MDL72527 and DA addition (Fig. S4H,I). Taken together, the data obtained upon inhibition of SSAT and PAOX confirm that the accumulation of *N*1-AcSpd is not only required but also sufficient to enhance stemness in the HFSC organoids.

Since DENSpm and MDL72527 affect both *N*1-AcSpd and *N*1-AcSpm levels, we aimed to investigate the effect of *N*1-AcSpm treatment on cell fate. In stark contrast to what was seen with *N*1-AcSpd treatment, supplementation with *N*1-AcSpm did not influence the proportion of HFSCs (Fig. 3J; Fig. S4J). Stem cell maintenance and de-differentiation were not affected by *N*1-AcSpm treatment (Fig. S4K). The live-cell number tended to be decreased in α6+/CD34− progenitors and increased in α6+/CD34+ stem cells; however, this effect was not significant (Fig. S4L,M). Surprisingly, short-term *N*1-AcSpm (72 h) treatment reduced puromycin incorporation in the organoid culture (Fig. S4N,O). Overall, the data demonstrate a specific effect of *N*1-AcSpd treatment on cell fate.

### *N*1-acetylspermidine treatment affects HFSC fate by increasing proliferation

**Fig. 4.**
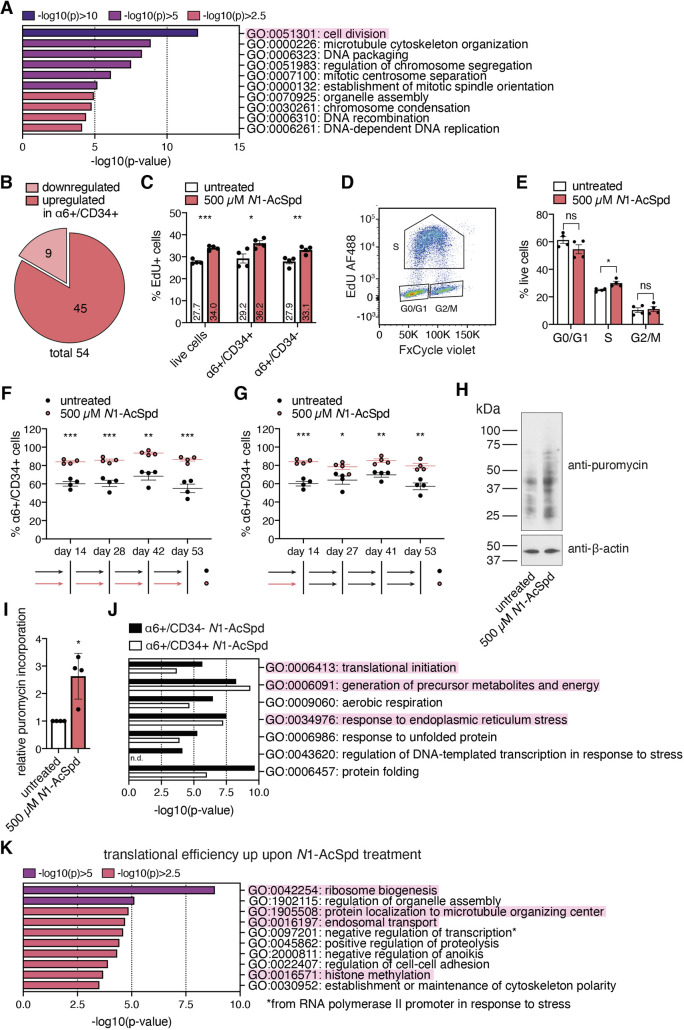
***N*1-acetylspermidine treatment affects cell fate by promoting cell cycle progression.** (A) GO term analysis of differentially regulated genes between cell types (*P*<0.05, log2FC >±0.5) only upon *N*1-AcSpd treatment (depicted in red in Fig. S5A, biological process; https://metascape.org/). (B) Of the 54 genes that belong to the GO term ‘cell division’ and were found to be differentially expressed upon *N*1-AcSpd treatment (highlighted in Fig. S5C), 9 genes were downregulated (light red) and 45 genes were upregulated (dark red). (C) Proportion (%) of EdU+ cells with or without *N*1-AcSpd treatment for the last 72 h of culture. EdU was incorporated for 2 h. mean±s.e.m. (*n*=4). (D) Dot plot of FxCycle Violet intensity and EdU AF488 intensity, separating the phases of the cell cycle (G0/G1, S, G2/M) as indicated. (E) Proportion (%) of live cells with or without *N*1-AcSpd treatment for the last 72 h of culture according to their cell cycle phase distribution. Results are mean±s.e.m. (*n*=4). (F) Proportion (%) of α6+/CD34+ cells in long-term 3D–3C culture with or without *N*1-AcSpd treatment. mean±s.e.m. (*n*=4). (G) Proportion (%) of α6+/CD34+ cells in long-term 3D–3C culture with or without *N*1-AcSpd treatment. The treatment was stopped after the first passage on day 14. Results are mean±s.e.m. (*n*=4). (H) Representative western blot analysis after puromycin incorporation in 3D–3C cultured cells with or without *N*1-AcSpd treatment for the last 72 h of culture. (I) Quantification of western blot analysis results from experiment as in H. Results are mean±s.d. (*n*=4). (J) GO term analysis of all translated genes comparing α6+/CD34− progenitor cells and α6+/CD34+ HFSCs upon *N*1-AcSpd treatment (biological process; https://metascape.org/). GO terms that were found to be differently enriched in the translatome of untreated progenitors versus HFSCs are shown. n.d., not detected. (K) GO term analysis of differentially translated genes between cell types (*P*<0.05) with higher translational efficiency in *N*1-AcSpd-treated cells compared to untreated cells (biological process; https://metascape.org/). For C,E–G,I, each dot represents one biological replicate (*n*); analysis was performed in technical duplicates. ***P<0.001; **P<0.01; *P<0.05; ns, not significant [unpaired two-tailed *t*-test (C,E–G); paired two-tailed *t*-test (I)].

To investigate the molecular mechanism by which *N*1-AcSpd supplementation promotes stemness, we performed 3′ RNA-seq of 3D–3C cultured cells upon short-term *N*1-AcSpd treatment (72 h). HFSCs and progenitor cells were purified and analyzed separately (Fig. S1C). We compared α6+/CD34− cells with α6+/CD34+ cells to identify differentially expressed genes for untreated and *N*1-AcSpd-treated cells (Fig. S5A). The two groups of differentially expressed genes were subjected to further analysis. Around 2200 genes with differential expression between cell types were not affected by *N*1-AcSpd treatment (Fig. S5B); 851 genes were differentially expressed between HFSCs and progenitors only in the presence of *N*1-AcSpd, while 540 genes were differentially expressed between cell types exclusively in untreated cells (Fig. S5B). Gene ontology (GO) term analysis was performed with the three different groups using Metascape (Zhou et al., 2019). Strikingly, analysis of the genes differentially expressed between HFSCs and progenitors only during *N*1-AcSpd treatment revealed that most of the ten highest ranked GO terms were linked to cell cycle progression (Fig. 4A). The most significant GO term was cell division, of which 54 genes were present among the 851 differentially expressed genes in *N*1-AcSpd treatment (Fig. S5C). Interestingly, expression of the majority of these 54 genes was higher in α6+/CD34+ stem cells (Fig. 4B). Importantly, *N*1-AcSpd treatment affected the amplitude of the fold change; the expression of these genes was higher also in the untreated α6+/CD34+ stem cells (Table S1). Consistently, EdU incorporation was higher in untreated α6+/CD34+ HFSCs compared to progenitor cells (Fig. S5D). Together, these data suggest that *N*1-AcSpd selectively promoted self-renewal of HFSCs. GO term analysis of the ∼2200 genes that differed between HFSCs and progenitors without being affected by *N*1-AcSpd revealed ‘skin development’ as the most significant GO term (Fig. S5E). This suggests that *N*1-AcSpd treatment did not affect general cell identity in the 3D–3C organoids. Consistently, a comparison of the gene expression changes between cell types in a heat map showed a clear separation of α6+/CD34+ HFSCs and α6+/CD34− progenitors (Fig. S5F). Although *N*1-AcSpd treated cells clustered separately of control cells, their gene expression was very similar to the respective cell type in the control (Fig. S5F).

Since the analysis of global gene expression changes clearly indicated that *N*1-AcSpd treatment would affect proliferation, we measured EdU incorporation. Importantly, short-term *N*1-AcSpd supplementation (72 h) increased the number of EdU+ cells (Fig. 4C). The effect on α6+/CD34+ HFSCs was higher compared to α6+/CD34− progenitor cells (Fig. 4C). Still, consistent with increased de-differentiation and elevated stem cell maintenance, proliferation of both HFSCs and progenitors was affected by the treatment. Furthermore, we analyzed the cell cycle phase distribution of the cells using FxCycle Violet staining. Plotting of FxCycle Violet intensity on the *x*-axis and EdU Alexa Fluor 488 (AF488) intensity on the *y*-axis enabled clear separation of the different cell cycle phases (Fig. 4D). Consistent with increased EdU incorporation, we observed faster cell cycle progression with fewer cells in G0/G1 phase and more cells in S phase upon short-term *N*1-AcSpd treatment (72 h) (Fig. 4E). The number of cells in G2/M phase was not changed. Taken together, these data suggest that the acetylated polyamine *N*1-AcSpd fulfills a functional role in cell cycle progression and that it affects cell fate by increasing proliferation of both cell populations.

To test whether elevated proliferation seen upon *N*1-AcSpd treatment would result in premature stem cell exhaustion, we set up a long-term organoid culture. The cells were passaged every 11–14 days and the ratio of HFSCs to progenitors was repeatedly analyzed. The proportion of α6+/CD34+ HFSCs was steady at ∼60% in untreated and at ∼80% in *N*1-AcSpd-treated organoids (Fig. 4F), suggesting that the stem cells were not exhausted even under continuous treatment. Intriguingly, cell fate was also maintained in the long-term culture upon withdrawal of *N*1-AcSpd treatment after the first passage (Fig. 4G).

To test the dependency of the *N*1-AcSpd-mediated effect on mRNA translation, we next performed puromycin incorporation and ribosome footprinting. In line with the enhanced cell cycle progression, we found increased mRNA translation upon short-term *N*1-AcSpd treatment (72 h) (Fig. 4H,I). Ribosome footprinting confirmed that many of the HFSC identity markers were not only transcribed, but also translated differently in HFSCs compared to progenitors upon *N*1-AcSpd treatment (Fig. S5G). To investigate global changes, we performed functional enrichment analysis of all translated genes in α6+/CD34− progenitors compared to α6+/CD34+ HFSCs. As in untreated cells, proliferation was elevated in HFSCs upon *N*1-AcSpd treatment (Fig. S5H). This finding is in line with our EdU incorporation data, which also revealed higher proliferation of α6+/CD34+ HFSCs compared to progenitors (Fig. 4C). In contrast to untreated cells, the difference in enrichment for GO terms comprising translation, ER stress, and aerobic respiration was rather low between the total translatomes of the two cell types (Fig. 4J). These data suggest that *N*1-AcSpd treatment might bring the translatome of progenitor cells closer to that of HFSCs to influence cell fate changes in the organoid culture.

Next, we focused on the transcripts with a significantly different translational efficiency in α6+/CD34− progenitors compared to α6+/CD34+ HFSCs. Comparing these transcripts from untreated and *N*1-AcSpd-treated cells, the overlap at the transcript level was low (purple lines in Fig. S5I); however, many transcripts clustered at the GO term level (blue lines in Fig. S5I). Accordingly, some GO terms were enriched in both transcript lists (Fig. S5J). The GO term ‘ribonucleoprotein complex biogenesis’ was considerably more enriched upon *N*1-AcSpd treatment (Fig. S5J), suggesting an elevated requirement for new ribosomes due to enhanced mRNA translation. Moreover, the most enriched GO term, which was exclusively identified upon *N*1-AcSpd treatment, was ‘ribosome biogenesis’ (Fig. 4K). Together, these data suggest elevated mRNA translation, thereby confirming the increased puromycin incorporation seen upon *N*1-AcSpd treatment. Furthermore, *N*1-AcSpd treatment resulted in elevated translation of transcripts belonging to the GO term ‘protein localization to microtubule organizing center’ (Fig. 4K), indicating that the enhanced cell cycle progression seen in *N*1-AcSpd-treated cells (Fig. 4E) was not only transcriptionally but also translationally regulated. The addition of *N*1-AcSpd to the cell culture medium also induced translation of genes belonging to the GO term ‘endosomal transport’ (Fig. 4K). Although the exact mechanism of cellular polyamine uptake is largely unknown, the involvement of the endocytosis pathway has been suggested (Soulet et al., 2002). Thus, increased endosomal transport might be directly caused by elevated extracellular *N*1-AcSpd levels. Overall, the ribosome footprinting analysis confirmed elevated translation and cell cycle progression upon *N*1-AcSpd treatment. Furthermore, *N*1-AcSpd addition partially aligned the translatome of progenitors to the translatome of HFSCs, thereby likely facilitating cell fate changes.

### HFSC activation by depilation suggests a functional role of the acetylated polyamines in hair growth *in vivo*

Having established that *N*1-AcSpd treatment influences cell fate decisions in the organoid culture, we aimed to investigate whether *N*1-AcSpd also associates with cell fate decisions *in vivo*. To this end, we activated HFSCs *in vivo* by depilation, which forces hair follicle entry into anagen in the depilated area. As aged HFSCs have been shown to be more quiescent than young HFSCs and thus resistant to activation (Keyes et al., 2013), we further compared young (6 months) and aged (24 months) mice in this assay.

**Fig. 5.**
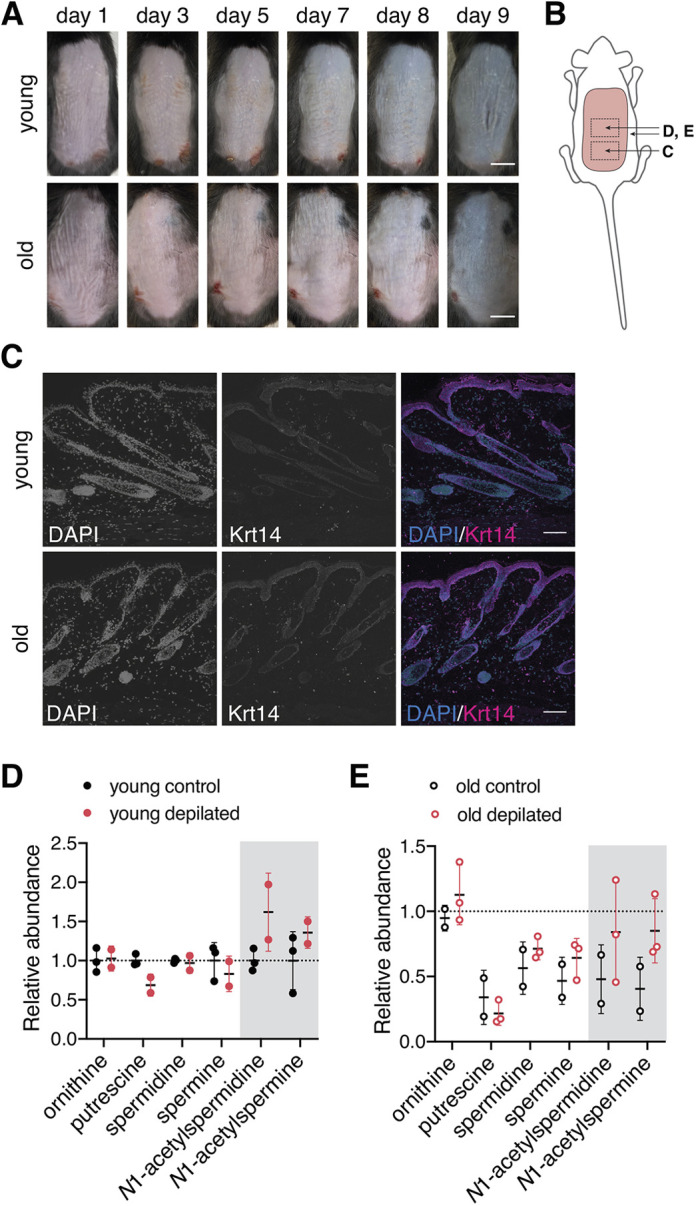
**Activation of hair growth increases the levels of the acetylated polyamines *in vivo*.** (A) Representative images of the depilated dorsal skin of young (6 months, top) and old (24 months, bottom) mice (*n*=4 for both groups). (B) Schematic representation of the depilated area (pink) and the areas used for sample collection (squares). (C) Representative immunofluorescence staining for keratin 14 (Krt14, magenta) in young (top) and old (bottom) mice. Nuclei were stained with DAPI (blue) (*n*=4 for both groups). (D) Polyamine levels in epidermis obtained from young mice comparing depilated epidermis (anagen; *n*=2) to control epidermis (telogen; *n*=3). Results are mean±s.d. (E) Polyamine levels in epidermis obtained from old mice comparing depilated epidermis (anagen; *n*=3) and control epidermis (telogen; *n*=2). Values are normalized to control epidermis from young mice (dotted line). Results are mean±s.d. for D,E, each dot represents one biological replicate (*n*). The gray box highlights the acetylated polyamines. Scale bars: 1 cm (A); 100 µm (C).

Because of the precise coupling of HF cycling and melanogenesis, the skin color changes from pink to black between 5 to 8 days after depilation, indicating the transition from telogen to anagen (Slominski and Paus, 1993). Consistently, in young mice, we observed a change of skin color starting on day 7 (Fig. 5A). However, the graying of the skin was delayed in the old mice, starting in the anterior region (neck) on day 8 (Fig. 5A).

The mice were killed on day 9 after depilation and tissue biopsies were collected for further analysis (Fig. 5B). It has been previously described that 9 days after depilation, the induced anagen hair follicles reach their maximal length and are morphologically indistinguishable from spontaneously developing anagen follicles (Müller-Röver et al., 2001). We confirmed that the hair follicles of young mice were in full anagen by immunofluorescence staining for keratin 14 (Fig. 5C). In contrast, the hair follicles of old mice were still in early anagen, as shown by reduced length of the hair shaft (Fig. 5C). These results confirmed the delay in hair growth determined by the changes in skin color (Fig. 5A). Finally, we measured the polyamine levels in control epidermis (telogen) and depilated epidermis (anagen) in young and in old mice. We found that depilation specifically increased the levels of the *N*1-AcSpd and *N*1-AcSpm in young mice (Fig. 5D). Interestingly, polyamine levels were generally lower in old mice and, although the levels of the acetylated polyamines also increased upon depilation in old mice, their levels remained lower than in young control epidermis (Fig. 5E).

Taken together, these results suggest that hair growth correlated with an accumulation of the acetylated polyamines. Together, with our findings in the organoid culture, these data suggest that increased *N*1-AcSpd levels are required for the full activation and differentiation of HFSCs *in vivo*.

## DISCUSSION

In this study, we delineate the polyamine-mediated regulation of HFSC maintenance and function. First, we show that low mRNA translation rates mark the HFSC state *in vivo* and in the organoid culture, and further identify key signatures of stemness in the translatome of HFSCs. Despite low polyamine levels in α6+/CD34+ stem cells, which might endogenously reduce protein synthesis, lowering translation rates by pharmacologically reducing polyamine levels does not influence stemness in the organoid culture. In contrast, accumulation of the acetylated polyamines favors the stem cell state. We identify *N*1-AcSpd as regulator of HFSC fate, affecting proliferation in the organoid culture. Going beyond *in vitro* analyses, we observe increased acetylated polyamines upon HFSC activation and differentiation *in vivo*.

Previously, Blanco et al. (2016) showed that translation is elevated during the hair cycle growth phase when cells become activated and differentiate. In contrast, quiescent HFSCs in the resting phase show low translation. This agrees with other studies showing that translation is upregulated during differentiation (Baser et al., 2019; Sampath et al., 2008; Signer et al., 2014; Zismanov et al., 2016). Importantly, we reproduce these findings upon HFSC isolation and in the 3D–3C organoid culture. Since the organoids maintain this key *in vivo* property of HFSCs, they represent a suitable model to study the influence of translation on cell fate. Of note, a forced inhibition of translation by loss of NOP2/Sun RNA methyltransferase family member 2 (NSUN2) in the mouse blocks the differentiation of HFSCs to progenitor cells (Blanco et al., 2011), clearly showing that upregulation of translation is necessary for differentiation in the hair follicle. However, reduction of translation by lowering polyamines was not sufficient to change cell fate in the organoids; neither stem cell maintenance nor progenitor cell de-differentiation were affected upon DMFO treatment. Instead, DENSpm treatment and *N*1-AcSpd supplementation, which do not reduce translation, favored the stem cell state.

To mechanistically understand how *de novo* protein synthesis affects cell fate, we analyzed the translatome in sorted progenitors and HFSCs by ribosome footprinting. Overall, analysis of the translatome revealed that HFSCs in the organoids have low mRNA translation rates, a high level of glycolysis and elevated protein quality control. Of note, these signatures are well-described key properties of stem cells in general. Several studies have shown that different types of stem cells rely on glycolysis for energy production, while OXPHOS is activated upon differentiation (Chen et al., 2008; Flores et al., 2017; Kondoh et al., 2007; Simsek et al., 2010). Furthermore, the suppression of the metabolic switch from glycolysis to OXPHOS and glutamine metabolism was reported to be required for ORS cells to return to the HFSC state (Kim et al., 2020), demonstrating a functional role of the metabolic profile in cell fate decisions. Interestingly, translation of a subset of mitochondrial genes involved in OXPHOS has been described to be particularly dependent on the hypusination of eIF5A (Puleston et al., 2019). Thus, polyamine availability might also affect cell fate through changes of mitochondrial function, which should be investigated in the future.

ESCs exhibit elevated levels of the proteasome subunit PSMD11, enhanced assembly of the proteasome, and, correspondingly, increased proteasome activity compared to more differentiated counterparts (Vilchez et al., 2012). Accordingly, the proteasome subunit PSMD14 is required for ESC self-renewal and cellular reprogramming (Buckley et al., 2012). Hematopoietic stem cells (HSCs) have fewer misfolded proteins compared to more differentiated counterparts (Hidalgo San Jose et al., 2020). However, an increase in protein synthesis causes the accumulation of misfolded proteins and the loss of HSCs, demonstrating that HSCs are dependent on low translation rates to maintain protein homeostasis and self-renewing capacity. Importantly, 3D–3C cultured cells showed increased puromycin incorporation compared to freshly isolated cells (Fig. 1B,C,E,F). This increase in mRNA translation might be sufficient to overwhelm the protein quality control machinery, resulting in misfolded proteins and subsequent ER stress. Since the maintenance of a healthy proteome is more fundamental in stem cells, protein quality control might be specifically increased in HFSCs. However, whether this signature is a precautionary measure to maintain a heathy proteome in HFSCs or an actual stress response cannot be concluded from our data and needs to be elucidated in future experiments.

Previously, acetylated polyamines were mostly described as the major group of polyamines exported from the cell (Seiler and Dezeure, 1990). Extending this notion, we find that the intracellular accumulation of *N*1-AcSpd has an effect on stemness. Although *N*1-AcSpd treatment and putrescine addition change intracellular polyamine levels comparably, we demonstrate that the effect on cell fate is mediated by *N*1-AcSpd. To this end, we prevented the conversion of putrescine into *N*1-AcSpd and vice versa. Importantly, the data obtained upon inhibition of SSAT and PAOX confirm that the accumulation of the acetylated polyamines is not only required but also sufficient to enhance stemness in the HFSC organoids. Since supplementation with *N*1-AcSpm did not influence the proportion of HFSCs, elevation of acetylated polyamines in general was not sufficient; instead, *N*1-AcSpd treatment has a specific effect on cell fate. Going beyond *in vitro* data, we identify a potential functional role of the acetylated polyamines in cell fate decisions *in vivo*; depilation-induced HFSC activation resulted in a specific accumulation of the acetylated polyamines in the epidermis. Our *in vitro* results suggest that the increase in *N*1-AcSpd levels is relevant. To further investigate the role of *N*1-AcSpd in cell fate decisions *in vivo*, topical application of *N*1-AcSpd to depilated skin would be conceivable in the future.

3′ RNA-seq of purified cell populations from the organoids revealed that *N*1-AcSpd treatment affected expression of cell cycle-associated genes. Interestingly, these same genes were found expressed at higher levels in α6+/CD34+ stem cells, which also display higher EdU incorporation compared to progenitor cells in the organoid cultures. Importantly, increased proliferation and stemness have been linked before. A study in ESCs suggests that elevated cyclin-dependent kinase (CDK) activity, and thus increased cell cycle progression, contributes to stem cell maintenance (Liu et al., 2017). Consistently, reprogramming efficiency seems to be linked to successful acceleration of the cell cycle (Guo et al., 2014; Ruiz et al., 2011). Of note, forced overexpression of AMD1 or ODC in mouse fibroblasts, resulting in the accumulation of polyamines, improves reprogramming efficiency (Zhang et al., 2012; Zhao et al., 2012). Our results suggest that this effect is caused by increased proliferation. Collectively, these data indicate that both stem cell maintenance and de-differentiation might be improved by elevated cell cycle progression upon *N*1-AcSpd treatment in the 3D–3C organoid culture.

Polyamines have been implicated in cell cycle progression before. Under normal growth conditions, polyamine levels are dynamically regulated during the cell cycle (Sunkara et al., 1981), which is due to cyclical changes in activity of ODC and AMD1 (Fredlund et al., 1995). Consistent with this, several studies have shown that polyamine depletion results in cell cycle arrest (Odenlund et al., 2009; Ray et al., 1999; Yamashita et al., 2013). Polyamines have been described to interact with DNA through electrostatic and hydrogen bonding forces, resulting in its stabilization (Flink and Pettijohn, 1975; Pallan and Ganesh, 1996; Tsukamoto et al., 2005). Moreover, polyamines change the conformation of the nucleosome core, thereby facilitating the replication of DNA (Morgan et al., 1987). Thus, polyamine availability might be required for optimal rates of DNA replication during S phase (Alm and Oredsson, 2009). Consistent with this, analysis of global translation upon *N*1-AcSpd treatment revealed the GO term ‘DNA replication’ (Fig. S5H). Additionally, the GO term ‘protein–DNA complex subunit organization’ was enriched in the same analysis and ‘histone methylation’ was identified when transcripts with higher translational efficiency upon *N*1-AcSpd treatment were analyzed. Together, these data suggest a role of *N*1-AcSpd in DNA replication. Moreover, potential epigenetic changes might explain the long-term effect of *N*1-AcSpd supplementation that persists after withdrawal of the treatment. Overall, analysis of the ribosome footprinting data upon *N*1-AcSpd treatment confirmed elevated translation and cell cycle progression. Furthermore, *N*1-AcSpd addition partially aligned the translatome of progenitors to the translatome of HFSCs, thereby likely facilitating cell fate changes.

In sum, this study dissects independent routes of polyamine-controlled regulation of cell fate – while depletion of natural polyamines endogenously reduced translation, addition of *N*1-AcSpd accelerated cell cycle progression. Previously, acetylated polyamines were described primarily as the major polyamine species exported from the cell. Here, we demonstrate that *N*1-AcSpd influences cell fate decisions via increased proliferation. Thus, our study explains why elevated polyamine levels and low translation rates are not mutually exclusive during stem cell maintenance. Instead, they regulate different aspects of cell fate. While low translation rates favor quiescence *in vivo*, enhanced cell cycle progression ensures stem cell self-renewal upon activation. The upregulation of the acetylated polyamines upon HFSC activation by depilation supports this model. Over the long term, *N*1-AcSpd treatment could be a viable intervention to tackle age-associated diseases caused by a decline in tissue homeostasis due to stem cell exhaustion.

## MATERIALS AND METHODS

### Mouse husbandry

Animals (*Mus musculus*) were housed on a 12 h light–12 h dark cycle with *ad libitum* access to food under pathogen-free conditions in individually ventilated cages. All animals were kept in C57BL/6J background. Animal care and experimental procedures were in accordance with the institutional and governmental guidelines.

### Depilation

Animals were anesthetized with isoflurane (cp-pharma) and dorsal skin was depilated using hair removal strips. The mice were killed on day 9 after depilation and tissue biopsies were collected for histological analysis and polyamine measurements as described below. Male mice at the age of 6 months (young) and 24 months (old) were used for the experiment.

### Immunofluorescence staining

Depilated back skin was fixed using 4% paraformaldehyde (PFA), embedded in paraffin and sectioned. The sections were de-paraffinized using a graded alcohol series, and target retrieval was performed in Target Retrieval Solution (DAKO) pH 9 in a pressure cooker at 110°C for 2 min. After blocking in 5% bovine serum albumin, primary antibody (Krt14, Progen, GP-CK14, 1:500) diluted in Dako Antibody Diluent was incubated with the samples overnight at 4°C. Bound primary antibody was detected by incubation with Alexa Fluor 657-conjugated secondary antibodies (Invitrogen). Nuclei were counterstained with DAPI (Invitrogen). After washing, the slides were mounted in Elvanol. All fluorescence images were collected by laser scanning confocal microscopy (SP8X; Leica) with Leica Application Suite software (LAS X version 2.0.0.14332), using a 40× immersion objective. Images were acquired using sequential scanning of frames of 1 µm thick confocal planes (pinhole 1). Afterwards, planes were projected as a maximum intensity confocal stack. Images were collected with the same settings for all samples within an experiment.

### Isolation of epidermal cells

Isolation of epidermal cells from mice of both sexes in telogen stage [postnatal day (P)21–25; P46–60] was performed as described previously (Chacón-Martínez et al., 2017). In brief, mouse skin was incubated with 0.8% trypsin (Thermo Fisher Scientific) for 50 min at 37°C. The skin was transferred to 8 ml KGM medium and the epidermis was separated from the dermis. After centrifugation (600 ***g*** for 5 min), the keratinocytes were resuspended in ice cold KGM medium (see below) and embedded in growth factor-reduced Matrigel (Corning Inc.). Cells isolated from independent donors represent biological replicates. Analysis of the stem cell ratio, EdU incorporation, cell cycle progression, and annexin V staining was performed in technical duplicates. The mean of the technical duplicates is plotted. All analyses were performed with at least three biological replicates.

### Culture of HFSC organoids

HFSC organoids were cultured in KGM medium [MEM medium (Spinner's modification, Sigma-Aldrich), supplemented with 8% fetal bovine serum (chelated, Thermo Fisher Scientific), penicillin/streptavidin (Thermo Fisher Scientific), L-glutamine (Thermo Fisher Scientific), insulin (5 µg/ml, Sigma-Aldrich), hydrocortisone (0.36 µg/ml, Calbiochem), EGF (10 ng/ml, Sigma-Aldrich), transferrin (10 µg/ml, Sigma-Aldrich), phosphoethanolamine (10 µM, Sigma-Aldrich), ethanolamine (10 µM, Sigma-Aldrich) and CaCl_2_ (14.5 µM, Sigma-Aldrich)]. 5 µM Y27632, 20 ng/ml mouse recombinant VEGF, 20 ng/ml human recombinant FGF2 (all Miltenyi Biotech) were added to KGM medium. The cells were grown at 37°C in 5% CO_2_.

The medium was changed three times per week. The following compounds were added each time the medium was changed: 2-difluoromethylornithine hydrochloride (DFMO; 2761, R&D Systems); diminazene aceturate (DA; D7770, Sigma-Aldrich); MDL72527 (M2949, Sigma-Aldrich); *N*1-acetylspermidine hydrochloride (9001535, Cayman Chemical); *N*1-acetylspermine trihydrochloride (01467, Sigma-Aldrich); *N*1,*N*11-diethylnorspermine (DENSpm) tetrahydrochloride (0468, Tocris); putrescine dihydrochloride (P5780, Sigma-Aldrich). Unless indicated otherwise, 3D–3C organoids were treated for the entire duration of the experiment. The concentrations were determined by assessing concentration curves – the lowest concentration that had an effect on cell fate or the highest concentration without overt toxicity were chosen for further experiments. The concentrations used are indicated in the respective figures. After sorting of pure populations, the cells were recovered for 2 days and treated for the last 5 days of the experiment. Before puromycin incorporation, the cells were cultured for 2 weeks and treated for the last 72 h.

### Live-cell number

Sorted cells were cultured in 3D–3C organoids for 7 days and treated for the last 5 days before Matrigel droplets were degraded in 0.5% trypsin and 0.5 mM EDTA in PBS for 8 min at 37°C. Trypsin was neutralized using cold KGM. The live-cell number of at least three biological replicates was determined using a Neubauer hemocytometer. Images of organoid cultures were acquired using the EVOS FL Auto 2 Imaging System (ThermoFisher Scientific) with an 4× objective. The scale bar is 650 µm.

### Cell maintenance

NIH3T3 fibroblasts were obtained from ATCC^®^ (CRL-1658). The cells were grown in DMEM containing 4.5 g/l glucose supplemented with 10% fetal bovine serum and penicillin/streptavidin (all Thermo Fisher Scientific) at 37°C in 5% CO_2_ on non-coated tissue culture plates.

### Colony formation assay

NIH3T3 fibroblasts were seeded on collagen G-coated (30 µg/ml in PBS; Biochrom AG) tissue culture plates. The cells were grown at 37°C in 5% CO_2_ for 2 days before proliferation was inhibited with 4 µg/ml mitomycin C (Sigma-Aldrich).

HFSCs were grown in 3D–3C organoids for 2 weeks and analyzed by flow cytometry. 4000 cells were seeded per 6-well (technical triplicates) on the fibroblast layer in MEM/HAM's F12 (FAD) medium with low Ca^2+^ (50 µM; Biochrom AG), supplemented with 10% fetal bovine serum (chelated, Thermo Fisher Scientific), penicillin/streptomycin (penicillin, 100 U/ml; streptomycin, 0.1 mg/ml; Thermo Fisher Scientific), L-glutamine (2 mM; Thermo Fisher Scientific), ascorbic acid (50 µg/ml; Sigma-Aldrich), adenine (0.18 mM; Sigma-Aldrich), insulin (5 µg/ml; Sigma-Aldrich), hydrocortisone (0.5 µg/ml; Sigma-Aldrich), EGF (10 ng/ml; Sigma-Aldrich) and cholera enterotoxin (10 ng/ml, Sigma-Aldrich). The cells were grown without treatment for 2–3 weeks at 32°C in 5% CO_2_ until colonies were formed. Remaining fibroblasts were removed using 0.25% trypsin-ETDA (Thermo Fisher Scientific) for 2 min at 37°C. Trypsin was stopped using supplemented DMEM. After washing the plates with PBS, keratinocytes were fixed with 4% PFA (Sigma-Aldrich) for 15 min at room temperature (RT). The cells were washed with PBS twice and the colonies were stained using 1% Crystal Violet (Sigma-Aldrich in PBS) for 1 h at RT on an orbital shaker. The wells were washed with tap water until no stain was released and air dried. The plates were scanned and the number of colonies was counted manually.

### Flow cytometry

Matrigel droplets were degraded in 0.5% trypsin, 0.5 mM EDTA in PBS for 8 min at 37°C. Trypsin was neutralized using cold KGM. After centrifugation for 5 min at 600 ***g***, cells were washed with FACS buffer (2% FBS, 2 mM EDTA in PBS). Surface marker staining was performed for 30 min on ice with the following antibodies: CD34-eFluor 660 (eBioscience, clone RAM34, 1:100) and ITGA6-PE/Cy7 (Biolegend, clone GoH3, 1:1000) for sorting or CD34-eFluor 660 (eBioscience, clone RAM34, 1:100) and ITGA6-Pacific Blue (Biolegend, clone GoH3, 1:200) for analysis. Freshly isolated keratinocytes were stained additionally with Sca1-Pacific Blue (Biolegend, clone D7, 1:400). 7AAD or propidium iodide (PI) was used to exclude dead cells. Cells were analyzed on FACSCantoII (BD Biosciences) or sorted on FACSAria IIIu Svea and FACSAria Fusion sorters (BD Biosciences). Sorted cells were collected in 15 ml tubes containing KGM at 4°C.

### EdU incorporation and detection

Cell proliferation was assessed using the Click-iT™ Plus EdU Alexa Fluor™ 488 Flow Cytometry Assay Kit (Thermo Fisher Scientific) following the manufacturer's instructions. Cells were grown for 9–14 days in 3D–3C culture before 10 µM EdU was added to the medium and incubated for 2 h or 24 h. Matrigel droplets were degraded, the cells were washed with PBS and subsequently stained with fixable LIVE/DEAD-violet dye (Thermo Fisher Scientific, 1:500) for 20 min at RT. The cells were washed with FACS buffer and surface marker staining was performed for 30 min on ice using CD34-eFluor 660 (eBioscience, clone RAM34, 1:100) and ITGA6-PE/Cy7 (Biolegend, clone GoH3, 1:1000). The cells were washed with 1% bovine serum albumin (BSA) in PBS, fixed with 4% PFA for 10 min at RT and permeabilized using Click-iT saponin-based permeabilization buffer for 15 min at RT. The EdU reaction cocktail was prepared following the manufacturer's instructions and incubated for 30 min at RT in the dark after washing the cells. The cells were analyzed on FACSCantoII.

### Cell cycle analysis

Cell proliferation was assessed using the Click-iT™ Plus EdU Alexa Fluor™ 488 Flow Cytometry Assay Kit (Thermo Fisher Scientific) as described above. Cells were grown for 2 weeks in 3D–3C culture before 10 µM EdU was added to the medium and incubated for 2 h. Matrigel droplets were degraded, the cells were washed with PBS and stained with fixable Zombie NIR dye (Biolegend, 1:500) for 20 min at RT. The cells were washed, fixed and permeabilized, and the EdU reaction cocktail was incubated with the sample as described above. Subsequently, the cells were permeabilized using 0.1% Triton X-100 in PBS for 15 min at RT and stained with FxCycle Violet (Thermo Fisher Scientific, 1:500) for 30 min at RT. The cells were analyzed on FACSCantoII without further washing.

### Annexin V staining

Cells from four independent donors representing four biological replicates were cultured for 13 days. DENSpm treatment was performed for the last 24 h. The supernatant was collected and stained together with the embedded cells to include detached cells. Matrigel droplets were degraded in 0.5% trypsin, 0.5 mM EDTA in PBS for 8 min at 37°C. Trypsin was neutralized using cold KGM. After centrifugation for 5 min at 600 ***g*** cells were washed with FACS buffer. Surface marker staining was performed for 30 min on ice using CD34-eFluor 660 (eBioscience, clone RAM34, 1:100) and ITGA6--acific Blue (Biolegend, clone GoH3, 1:200). Cells were washed with PBS and stained with annexin V-AF488 (ThermoFisher Scientific, 1:20 in 100 µl annexin-binding buffer per sample) for 15 min at RT. 100 µl annexin-binding buffer containing PI was added per sample. The samples were analyzed on FACSCantoII.

### Puromycin incorporation

Cells were incubated with 10 µg/ml puromycin in the medium for exactly 10 min at 37°C. The cells were washed with PBS and collected in RIPA buffer (50 mM Tris-HCl pH 7.4, 120 mM NaCl, 0.1% SDS, 1% NP40, 0.5% sodium deoxycholate) with proteinase and phosphatase inhibitors (Roche Diagnostics GmbH). Sorted cells were recovered for 30 min at 37°C before puromycin was incorporated. Cells from at least three independent donors representing at least three biological replicates were used for puromycin incorporation. All treatments were performed for the final 72 h of culture. This treatment duration did not affect the proportion of HFSCs, which was confirmed by FACS analysis.

### Western blot analysis

Protein concentration of cell lysates was determined using the Pierce™ BCA protein assay kit according to the manufacturer's instructions (ThermoFisher Scientific). Samples were subsequently subjected to SDS-PAGE and blotted on a nitrocellulose membrane. The following antibodies were used in 5% low-fat milk in TBS with 0.05% Tween 20 (w/v in ddH_2_O): puromycin (mouse, Millipore, 12D10, 1:10,000), β-actin (mouse, Sigma, AC-74, 1:25,000), β-actin (rabbit, Cell Signaling Technologies, 4967, 1:50,000). After incubation with HRP-conjugated secondary antibody (Invitrogen, 1:5000), the blot was developed using ECL solution (Merck Millipore). Films were used for detection (Amersham Biosciences).

### RNA isolation and quantitative RT-PCR

Sorted cells were collected in QIAzol and frozen in liquid nitrogen. Cells from three independent donors representing three biological replicates were used for further analysis. Samples were subjected to three freeze–thaw cycles (liquid nitrogen and 37°C water bath). Half of the total volume of QIAzol was added per sample. After incubation for 5 min at RT, 200 µl chloroform were added per 1 ml QIAzol. The samples were vortexed, incubated for 2 min at RT, and centrifuged at 10,000 ***g*** and 4°C for 15 min. The aqueous phase was mixed with an equal volume of 70% ethanol and transferred to a RNeasy Mini spin column (Qiagen). Total RNA was isolated with the RNeasy Mini Kit (Qiagen) according to the manufacturer's instructions. cDNA was generated using the iScript cDNA Synthesis Kit (Bio-Rad Laboratories Inc.). Quantitative RT-PCR was performed using Power SYBR Green master mix (Applied Biosystems) on a ViiA 7 Real-Time PCR System (Applied Biosystems). Primer sequences are listed in Table S2.

### Preparation of 3′ RNA-seq libraries

Sorted cells were collected in QIAzol and frozen in liquid nitrogen. Untreated and treated cells from four independent donors representing four biological replicates were used for further analysis. Total RNA was isolated as described above. 50 ng RNA per sample were used for cDNA synthesis with Maxima H Minus reverse transcriptase (Thermo Fisher Scientific). During reverse transcription, unique barcodes including unique molecular identifiers (UMI) were attached to each sample. After cDNA synthesis, all samples were pooled and processed in one single tube. DNA was purified using AmpureXP beads (Beckman Coulter) and the eluted cDNA was subjected to Exonuclease I treatment (New England Biolabs). cDNA was PCR-amplified for 12 cycles and subsequently purified. After purification, cDNA was tagmented in 5 technical replicates of 1 ng cDNA each using the Nextera XT Kit (Illumina), according to the manufacturer's instructions. The final library was purified and concentration and size were validated by Qubit and High Sensitivity TapeStation D1000 analyses. A detailed protocol for library preparation is available on request. Sequencing was carried out on an Illumina NovaSeq system.

### Bioinformatic analysis of 3′ RNA-seq data

The raw reads were demultiplexed and the UMI-tag was added to each read name using FLEXBAR (Dodt et al., 2012). The second end of the read-pairs were mapped using Kallisto creating pseudo-alignments to the mouse reference genome mm10 (Bray et al., 2016). UMI-tags were assigned using BEDTools intersect and bash re-formatting (Quinlan and Hall, 2010). An R script was used to count the unique UMI-tags per gene. Differential gene expression was calculated using the R package Deseq2 (Love et al., 2014). Only genes with an average of more or equal to 5 reads over all samples were used for the analysis. Only genes with an adjusted *P*-value ≤0.05 (α6+/CD34- versus α6+/CD34+; untreated or treated) are shown in the heatmap. Venn diagrams were generated using the R package VennDiagram. Functional enrichment analysis was performed using the Metascape tool (http://metascape.org) (Zhou et al., 2019). Only genes with a *P*-value <0.05 and a log2FC >±0.5 (α6+/CD34− versus α6+/CD34+; untreated or treated) were defined as differentially expressed and included in the functional enrichment analysis.

### Polysome profiling

3D–3C organoids were cultured for 2 weeks. Matrigel droplets were degraded in 0.5% trypsin, 0.5 mM EDTA in PBS for 8 min at 37°C. Trypsin was neutralized using cold KGM. After centrifugation for 5 min at 600 ***g***, cells were washed with cold PBS and the pellet was frozen in liquid nitrogen. The pellet was resuspended in 300 µl lysis buffer (10 mM NaCl, 10 mM MgCl_2_, 10 mM Tris-HCl pH 7.5, 1% triton X-100, 1% sodium deoxycholate, 1 mM DTT and 0.1 mg/ml cycloheximide) and incubated for 30 min on ice. The samples were centrifuged for 10 min at 4°C and 21,000 ***g*** for clearance.

To prepare sucrose gradients, 15% (w/v) and 50% (w/v) sucrose solutions were prepared in basic lysis buffer (10 mM NaCl, 10 mM MgCl_2_, 10 mM Tris-HCl pH 7.5, 1 mM DTT, 0.1 mg/ml cycloheximide). Linear sucrose gradients were produced using a Gradient Master (Biocomp). The samples were loaded on the gradient and centrifuged using an Optima L-100 XP Ultracentrifuge (Beckman Coulter) and the SW41Ti rotor for 2 h at 39,000 ***g*** and 4°C. To analyze the sample on the gradient, absorbance at 254 nm was measured and recorded (Econo UV monitor EM-1, Biorad).

### Sample preparation for ribosome footprinting

3D–3C organoids were cultured for 2 weeks and treated for the last 72 h. The cells were sorted and frozen in liquid nitrogen. Untreated and treated cells from two independent donors representing two biological replicates were used for further analysis. The cells were incubated in 300 µl lysis buffer (10 mM NaCl, 10 mM MgCl_2_, 10 mM Tris-HCl pH 7.5, 1% Triton X-100, 1% sodium deoxycholate, 1 mM DTT and 0.1 mg/ml cycloheximide) for 30 min on ice. The samples were centrifuged for 10 min at 4°C at 21,000 ***g***. The concentration was determined by NanoDrop and 1 µg of RNA was subjected to the Direct-zol™ RNA Microprep Kit (Zymo Research) to isolate total RNA according to the manufacturer's instructions, which was subsequently subjected to RNA-sequencing.

The rest of the sample was incubated with 2.5 µl Ambion™ RNase I (Thermo Fisher Scientific) and 2.5 µl RNase S7 (Roche) for 1 h at RT. The reaction was stopped by the addition of 3 µl RNasin Plus (Promega Corporation). Ribosomal proteins were digested by proteinase K in proteinase K buffer (50 mM NaCl, 10 mM Tris-HCl pH 7.4, 5 mM EDTA, 5% SDS) containing GlycoBlue (0.75 mg/ml; Thermo Fisher Scientific) for 1 h at 65°C. 1 volume acid-phenol:chloroform (Thermo Fisher Scientific) was added and the samples were centrifuged for 10 min at 4°C and 12,000 ***g***. The supernatant was collected, 1 volume of chloroform was added and the samples were centrifuged for 10 min at 4°C and 12,000 ***g***. This step was repeated twice. The supernatant was mixed with 3 volumes of cold ethanol containing 100 mM sodium acetate and incubated at −80°C overnight. The samples were centrifuged for 1 h at 4°C and 12,000 ***g***. 1 volume of cold ethanol was added to the pellet before centrifugation for 10 min at 4°C and 12,000 ***g***. The supernatant was discarded and the pellet was air dried and stored at −80°C until further use.

### Preparation of sequencing libraries for ribosome footprinting

For RNA-seq, libraries were prepared using the NEBNext^®^ Ultra™ RNA Library Preparation Kit for Illumina (NEB) including hybridization-based ribo-depletion according to the manufacturer's instructions. Library validation and quantification were performed using TapeStation analysis (Agilent). Equimolar amounts of the library were pooled. The pool was quantified using the KAPA Library Quantification Kit (Peqlab) and the ABI Prism^®^ 7900HT Sequence Detection System (Applied Biosystems) and sequenced on an Illumina NovaSeq6000 sequencer.

For Ribo-sequencing, the fragmented RNA pellets were resuspended in nuclease-free water and treated with T4 polynucleotide kinase (NEB) for 90 min at 37°C. Size selection of the fragmented RNA was performed using PAGE. Gel slices in the range of 24–36 nt were excised using X-Tracta Tips (Biozym). After purification, RNA fragments were resuspended in nuclease-free water. Sequencing libraries were prepared using the Small RNA-Seq Library Prep Kit (Lexogen) according to the manufacturer's instructions. Amplification was undertaken using 16 PCR cycles. The pool of all libraries was size-selected using the BluePippin device (SAGE-Science) with a 3% DF Marker Q3 Kit (SAGE-Science). The range of 135–180 bp was selected. The pool was quantified using the KAPA Library Quantification Kit (Peqlab) and the ABI Prism^®^ 7900HT Sequence Detection System (Applied Biosystems) and sequenced on an Illumina NovaSeq6000 sequencer.

Library preparation and sequencing for RNA-sequencing and Ribo-sequencing was performed at the Cologne Center for Genomics.

### Bioinformatic analysis of Ribo-sequencing data

The adapters were trimmed using flexbar (Roehr et al., 2017). Reads aligning to rRNA or tRNA were removed with bowtie (Langmead et al., 2009). The reads were aligned to the reference genome mm10 and the transcriptome using STAR (Dobin et al., 2013). The P-site offset and meta-profile were determined with the R package riboWaltz (Lauria et al., 2018). Actively translated open reading frames (ORFs) were identified using the Python package RiboCode (Xiao et al., 2018). Differentially translated genes were determined using the R package Riborex (Li et al., 2017). Only genes with an average of more or equal to 1 read over all samples were used for further analysis. Functional enrichment analysis was performed using the Metascape tool (http://metascape.org; Zhou et al., 2019). For Figs 1G, 4J; Fig. S5H, all translated genes were used for functional enrichment analysis independently of the *P*-value. Only genes with a *P*-value <0.05 (α6+/CD34− versus α6+/CD34+; untreated or treated) were defined as differentially translated and used for functional enrichment analysis shown in Fig. S1K,L, Fig. 4K and Fig. S5I,J.

### Sample preparation for polyamine extraction from cells

Matrigel droplets were degraded in 0.5% trypsin and 0.5 mM EDTA in PBS for 8 min at 37°C. Trypsin was neutralized using cold KGM. After centrifugation for 5 min at 600 ***g***, cells were washed with PBS. Sorted cells were centrifuged (1500 ***g*** for 10 min) and washed with PBS. The pellet was flash frozen in liquid nitrogen. The cells were lysed in ddH_2_O by freeze–thaw cycles and the protein concentration was determined using the Pierce™ BCA protein assay kit (Thermo Fisher Scientific).

### Sample preparation for polyamine extraction from epidermis

Depilated skin (anagen) and control skin (telogen) from four young and four old male mice was collected and incubated on collagenase (400 U/ml in 50 mM Tris base, 5 mM CaCl_2_, pH 7.4; Sigma-Aldrich) at 37°C for 45 min with the dermal side down. The dermis was separated from the epidermis using a scalpel and the epidermis was frozen in liquid nitrogen. Tissue was disrupted using a TissueLyser II (Qiagen) at 20–25 Hz. The powder was transferred to a fresh tube and solved in ddH_2_O. After four freeze–thaw cycles, the protein concentration was determined using the Pierce™ BCA protein assay kit (ThermoFisher Scientific). In some samples, the protein concentration was too low for further processing.

### Polyamine extraction

Polyamines were extracted by Bligh and Dyer extraction (Noga et al., 2012; Pieragostino et al., 2015). In brief, a methanol:chloroform mixture of 2:1 (v/v) was added to a volume of cell lysate that corresponded to 100 µg of protein and then was incubated for 1 h at 4°C. Samples were centrifuged for 5 min at 3000 ***g*** at 4°C and the liquid phase was transferred and dried in a SpeedVac Vacuum Concentrator (Genevac). Samples were reconstituted with 15 µl aqueous acetonitrile 2:3 mixture (v/v) and 10 µl were injected into the LC-MS system.

### Targeted analysis of polyamines by LC-MS

The identification of polyamines was performed on a triple stage quadrupole (TSQ Altis, ThermoFisher Scientific GmbH) coupled with a binary pump system (Vanquish, ThermoFisher Scientific GmbH). Polyamine species were separated using a reverse column (Xselect column, 2.1×100 mm, 2.5 µm) using solvent A (water with 0.1% formic acid) and B (acetonitrile with 0.1% formic acid) as previously reported (Häkkinen et al., 2008, 2013; Sánchez-López et al., 2009).

The gradient started from 0.1% eluent B and ramped to 0.3% eluent B in 0.5 min. It ramped further to 0.5% eluent B in 0.5 min and to 1% B in the next 0.5 min. The gradient increased to 2% eluent B in the next minute, then it ramped to 5% eluent B in 1 min. In the following 3 min it went to 95% eluent B and stayed constant for 3 min. Afterwards the gradient decreased to 0.1% eluent B in 2 min and stayed constant for 3 min adding up to a total time of 15 min. The column was heated to 30°C using a flow rate of 100 µl/min. The LC system was flushed in between runs with isopropanol:water 75:25 (v/v) with 0.1% formic acid.

Polyamines were detected using heated electrospray ionization (HESI) with the following parameters: 10 (a.u.) sheath gas, 5 (a.u.) auxiliary, 200°C transfer ion capillary and 3 kV for spray voltage.

Relative quantification was performed using selected ion monitoring chromatogram mode (SIM-Q1) in positive ion mode using a scan rate of 1000 Da/sec, Q1 resolution was set to 0.7 *m/z* and calibrated RF lenses were used. The following ions were monitored: ornithine→133.17, putrescine→89.17, spermidine→146.23, spermine→203.32, *N*1-acetylspermidine→188.25, *N*1-acetylspermine→245.34. The relative response for each polyamine was calculated using spermidine-(butyl-d8) as internal standard. A detailed description of the detected metabolites is shown in Table S3.

### Statistical analysis

Data are presented as mean±s.e.m. or mean±s.d. Biological replicates represent independent donors for 3D–3C organoids or independent mice (*n*). Statistical significance was calculated using GraphPad Prism 8 (GraphPad Software). The statistical test used is indicated in the respective figure legend. Significance levels are *P<0.05, **P<0.01, ***P<0.001 versus the respective control.

### Software

Graphs were generated using GraphPad Prism 8 (GraphPad Software). For western blot analysis, the intensity of the bands was quantified using Fiji (Schindelin et al., 2012). Flow profiles were recorded and exported with the FACSDiva™ software (BD Biosciences). FlowJo™ Software v.10.0.8 (FlowJo LLC) was used for the analysis of flow cytometry data. Functional enrichment analysis was performed using the Metascape tool (http://metascape.org; Zhou et al., 2019).

## Supplementary Material

Supplementary information

Reviewer comments
