# Supplementary material for: N1-acetylspermidine is a determinant of hair follicle stem cell fate
Source: J Cell Sci. 2021 May 11;134(9):jcs252767. doi: 10.1242/jcs.252767 (PMC8182411; doi:10.1242/jcs.252767)
Supplement: Supplementary information [file joces-134-252767-s1.pdf]

**Table S1: Expression of the genes associated with the GO term "cell division" in untreated and *N*1-AcSpd treated cells.** The ratio of the log2FC according to the 3' RNA-sequencing data in α6+/CD34+ vs. α6+/CD34- cells is shown.

| Gene names | untreated | *N*1-AcSpd |
|---|---|---|
| Anapc5 | 0,253 | 0,507 |
| Aspm | 0,562 | 0,747 |
| Aurka | 0,332 | 0,664 |
| Bub1 | 0,463 | 0,732 |
| Ccnb1 | 0,353 | 0,646 |
| Ccnb2 | 0,482 | 0,823 |
| Ccnd1 | -0,474 | -0,626 |
| Ccnf | 0,368 | 0,571 |
| Ccng1 | -0,493 | -0,652 |
| Ccp110 | 0,550 | 0,994 |
| Cdc20 | 0,334 | 0,577 |
| Cdc23 | 0,480 | 0,539 |
| Cdca3 | 0,449 | 0,716 |
| Cdca8 | 0,319 | 0,524 |
| Cenpa | 0,310 | 0,665 |
| Cenps | 0,301 | 0,510 |
| Cep164 | 0,441 | 0,659 |
| Cep55 | 0,397 | 0,847 |
| Cit | 0,260 | 0,859 |
| Ckap5 | 0,298 | 0,521 |
| Dixdc1 | -0,520 | -0,811 |
| Dsn1 | 0,394 | 0,632 |
| E2f8 | 0,403 | 0,564 |
| Ect2 | 0,448 | 0,799 |
| Fbxo5 | 0,434 | 0,772 |
| Hmga2 | -0,089 | -0,708 |
| Incenp | 0,499 | 0,612 |
| Itgb3bp | 0,360 | 0,539 |
| Kif20a | 0,480 | 0,553 |
| Knl1 | 0,349 | 0,503 |
| Knstrn | 0,475 | 0,634 |
| Lig4 | 0,018 | -0,528 |
| Mad2l1 | 0,365 | 0,605 |
| Mapre2 | -0,404 | -0,664 |
| Ncapd2 | 0,441 | 1,136 |
| Ncapg2 | 0,453 | 0,619 |
| Nde1 | 0,342 | 0,510 |
| Nek2 | 0,428 | 0,814 |
| Nsl1 | 0,443 | 0,508 |
| Numa1 | 0,420 | 0,624 |
| Pdgfb | 0,682 | 0,953 |
| Plk1 | 0,349 | 0,587 |
| Ppp2r2d | -0,428 | -0,596 |
| Pstpip1 | -0,612 | -0,740 |
| Rbbp8 | 0,416 | 0,578 |

Journal of Cell Science • Supplementary information

| Gene names | untreated | *N*1-AcSpd |
|---|---|---|
| Septin8 | -0,284 | -0,557 |
| Sgo1 | 0,353 | 0,792 |
| Sgo2a | 0,324 | 0,581 |
| Smc2 | 0,378 | 0,616 |
| Tacc3 | 0,337 | 0,538 |
| Terf1 | 0,372 | 0,504 |
| Tpx2 | 0,459 | 0,580 |
| Ube2c | 0,385 | 0,660 |
| Wee1 | 0,397 | 0,665 |

**Table S2: Primer sequences for quantitative RT-PCR.**

| Primer/gene name | Sequence (5'→ 3') |
|---|---|
| msβ-actin_fwd<br>msβ-actin_rev | TCAAGATCATTGCTCCTCCTG<br>TACTTCTGCTTGCTGATCCAC |
| msGAPDH_fwd<br>msGAPDH_rev | GGTGTGAACGGATTTGGCCGTATTG<br>CCGTTGAATTTGCCGTGAGTGGAGT |
| msCD34_fwd<br>msCD34_rev | TGAGATGACATCACCCACCG<br>GCCAACCTCACTTCTCGGAT |
| msSox9_fwd<br>msSox9_rev | AGGAAGCTGGCAGACCAGTA<br>TCCACGAAGGGTCTCTTCTC |
| msTcf3_fwd<br>msTcf3_rev | CTCAGCAGCAAATCCAAGAGGCAGAG<br>TGGGAAGACGCAGGGCTATCACAAG |
| msLhx2_fwd<br>msLhx2_rev | ATCGACGAGATGGACCGCA<br>TCACTGCTGATGGACGGC |
| msNfatc1_fwd<br>msNfatc1_rev | GGTGCTGTCTGGCCATAACT<br>CCAGGGAATTTGGCTTGCAC |
| msId2_fwd<br>msId2_rev | ATCCCCCAGAACAAGAAGGT<br>TGTCCAGGTCTCTGGTGATG |
| msDkk3_fwd<br>msDkk3_rev | ATGCTATGCACCCGAGACAG<br>GAACAGCAGGCCTCTTTGGA |

**Table S3: Detailed description of the detected metabolites.**

| Name | Elemental composition | Retention time (min) | Mass (amu) | $[M+H]^+$ *(m/z)* |
|---|---|---|---|---|
| ornithine | $C_5H_{12}N_2O_2$ | 2.73 | 132.089 | 133.097 |
| putrescine | $C_4H_{12}N_2$ | 2.41 | 88.099 | 89.107 |
| spermidine | $C_7H_{19}N_3$ | 2.22 | 145.157 | 146.165 |
| spermine | $C_{10}H_{26}N_4$ | 2.46 | 202.215 | 203.223 |
| *N*1-acetylspermidine | $C_9H_{21}N_3O$ | 3.14 | 187.167 | 188.175 |
| *N*1-acetylspermine | $C_{12}H_{28}N_4O$ | 3.05 | 244.225 | 245.233 |

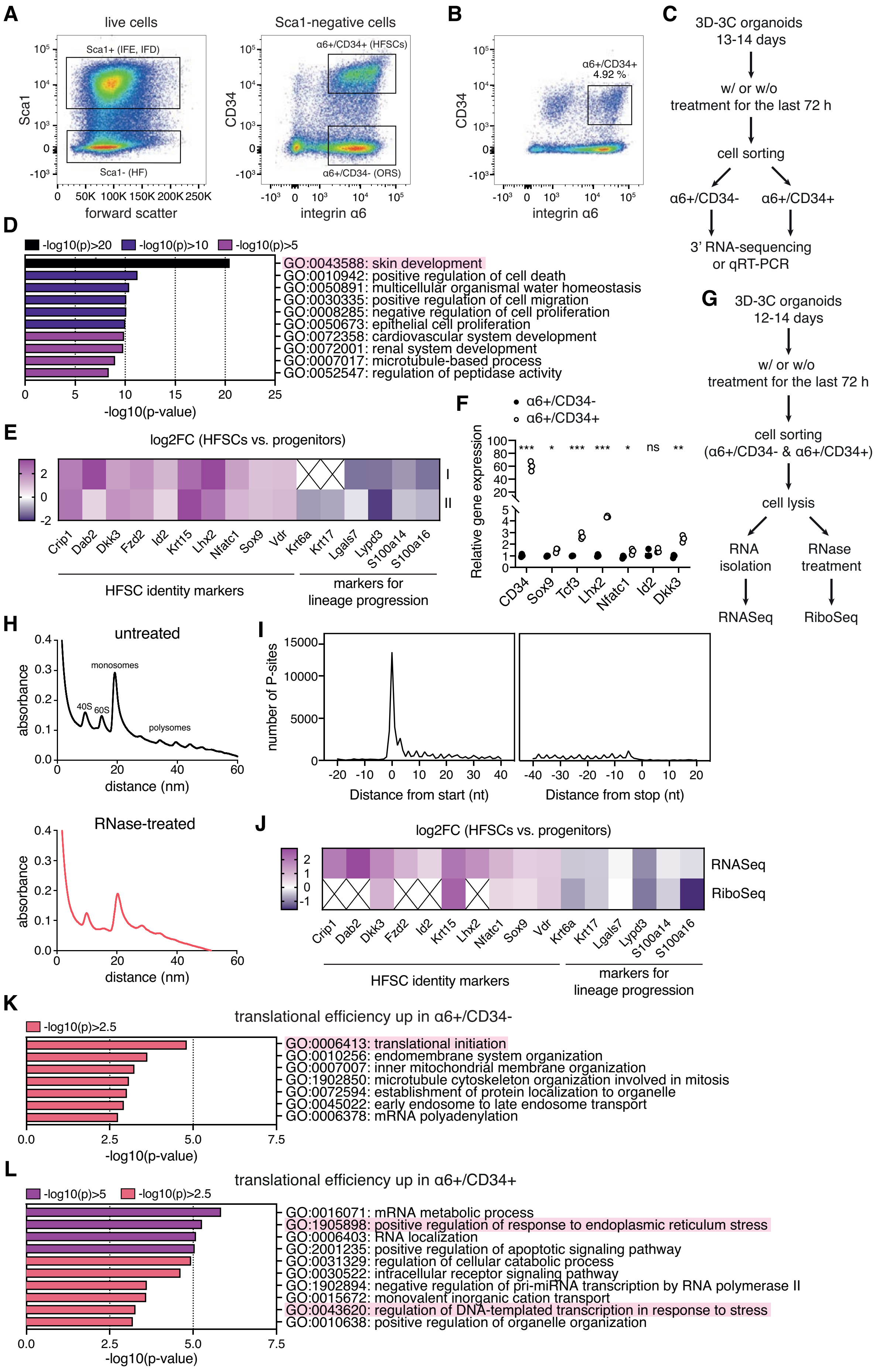


Journal of Cell Science • Supplementary information

**Figure S1: 3' RNA-sequencing confirms cell identity in the 3D-3C organoids and ribosome foot printing reveals differential translation in progenitors compared to HFSCs.** (**A**) Dot plot showing the gating strategy for sorting of freshly isolated epidermel cells. Live cells were gated according to Sca1-expression. Sca1-negative cells were separated into HFSCs and ORS cells. (**B**) Representative dot plot showing the different populations of freshly isolated epidermis cells using integrin $\alpha6$ and CD34 as markers. (**C**) Schematic representation of the workflow for quantitative RT-PCR (qRT-PCR) and 3' RNA-sequencing sample collection. (**D**) GO term analysis of differentially expressed genes (p-value < 0.05, log2FC > ±0.5) from 3' RNASeq experiment comparing $\alpha6$+/CD34- progenitor cells with $\alpha6$+/CD34+ stem cells (biological process, metascape.org). (**E**) Heatmap showing mean values of log2FC of different HFSC identity markers (left) and markers for lineage progression (right) based on published RNA-sequencing data from Chacón-Martínez et al. (2017) (top; **I**), compared to the 3' RNA-sequencing results obtained in this study (bottom, **II**). The ratio of the log2FC in HFSCs vs. progenitor cells is displayed. (**F**) Stem cell marker gene expression in sorted $\alpha6$+/CD34- and $\alpha6$+/CD34+ cells determined by qRT-PCR. mean±s.e.m. (n=3). Statistical significance was calculated by unpaired *t*-test. two-tailed p-values: *** p<0.001, ** p<0.01, * p<0.05, ns: not significant. (**G**) Schematic representation of the workflow for RNASeq and RiboSeq sample collection. (**H**) Polysome profiling of untreated and RNase-treated samples. (n=1). (**I**) Representative meta-profile based on P-site mapping around the start and the stop codon of annotated coding sequences. (n=2 for each condition). (**J**) Heatmap showing mean values of log2FC of different HFSC identity markers (left) and markers for lineage progression (right) comparing RNASeq (top) and RiboSeq (bottom). The ratio of the log2FC in HFSCs vs. progenitor cells is displayed. A crossed cell indicates that the respective transcript was not detected in at least one sample in the RiboSeq experiment. (n=2 for each cell type). (**K**) GO term analysis of differentially translated genes between cell types (p<0.05) with higher translational efficiency in $\alpha6$+/CD34- cells (biological process, metascape.org). (**L**) GO term analysis of differentially translated genes between cell types (p<0.05) with higher translational efficiency in $\alpha6$+/CD34+ cells (biological process, metascape.org). (**F**,**H**,**I**,**J**) n: biological replicates.

Journal of Cell Science • Supplementary information

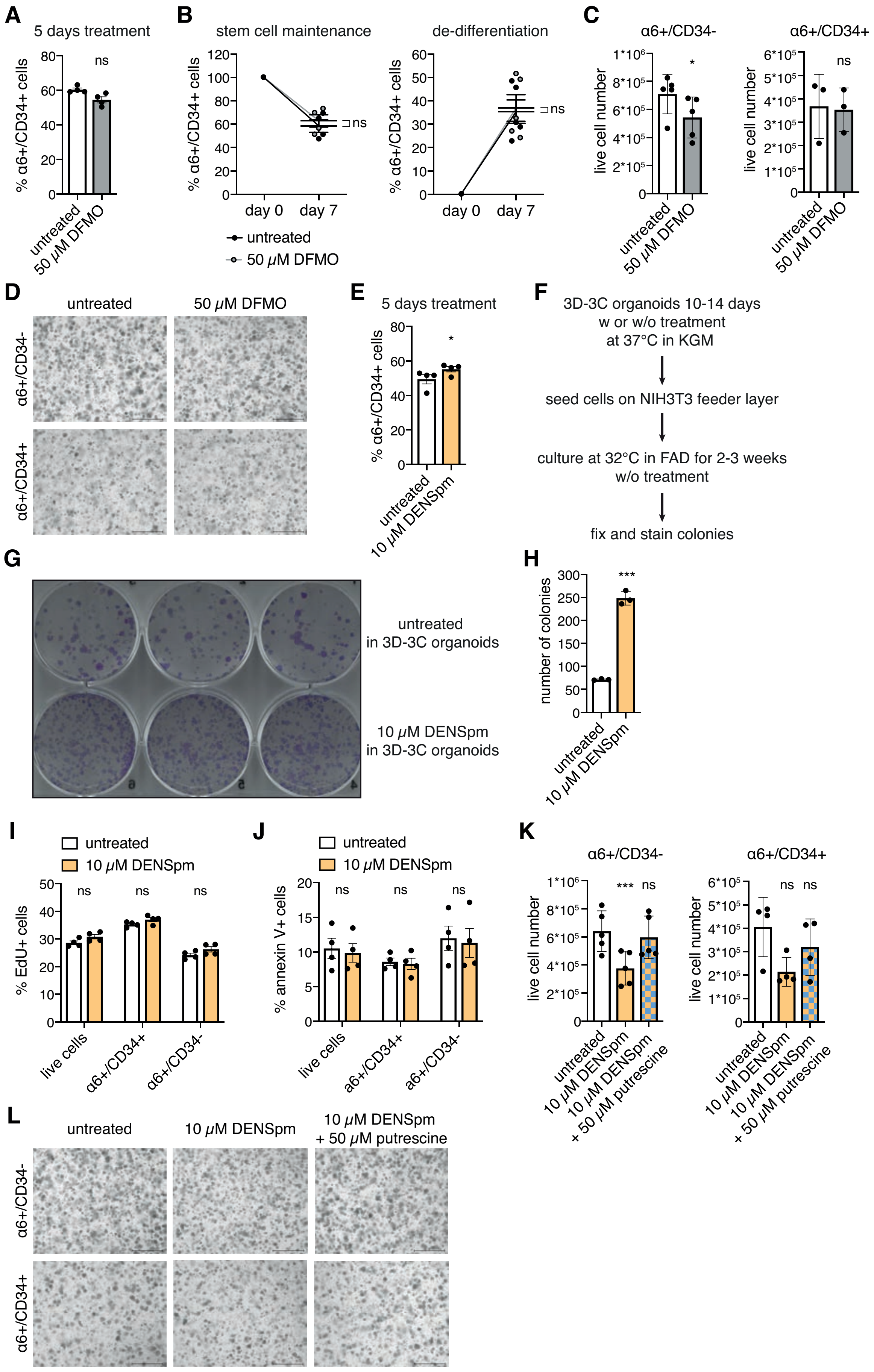


Journal of Cell Science • Supplementary information

**Figure S2: DENSpm treatment increases stem cell potency without major effects on proliferation or apoptosis.** (**A**) Ratio of α6+/CD34+ cells after two weeks of 3D-3C culture with or without DFMO treatment for the last 5 days. mean±s.e.m. (n=4). (**B**) Ratio of α6+/CD34+ cells on day 0 and day 7 post-sorting starting from 100 % α6+/CD34+ cells (left; n=4) or 100 % α6+/CD34- cells (right; n=5) with or without DFMO treatment for the last 5 days of culture. mean±s.e.m. (**C**) Live cell number of α6+/CD34- cells (left; n=5) and α6+/CD34+cells (right; n=3) on day 7 post-sorting. mean±s.d. (**D**) Representative images of organoid cultures on day 7 post-sorting. (**C**,**D**) Treatment was performed for the last five days of culture. (**E**) Ratio of α6+/CD34+ cells after two weeks of 3D-3C culture with or without DENSpm treatment for the last 5 days. mean±s.e.m. (n=4). (**F**) Schematic representation of the workflow for colony formation assay after 3D-3C organoid culture. (**G**) Representative image of tissue culture plate after colony formation assay using cells with or without DENSpm treatment in 3D-3C organoids (n=2). (**H**) Quantification of colony number in (**G**). mean±s.d. (n=3; technical duplicates). (**I**) Ratio of EdU+ cells with or without DENSpm treatment for the last 72 h. EdU was incorporated for 2 h. mean±s.e.m. (n=4). (**J**) Ratio of annexin V+ cells with or without DENSpm treatment for the last 24 h. mean±s.e.m. (n=4). (**K**) Live cell number of α6+/CD34- cells (left; n=5) and α6+/CD34+cells (right; n=4) on day 7 post-sorting. mean±s.d. (**L**) Representative images of organoid cultures on day 7 post-sorting. (**K**,**L**) Treatment was performed for the last five days of culture. (**A**,**C**,**E**) Statistical significance was calculated by paired *t*-test. two-tailed p-values: * $p<0.05$; ns: not significant. (**B**,**H**,**I**,**J**) Statistical significance was calculated by unpaired *t*-test. two-tailed p-values: *** $p<0.001$; ns: not significant. (**K**) Statistical significance was calculated by one-way ANOVA Dunnett post-test. p-values: *** $p<0.001$, ns: not significant. (**A**,**B**,**C**,**E**,**G**,**I**,**J**,**K**) The cells were isolated from n mice. Each dot represents one biological replicate (n). (**H**) Each dot represents one technical replicate. (**A**,**B**,**I**,**J**) Analysis was performed in technical duplicates.

Journal of Cell Science • Supplementary information

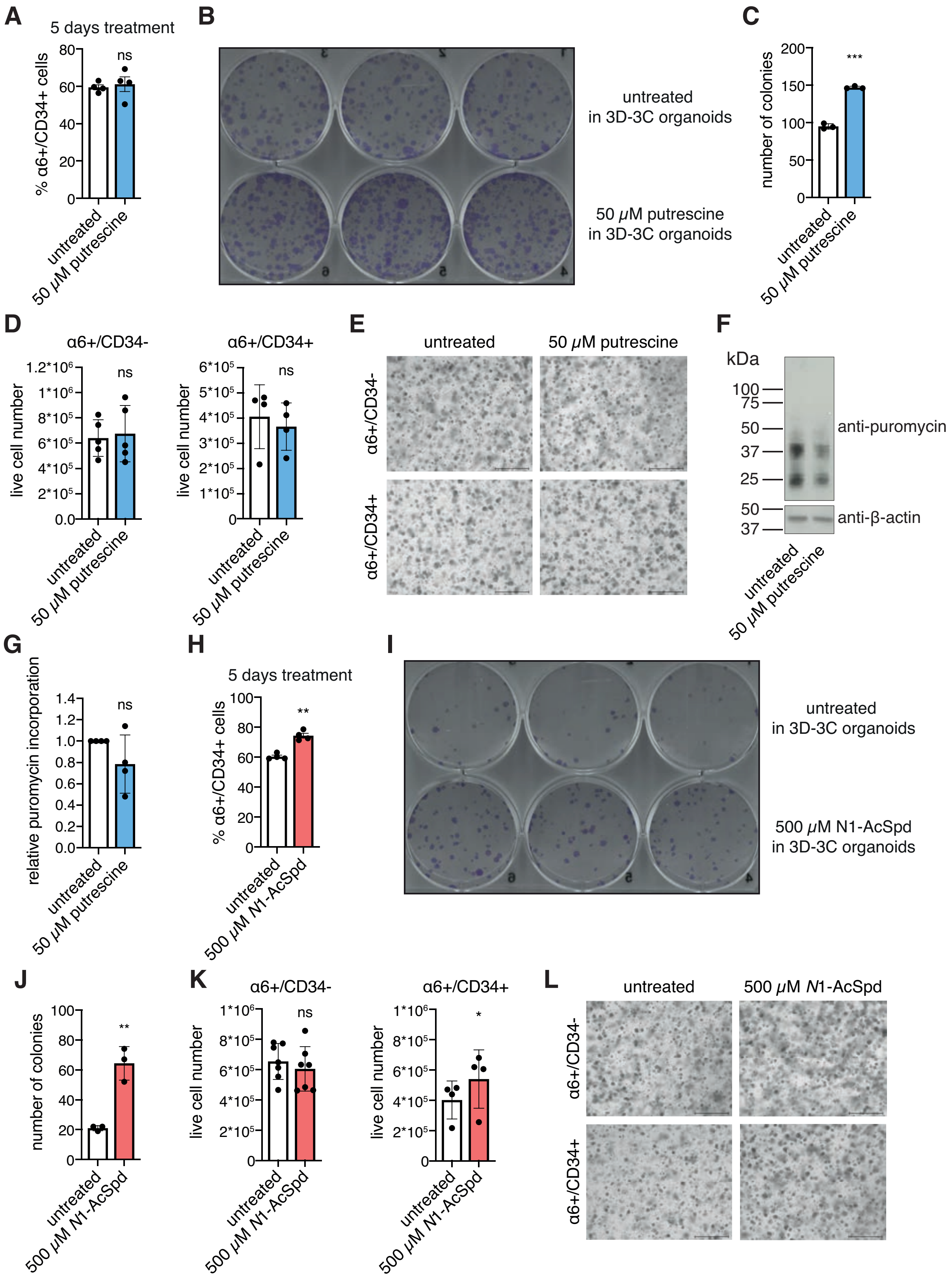


Journal of Cell Science • Supplementary information

**Figure S3: Putrescine supplementation and *N*1-acetylspermidine treatment increase stem cell potency.** (**A**) Ratio of α6+/CD34+ cells after two weeks of 3D-3C culture with or without putrescine treatment for the last five days. mean±s.e.m. (n=4). (**B**) Representative image of tissue culture plate after colony formation assay using cells with or without putrescine treatment in 3D-3C organoids (n=2). (**C**) Quantification of colony number of plate in (B). mean±s.d. (n=3; technical replicates). (**D**) Live cell number of α6+/CD34- cells (left; n=5) and α6+/CD34+cells (right; n=4) on day 7 post-sorting. mean±s.d. (**E**) Representative images of organoid cultures on day 7 post-sorting. (**D**,**E**) Treatment was performed for the last five days of culture. (**F**) Representative Western blot analysis after puromycin incorporation in 3D-3C cultured cells with or without putrescine treatment for the last 72 h of culture. (**G**) Quantification of Western blot analysis as shown in (**F**). mean±s.d. (n=4). (**H**) Ratio of α6+/CD34+ cells after two weeks of 3D-3C culture with or without *N*1-AcSpd treatment for the last five days. mean±s.e.m. (n=4). (**I**) Representative image of tissue culture plate after colony formation assay using cells with or without *N*1-AcSpd treatment in 3D-3C organoids (n=3). (**J)** Quantification of colony number of plate in (**I**). mean±s.d. (n=3; technical replicates). (**K**) Live cell number of α6+/CD34- cells (left; n=7) and α6+/CD34+cells (right; n=4) on day 7 post-sorting. mean±s.d. (**L**) Representative images of organoid cultures on day 7 post-sorting. (**K**,**L**) Treatment was performed for the last five days of culture. (**A**,**D**,**G**,**H**,**K**) Statistical significance was calculated by paired *t*-test. two-tailed p-values: ** p<0.01, * p<0.05, ns: not significant. (**C**,**J**) Statistical significance was calculated by unpaired *t*-test. two-tailed p-values: *** p<0.0001, ** p<0.01. (**A**,**D**,**G**,**H**,**K**) The cells were isolated from n mice. Each dot represents one biological replicate (n). (**C**,**J**) Each dot represent one technical replicate. (**A**,**H**) Analysis was performed in technical duplicates.

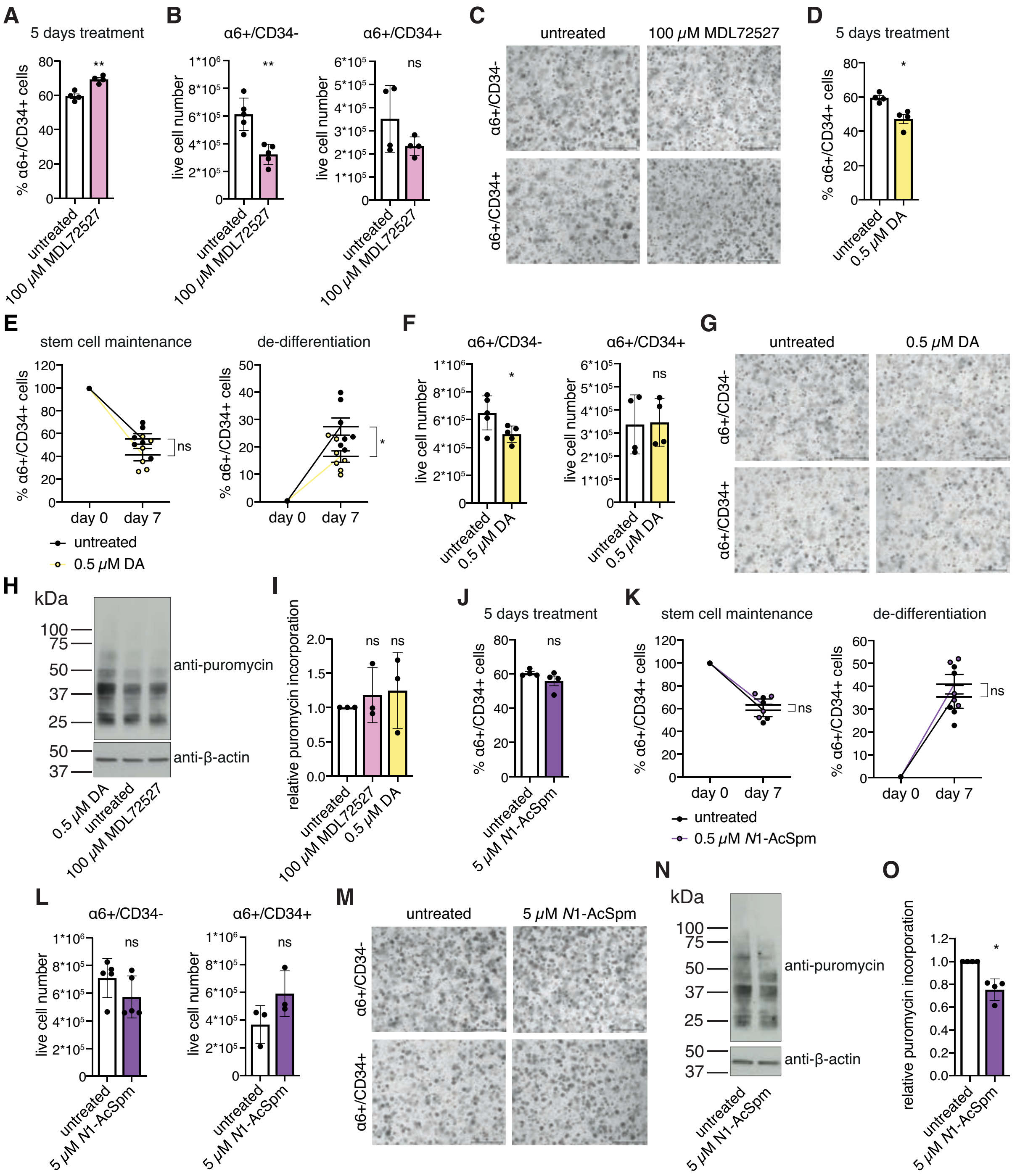


Journal of Cell Science • Supplementary information

**Figure S4: Inhibition of PAOX and SSAT have opposing effects, while *N*1-acetylspermine treatment does not affect hair follicle stem cell fate in the 3D-3C organoids.** (**A**) Ratio of α6+/CD34+ cells after two weeks of 3D-3C culture with or without MDL72527 treatment for the last five days. mean±s.e.m. (n=4). (**B**) Live cell number of α6+/CD34- cells (left; n=5) and α6+/CD34+cells (right; n=4) on day 7 post-sorting. mean±s.d. (**C**) Representative images of organoid cultures on day 7 post-sorting. (**D**) Ratio of α6+/CD34+ cells after two weeks of 3D-3C culture with or without diminazene aceturate (DA) treatment for the last five days. mean ±s.e.m. (n=4). (**E**) Ratio of α6+/CD34+ cells at day 0 and day 7 post-sorting starting from 100 % α6+/CD34+ cells (left; n=6) or 100 % α6+/CD34- cells (right; n=7) with or without DA treatment. mean±s.e.m. (**F**) Live cell number of α6+/CD34- cells (left; n=5) and α6+/CD34+cells (right; n=4) on day 7 post-sorting. mean±s.d. (**G**) Representative images of organoid cultures on day 7 post-sorting. (**H**) Representative Western blot analysis after puromycin incorporation in 3D-3C cultured cells with or without MDL72527 or DA treatment for the last 72 h of culture. (**I**) Quantification of Western blot analysis as shown in (**H**). mean±s.d. (n=3). (**J**) Ratio of α6+/CD34+ cells after two weeks of 3D-3C culture with or without *N*1-AcSpm treatment for the last five days. mean±s.e.m. (n=4). (**K**) Ratio of α6+/CD34+ cells at day 0 and day 7 post-sorting starting from 100 % α6+/CD34+ cells (left; n=4) or 100 % α6+/CD34- cells (right; n=5) with or without *N*1-AcSpm treatment. mean±s.e.m. (**L**) Live cell number of α6+/CD34- cells (left; n=5) and α6+/CD34+cells (right; n=3) on day 7 post-sorting. mean±s.d. (**M**) Representative images of organoid cultures on day 7 post-sorting. (**N**) Representative Western blot analysis after puromycin incorporation in 3D-3C cultured cells with or without *N*1-AcSpm treatment for the last 72 h of culture. (**O**) Quantification of Western blot analysis as shown in (**N**). mean±s.d. (n=4). (**A**,**B**,**D**,**F**,**J**,**L**,**O**) Statistical significance was calculated by paired *t*-test. two-tailed p-values: ** p<0.01, * p<0.05, ns: not significant. (**E**,**K**) Statistical significance was calculated by unpaired *t*-test. two-tailed p-values: * p<0.05, ns: not significant. (**I**) Statistical significance was calculated by one-way ANOVA Dunnett's post-test. ns: not significant. (**A**,**B**,**D**-**F**,**I**-**L**,**O**) Each dot represents one biological replicate (n). (**A**,**D**,**E**,**J**,**K**) Analysis was performed in technical duplicates. (**B**,**C**,**E**-**G**,**K**-**M**) Treatment was performed for the last five days of culture.

Journal of Cell Science • Supplementary information

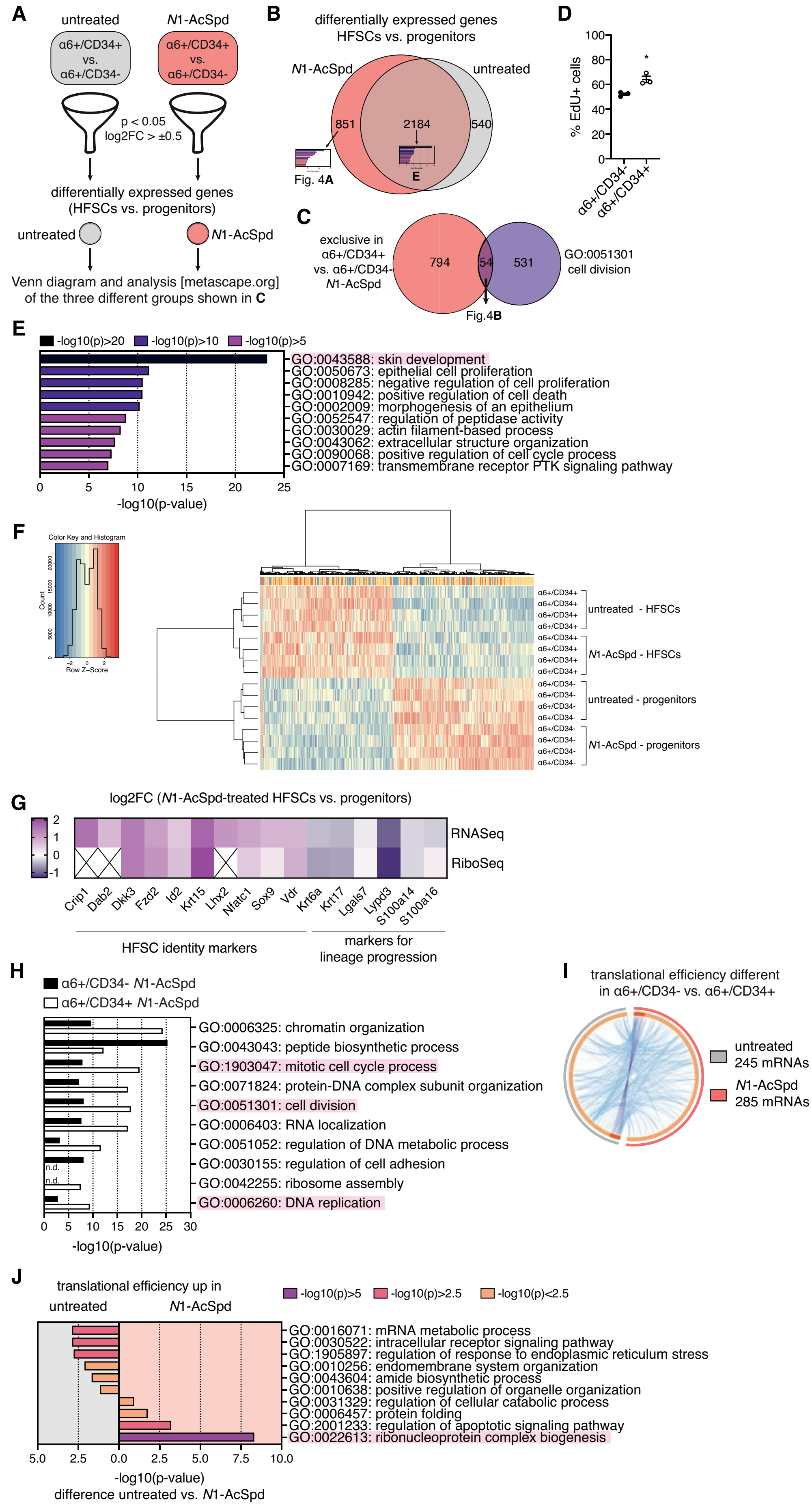


Journal of Cell Science • Supplementary information

**Figure S5: 3' RNA-sequencing and Ribo-sequencing analysis upon *N*1-acetylspermidine treatment.** (**A**) Schematic representation of the bioinformatic workflow. α6+/CD34- cells and α6+/CD34+ cells were compared for untreated and treated conditions. Genes were filtered (p-value < 0.05, log2FC > ±0.5) and the resulting lists were used for further analysis. untreated is shown in gray, *N*1-AcSpd is depicted in red. (**B**) Venn diagram of the two groups from (**A**). untreated is shown in gray, *N*1-AcSpd is depicted in red. (**C**) Venn diagram of differentially expressed genes upon *N*1-AcSpd treatment (red) and genes covered by GO term cell division (purple). (**D**) Ratio of EdU+ cells in α6+/CD34- progenitors and α6+/CD34+ HFSCs. mean±s.e.m. (n=3). Statistical significance was calculated by paired *t*-test. two-tailed p-value: * p<0.05. Each dot represents one biological replicate (n). Analysis was performed in technical duplicates. (**E**) GO term analysis of differentially expressed genes between cell types (p-value < 0.05, log2FC > ±0.5) from 3' RNASeq experiment common in untreated and treated cells (overlap shown in (**B**), biological process, metascape.org). (**F**) Heat map showing differentially expressed genes with a p≤0.05 (α6+/CD34- vs. α6+/CD34+; treated or untreated). (**G**) Heatmap showing mean values of log2FC of different HFSC identity markers (left) and markers for lineage progression (right) comparing RNASeq (top) and RiboSeq (bottom) upon *N*1-AcSpd treatment. The ratio of the log2FC in HFSCs vs. progenitor cells is displayed. A crossed cell indicates that the respective transcript was not detected in at least one sample in the RiboSeq experiment. (n=2 for each condition). (**H**) GO term analysis of all translated genes comparing α6+/CD34- progenitor cells and α6+/CD34+ HFSCs upon *N*1-AcSpd treatment (biological process, metascape.org). The ten GO terms with the biggest difference in enrichment between the translatomes of progenitors and HFSCs are shown. (**I**) Circos plot showing the overlap at the transcript level (purple lines) and the GO term level (blue lines) between differentially translated transcripts (p<0.05) in α6+/CD34- progenitors vs. α6+/CD34+ HFSCs in untreated compared to *N*1-AcSpd-treated cells. (**J**) GO term analysis of differentially translated transcripts (p<0.05) in α6+/CD34- progenitors vs. α6+/CD34+ HFSCs in untreated compared to *N*1-AcSpd-treated cells. The ten GO terms with the biggest difference between untreated and *N*1-AcSpd-treated cells are shown. GO terms enriched in untreated cells are shown on the left, GO terms enriched in *N*1-AcSpd-treated cells are display on the right side.

## Supplementary references

**Chacón-Martínez, C. A., Klose, M., Niemann, C., Glauche, I. & Wickström, S. A.** (2017). Hair follicle stem cell cultures reveal self-organizing plasticity of stem cells and their progeny. *EMBO J.* **36,** 151-164.
