# Supplementary material for: N1-acetylspermidine is a determinant of hair follicle stem cell fate
Source: J Cell Sci. 2021 May 11;134(9):jcs252767. doi: 10.1242/jcs.252767 (PMC8182411; doi:10.1242/jcs.252767)
Supplement: Reviewer comments [file joces-134-252767_review_history.pdf]

Journal of Cell Science

Review COMMONS

*N*1-acetylspermidine is a determinant of hair follicle stem cell fate

Kira Allmeroth, Christine S. Kim, Andrea Annibal, Andromachi Pouikli, Janis Koester, Maxime J. Derisbourg, Carlos Andrés Chacón-Martınez, Christian Latza, Adam Antebi, Peter Tessarz, Sara A. Wickström and Martin S. Denzel

DOI: 10.1242/jcs.252767

Editor: Andrew Ewald

Review timeline

Submission to Review Commons:	4 May 2020
Submission to Journal of Cell Science:	5 August 2020
Editorial decision:	18 August 2020
First revision received:	12 January 2021
Editorial decision:	11 March 2021
Second revision received:	16 March 2021
Accepted:	20 March 2021

Reviewer 1

Evidence, reproducibility and clarity

The authors utilize an in vitro cell culture system to examine cells sorted from the skin via CD34+/Sca1-/a6+ as hair follicle stem cells and CD34-/Sca1-/a6+ as potential progenitor cells from the outer root sheath. Using biochemical assays in cell culture they determine that the presumed stem cell population is increased when translation is inhibited and is also affected by manipulating the polyamine content of the cells. They find that these manipulation affect the cell cycle progression of the presumed stem cells and their clonal ability.

The study is generally well executed, with the caveat of a pure in vitro model system, and well written with sufficient clarity for the non-specialist reader. The main concern is that the cell culture system clearly perturbs the cell division patterns of hair follicle stem cells, which are normally quiescent and rampantly dividing like we see them in vivo. The changes that hair follicle stem cells undergo in cell culture are substantial and the assays and markers used here are insufficient to examine cell fate decisions without validation in vivo.

Significance

While I find the idea of the study and the approach interesting, the study is quite minimal and preliminary. It suffers from chronic lack of in vivo data from the live tissue, and lack of genetic approaches that would allow solid conclusions to bring significant advancement in the field. The conclusions are diffuse and do not seem to go beyond what we largely seems to know of stem cells in general and the applicability to hair follicle in particular is questionable due to the lack of in vivo data.

Reviewer 2

Evidence, reproducibility and clarity

Summary:

In this manuscript, Allmeroth et al. investigate whether the increase in mRNA translation associated with stem cell activation and differentiation is regulated via polyamines. To this end, the authors

© 2021. Published by The Company of Biologists under the terms of the Creative Commons Attribution License (https://creativecommons.org/licenses/by/4.0/).

use the well-defined stem cell model of the Hair Follicle in the skin. Most of the work is performed by implementing an in vitro organoid culture system (3D-3C) developed by Chacon-martinez et.al (2017) that allows for the long-term culture of Hair Follicle Stem Cells (HFSCs) and their direct progeny.

The authors start by validating the levels of translation in the CD34+/- basal cell population of in vivo hair follicles, and in agreement with the literature they found reduced translation rates in stem compared to progenitor cells. This observation was reproducible when using 3D-3C in vitro cultures, justifying the use of this in vitro system for the study.

To test the role of polyamines in both stem cell maintenance and cell fate decision, the authors compare the effect of a translation inhibitor (G418) with that of a DENSpm, a spermine analogue which depletes natural polyamines resulting in decreased mRNA translation. The authors conclude that decreased translation promotes the stem cell state. Interestingly, when depleting all polyamines upon DFMO treatment, it did not have a significant impact on the cell fate. This made authors hypothesize that DENSpm treatment may be exerting its effect through a mechanism independent from mRNA translation. This led to the realization that the increased levels of N1-acteylspermidine (N1-AcSpd) in response to DENSpm or putrescine treatment may be the cause of the tilt towards stem cell fate (a6+/CD34+) in a translation independent fashion. The authors conclude that an acceleration in cell cycle progression is the mechanism by which N1-AcSpd shifts the balance towards the stem cell state in their HFSC organotypic cultures, and that this happens in a translation independent fashion.

Comments:

In this study the authors claim that polyamines mediate HFSC maintenance and function through different mechanisms. One of the mechanisms explored is the role of polyamines as regulators of mRNA translation, for which a precedent has already been established. Based on previous observations revealing that an increase in translation is associated with HFSC activation and differentiation, the authors aim at identifying whether polyamines mediate given increase of translation. However, despite this representing a major aim of the study translation is not assessed routinely to check the impact of each treatment. For instance, the authors compare the observations made upon inhibition of translation (G418 treatment), to those upon DENSpm treatment without further confirming whether this treatment actually impacts on translation. Similarly, despite the strong conclusions that they draw from the DFMO experiment, suggesting that reduced mRNA translation through decreased polyamine was not the only determinant of the cell fate changes observed upon DENSpm treatment, the authors never confirm whether this treatment impacted or not on translation. And, in fact, they do not do it either for putrescine. Other than the initial experiment where they test the inhibitor of translation G418, they do not perform a puromycin incorporation assay up until looking into the effect of N1-AcSpd, the proposed intermediate promoting the proliferative effect of DENSpm treatment. In my opinion, these conclusions need to be reinforced with further experimental evidence to validate the connections made throughout the study.

Some additional experiments should be performed to confirm the strong conclusions drawn by the authors. Similarly, a more detailed account of the experimental conditions used for each and every dataset would help clarify the observations made.

1- The dose and duration of the treatments should be clearly specified in the methods section. The authors should indicate whether treatment of 3C-3D cultures was initiated before or after they had reached equilibrium in the ratio of CD34+ and CD34- cells, which according to Chacon-Martinez et al (2017) occurs around day 10-14.
In general, the method section should be more detailed. Dose, duration and details of the treatments are often missing. The validations performed to determine the experimental conditions used need to be included to assess specificity of the treatment.

2- Additionally, most of the results shown for G418 corresponded to the last 4h of culture. However, when analysing the effect on sorted pure populations of either CD34- or CD34+ cells, the authors state they were cultured for one week with or without treatment. It is critical to specify the experimental conditions and end points for all datasets. For example, were G418, DENSpm,

© 2021. Published by The Company of Biologists under the terms of the Creative Commons Attribution License (https://creativecommons.org/licenses/by/4.0/).

Putrescine, or N1-AcSpd present in the cultures during the 7 days?; were the treatments added several times? Likewise, the authors should justify the criteria used to determine the doses used for polyamine supplementation. For example, 500µm N1-AcSpd and 5µm N1-AcSpd in Figure 3; are these based in physiological concentrations, literature, or previous titration experiments.

3- Targeting an essential cellular process as translation must undoubtedly have several implications in cell survival, signalling and adhesion. It has been shown that DENSpm treatment induces cell detachment and apoptosis in glioblastoma cells (Tian et al. 2012). It would be informative to asses if the size or morphology of the organoids changed after the different treatments. Similarly, any changes in cell attachment and cell shedding could impact on the apoptosis quantification (performed via Annexin V staining). It would be important to ensure that this quantification has not missed any apoptotic detached cells from organoids. Authors should additionally check that cell death did not occur throughout the 7/14 days of treatment, especially if repeated doses were added.

4- When looking into the CD34+ cells, the data is presented in the form of percentages after 7 days of culture. It would be critical to show the final total number of cells obtained after each treatment. This would help to understand the impact of the treatment, how comparable is to control conditions and any significant change in the culture conditions that could significantly impact the results.

5- Previous work has shown that Putrescine impacts epidermal cells proliferation (Takigawa et al. 1977). In the experiments shown here N1-AcSpd increases putrescine to levels even higher than those used in the Putrescine treatment. The authors should discuss why they associate the changes in cell cycle progression only to N1-AcSpd, and clarify how they can discern the specific effects of N1-AcSpd from those of Putrescine.

6- Given that the authors centre the study around the potential polyamine regulation of translation and its impact on cell fate, a much more thorough characterization of puromycin incorporation and translation should be made throughout the study.

7- Given the association made by the authors between polyamine treatment and HFSC fate, it would be ideal to have information about how cells behave upon several passages. One would want to know whether the bias in cell fate is something acquired by the cells upon treatment, or whether it can be reversed. Similarly, it would be interesting to know whether the treatment affects long-term stem cell exhaustion. This data would be of translational relevance if this treatment was to be used to increase the regenerative capacity of epithelial cells.

8- This study identifies N1-acetylspermidine as a novel regulator of cell fate decisions using a 3D culture system. However, additional data supporting as to whether this treatment impacts on the response of skin cells in vivo is missing. Treatment of the skin upon wounding (via topical/local application) would represent an ideal model were to explore this further.

9- Figure 4 B is too packed and gene names are difficult to read unless the image is significantly magnified.

References

Chacon-Martinez CA, Klose M, Niemann C, Glauche I, Wickstrom SA (2017) Hair follicle stem cell cultures reveal self-organizing plasticity of stem cells and their progeny. EMBO J 36: 151-164

Takigawa M, Inoue H, Gohda E, Asada A, Takeda Y, Mori Y. The role of putrescine in cell proliferation of the skin of mice induced by ethylphenylpropriolate. Exp Mol Pathol. 1977;27(2):183-196. doi:10.1016/0014-4800(77)90029-6

Tian Y, Wang S, Wang B, Zhang J, Jiang R, Zhang W. Overexpression of SSAT by DENSPM treatment induces cell detachment and apoptosis in glioblastoma. Oncol Rep. 2012;27(4):1227-1232. doi:10.3892/or.2011.1592

© 2021. Published by The Company of Biologists under the terms of the Creative Commons Attribution License (https://creativecommons.org/licenses/by/4.0/).

Significance

Understanding how single-cell behaviour is coordinated at the population level, and the specific mechanisms driving changes in epithelial stem cell fate will provide valuable knowledge on how complex tissues are maintained and regenerated. Previous studies have shown that low translation functionally contributes to maintain stem cells (Blanco 2016; Kim 2019; Pegg 2016; Roux 2018; Tahmasebi 2019). This manuscript reinforces an already reported finding using an in vitro organoid system to grow HFSCs.

In this manuscript, Allmeroth et. al. present N1-acetylspermidine as a potential regulator of stem cell proliferation and stemness, providing new insights that might be potentially valuable in regenerative medicine. This study, however, does not go further than a mere characterization of the observation using a 3D culture system. Additional data revealing whether this treatment impacts on the response of skin to tissue challenges, such as the wounding, would enrich the significance of the present work.

References

Blanco S, Bandiera R, Popis M, et al. Stem cell function and stress response are controlled by protein synthesis. Nature. 2016;534(7607):335-340. doi:10.1038/nature18282

Dever TE, Ivanov IP. Roles of polyamines in translation. J Biol Chem. 2018;293(48):18719- 18729. doi:10.1074/jbc.TM118.003338

Kim HJ. Cell Fate Control by Translation: mRNA Translation Initiation as a Therapeutic

Target for Cancer Development and Stem Cell Fate Control. Biomolecules. 2019;9(11):665. Published 2019 Oct 29. doi:10.3390/biom9110665

Medema, J., Vermeulen, L. Microenvironmental regulation of stem cells in intestinal homeostasis and cancer. Nature 474, 318-326 (2011).

Pegg AE. Functions of Polyamines in Mammals. J Biol Chem. 2016;291(29):14904-14912. doi:10.1074/jbc.R116.731661

Roux PP, Topisirovic I. Signaling Pathways Involved in the Regulation of mRNA Translation. Mol Cell Biol. 2018;38(12):e00070-18. Published 2018 May 29. doi:10.1128/MCB.00070-18

Saygin C, Matei D, Majeti R, Reizes O, Lathia JD. Targeting Cancer Stemness in the Clinic: From Hype to Hope. Cell Stem Cell. 2019;24(1):25-40. doi:10.1016/j.stem.2018.11.017

Tahmasebi S, Amiri M, Sonenberg N. Translational Control in Stem Cells. Front Genet. 2019;9:709. Published 2019 Jan 15. doi:10.3389/fgene.2018.00709

Reviewer 3

Evidence, reproducibility and clarity

In their manuscript Allmeroth and colleagues describe novel roles for polyamines in regulating stem cell fate. Using hair follicle stem cell organoids, the authors find that specific depletion of polyamines enhanced stem cell function by lowering translation rates. In contrast, inhibition of N1-acetylspermidine regulated cell proliferation without decreasing mRNA translation. Thus, this study linked polyamine metabolism to distinct aspects of stem cell fate.

It is well known that polyamines contribute to efficiency and fidelity of protein synthesis within cells, yet their role in regulating stem cell fate remains largely unknown. Here, the authors dissect the roles of polyamines in regulating protein synthesis and cell proliferation during hair follicle differentiation. Several recent studies demonstrated that the tight regulation of protein synthesis is

© 2021. Published by The Company of Biologists under the terms of the Creative Commons Attribution License (https://creativecommons.org/licenses/by/4.0/).

required for proper stem cell function in blood, skin and brain for example. However, the precise molecular mechanisms how de novo protein synthesis rate is controlled in stem and differentiated cells remained largely unclear. While the authors confirm roles of polyamines during stem cell differentiation in organoids, the data remain purely descriptive and do not inform about the underlying mechanisms. Moreover, the authors do not provide direct evidence that it is indeed repression of translation that actively promotes the stem cell state of CD34+/ITGA6+ stem cells in their organoid culture. The regulatory roles of spermidine and spermine in mRNA translaion are known, yet the authors do not provide any evidence that similar mechanisms apply to their study.

Specific comments:

1.Page 7; lines 136-138: "It has been previously described that stem cells display lower translation rates than their differentiated counterparts (Tahmasebi et al., 2018a). To test if this was also true in epidermal stem cells, we sorted freshly isolated mouse epidermal cells using three different markers..." The authors should acknowledge that this has been shown before in vivo (Blanco et al., 2016). The concept that tissue stem cells display lower translation rates than their differentiated counterparts has been demonstrated for at least blood, brain and the epidermis (Baser et al., 2019; Blanco et al., 2016; Signer et al., 2014). Yet, this may not be the case for all tissues and the author should differentiate the studies better by citing the original studies.

2.The authors use a previously established hair follicle stem cell organoid culture system for their further analyses. While the protocols are established and the behaviour of the hair follicle stem cells as organoids have been defined (Chacon-Martinez et al., 2017), the authors should provide evidence that the organoids behave similar in their hands by at least showing some examples and by providing the raw data from the flow sorting experiment.

3.Moreover, the authors make the statement: "Based on their transcriptome and marker expression analysis, these progenitor cells represent HF outer root sheath (ORS) cells and inner bulge cells (Kim et al., 2019),.." The citation refers to a reference submitted to publication. This is not acceptable and the authors must remove unpublished citations and provide this evidence by experiments or remove these claims from the manuscript.

4.The authors perform RNA-seq experiments to confirm that CD34+/ITGA6- cells are ORS progenitors. However, the data provided do not confirm identity. The markers solely provide evidence that CD34+/ITGA+ cells are less differentiated than CD34-/ITGA6+ cells.

5. Figure 1G: the authors do not provide convincing evidence that translation elongation is reduced upon G418 application, the results shown in Figure S1D are not significant.

6. Figure 1H is misleading as the populations are not 100% or 0%. The authors do not provide evidence that it is indeed differentiation driving the differences as opposed to survival for instance.

7. Figure 2: The authors do not provide evidence that DENSpm-treated cells indeed reduce global translation rates.

8. The data shown in Figure S2C do not cohere. CD34+/ITGA+ seem more proliferative than CD34-/ITGA+ cells, yet administration of DENSpm does not increase the number of EdU+ cells when all live cells are considered. However, the authors argument is that DENSpm enriches the number of CD34+/ITGA+. A similar argument applies for Figure S2D. It also worrying that the overall survival rate of CD34+/ITGA6+ cells is generally higher indicating that survival might be a driver for the results. For instance, cell stress reduces global translation and CD34+/ITGA6+ cells may simply cope better with stress.

9. Page 9: The authors state: "Taken together, these data suggest that reduced translation is not merely a consequence of stemness. Instead, decreasing translation actively promotes the stem cell state." While this has been shown before in several adult tissues in vivo including skin, it is an overstatement regarding the authors own data because no evidence is provided that the treatment indeed resulted in either global or more specific mRNA translation.

© 2021. Published by The Company of Biologists under the terms of the Creative Commons Attribution License (https://creativecommons.org/licenses/by/4.0/).

10. In addition to directly acting on the translation apparatus to stimulate protein synthesis, polyamine spermidine has been shown to regulate specific translation due to its essential role for eukaryotic translation factor 5A (eIF5A). Multiple studies have also linked lower levels of polyamines with increased misreading during translation elongation. The authors need to provide evidence for a change in global and/or specific protein translation, for instance by performing polysome profiling or ribosome footprinting.

11. It has already been demonstrated that over-expression of spermidine/spermine N1-acetyltransferase 1 (SAT1) in mammalian cells was sufficient to suppress protein synthesis via spermidine and spermine (Mandal et al., 2013). The authors should acknowledge these findings.

12. Similar to my comments above, the finding that N1-acetylspermidine enhances cell proliferation without reducing translation is potentially interesting but unlikely to be independent of a role in translation. The finding that cell cycle regulators are up-regulated do not point to any underlying mechanism.

Baser, A., Skabkin, M., Kleber, S., Dang, Y., Gulculer Balta, G.S., Kalamakis, G., Gopferich, M., Ibanez, D.C., Schefzik, R., Lopez, A.S., et al. (2019). Onset of differentiation is post-transcriptionally controlled in adult neural stem cells. Nature 566, 100-104.

Blanco, S., Bandiera, R., Popis, M., Hussain, S., Lombard, P., Aleksic, J., Sajini, A., Tanna, H., Cortes-Garrido, R., Gkatza, N., et al. (2016). Stem cell function and stress response are controlled by protein synthesis. Nature 534, 335-340.

Chacon-Martinez, C.A., Klose, M., Niemann, C., Glauche, I., and Wickstrom, S.A. (2017). Hair follicle stem cell cultures reveal self-organizing plasticity of stem cells and their progeny. The EMBO journal 36, 151-164.

Mandal, S., Mandal, A., Johansson, H.E., Orjalo, A.V., and Park, M.H. (2013). Depletion of cellular polyamines, spermidine and spermine, causes a total arrest in translation and growth in mammalian cells. Proc Natl Acad Sci U S A 110, 2169-2174.

Signer, R.A., Magee, J.A., Salic, A., and Morrison, S.J. (2014). Haematopoietic stem cells require a highly regulated protein synthesis rate. Nature 509, 49-54.

Significance

Several recent studies demonstrated that tissue stem cells often reduce de novo protein synthesis, and that this repression of translation is required for stem cell functions.
Polyamines are known to regulate global and specific mRNA translation through several mechanisms but their function in regulating stem cell fate has not been studied yet.

Author response to reviewers' comments

Reviewer #1:
The authors utilize an in vitro cell culture system to examine cells sorted from the skin via CD34+/Sca1-/a6+ as hair follicle stem cells and CD34-/Sca1-/a6+ as potential progenitor cells from the outer root sheath. Using biochemical assays in cell culture they determine that the presumed stem cell population is increased when translation is inhibited and is also affected by manipulating the polyamine content of the cells. They find that these manipulations affect the cell cycle progression of the presumed stem cells and their clonal ability.

The study is generally well executed, with the caveat of a pure in vitro model system, and well written with sufficient clarity for the non-specialist reader. The main concern is that the cell culture system

© 2021. Published by The Company of Biologists under the terms of the Creative Commons Attribution License (https://creativecommons.org/licenses/by/4.0/).

clearly perturbs the cell division patterns of hair follicle stem cells, which are normally quiescent and rampantly dividing like we see them in vivo. The changes that hair follicle stem cells undergo in cell culture are substantial and the assays and markers used here are insufficient to examine cell fate decisions without validation in vivo.

Reviewer #1 (Significance (Required)):
While I find the idea of the study and the approach interesting, the study is quite minimal and preliminary. It suffers from chronic lack of in vivo data from the live tissue, and lack of genetic approaches that would allow solid conclusions to bring significant advancement in the field. The conclusions are diffuse and do not seem to go beyond what we largely seem to know of stem cells in general and the applicability to hair follicle in particular is questionable due to the lack of in vivo data.

We appreciate the reviewer's summary of our study and agree that in vivo experiments would test the relevance of our findings from the culture system. The 3C organoid system for epidermal stem cells has been validated extensively and it is known that the cells maintain their identity. Consistently, they are able to repopulate and regenerate the appropriate niche, as demonstrated by hair growth after cell transplantation to nude mice (Chacon-Martinez et al., 2017). Nonetheless, we agree that proliferation conditions are of course different between the hair bulge niche and the organoid system.
To address the reviewer's comments, we will perform in vivo experiments in mice. After careful consideration we decided to perform depilation experiments. Here, the hair is removed, a treatment that forces regeneration and, if done repeatedly, depletes the stem cell population. We will perform depilation experiments and simultaneously treat mice with DENSpm, putrescine, N-Acetyl-Spermidine, and N-Acetyl-Spermine and empty vehicle controls. Treatments will be done topically after depilation. In a first cohort, we will collect tissue 48 hours after depilation and analyze proliferation markers to detect stem cell activation. In another cohort, we will serially depilate to test if the treatments can delay stem cell exhaustion. We already have the ethics permission for these experiments and are eager to initiate this work soon.

Reviewer #2:

Summary:
In this manuscript, Allmeroth et al. investigate whether the increase in mRNA translation associated with stem cell activation and differentiation is regulated via polyamines. To this end, the authors use the well-defined stem cell model of the Hair Follicle in the skin. Most of the work is performed by implementing an in vitro organoid culture system (3D-3C) developed by Chacon-Martinez et al. (2017) that allows for the long-term culture of Hair Follicle Stem Cells (HFSCs) and their direct progeny.

The authors start by validating the levels of translation in the CD34+/- basal cell population of in vivo hair follicles, and in agreement with the literature they found reduced translation rates in stem compared to progenitor cells. This observation was reproducible when using 3D-3C in vitro cultures, justifying the use of this in vitro system for the study.

To test the role of polyamines in both stem cell maintenance and cell fate decision, the authors compare the effect of a translation inhibitor (G418) with that of a DENSpm, a spermine analogue which depletes natural polyamines resulting in decreased mRNA translation. The authors conclude that decreased translation promotes the stem cell state. Interestingly, when depleting all polyamines upon DFMO treatment, it did not have a significant impact on the cell fate. This made authors hypothesize that DENSpm treatment may be exerting its effect through a mechanism independent from mRNA translation. This led to the realization that the increased levels of N1-acteylspermidine (N1-AcSpd) in response to DENSpm or putrescine treatment may be the cause of the tilt towards stem cell fate (a6+/CD34+) in a translation independent fashion. The authors conclude that an acceleration in cell cycle progression is the mechanism by which N1-AcSpd shifts the balance towards the stem cell state in their HFSC organotypic cultures, and that this happens in a translation independent fashion.

Comments:
In this study the authors claim that polyamines mediate HFSC maintenance and function through different mechanisms. One of the mechanisms explored is the role of polyamines as regulators of mRNA translation, for which a precedent has already been established. Based on previous observations

© 2021. Published by The Company of Biologists under the terms of the Creative Commons Attribution License (https://creativecommons.org/licenses/by/4.0/).

revealing that an increase in translation is associated with HFSC activation and differentiation, the authors aim at identifying whether polyamines mediate given increase of translation. However, despite this representing a major aim of the study translation is not assessed routinely to check the impact of each treatment. For instance, the authors compare the observations made upon inhibition of translation (G418 treatment), to those upon DENSpm treatment without further confirming whether this treatment actually impacts on translation. Similarly, despite the strong conclusions that they draw from the DFMO experiment, suggesting that reduced mRNA translation through decreased polyamine was not the only determinant of the cell fate changes observed upon DENSpm treatment, the authors never confirm whether this treatment impacted or not on translation. And, in fact, they do not do it either for putrescine. Other than the initial experiment where they test the inhibitor of translation G418, they do not perform a puromycin incorporation assay up until looking into the effect of N1-AcSpd, the proposed intermediate promoting the proliferative effect of DENSpm treatment. In my opinion, these conclusions need to be reinforced with further experimental evidence to validate the connections made throughout the study.

We thank the reviewer for the careful analysis of our work. We agree that monitoring mRNA translation rates at each step of the manuscript's experimental progression is essential before drawing conclusions. We have initiated these experiments and are working to overcome some experimental difficulties with the approach. Specifically, we will quantify puromycin incorporation after G418, DENSpm, DMFO, and putrescine treatments.

Some additional experiments should be performed to confirm the strong conclusions drawn by the authors. Similarly, a more detailed account of the experimental conditions used for each and every dataset would help clarify the observations made.

We will make sure to expand the methods section and to delineate all experimental conditions for the various data sets.

1- The dose and duration of the treatments should be clearly specified in the methods section. The authors should indicate whether treatment of 3C-3D cultures was initiated before or after they had reached equilibrium in the ratio of CD34+ and CD34- cells, which according to Chacon-Martinez et al (2017) occurs around day 10-14.
In general, the method section should be more detailed. Dose, duration and details of the treatments are often missing. The validations performed to determine the experimental conditions used need to be included to assess specificity of the treatment.

The manuscript text will be modified to include all of this important information.

2- Additionally, most of the results shown for G418 corresponded to the last 4h of culture. However, when analysing the effect on sorted pure populations of either CD34- or CD34+ cells, the authors state they were cultured for one week with or without treatment. It is critical to specify the experimental conditions and end points for all datasets. For example, were G418, DENSpm, Putrescine, or N1-AcSpd present in the cultures during the 7 days?; were the treatments added several times? Likewise, the authors should justify the criteria used to determine the doses used for polyamine supplementation. For example, 500μm N1-AcSpd and 5μm N1-AcSpd in Figure 3; are these based in physiological concentrations, literature, or previous titration experiments.

We will modify the paper to include all of these points. The selection of concentrations was based on titration experiments that we will refer to in the updated version of the manuscript.

3- Targeting an essential cellular process as translation must undoubtedly have several implications in cell survival, signaling and adhesion. It has been shown that DENSpm treatment induces cell detachment and apoptosis in glioblastoma cells (Tian et al. 2012). It would be informative to asses if the size or morphology of the organoids changed after the different treatments. Similarly, any changes in cell attachment and cell shedding could impact on the apoptosis quantification (performed via Annexin V staining). It would be important to ensure that this quantification has not missed any apoptotic detached cells from organoids. Authors should additionally check that cell death did not occur throughout the 7/14 days of treatment, especially if repeated doses were added.

We will provide data regarding cell size and morphology from our FACS data. We will ensure that our

© 2021. Published by The Company of Biologists under the terms of the Creative Commons Attribution License (https://creativecommons.org/licenses/by/4.0/).

results are not influenced by differences in cell survival and provide live cell numbers for the different treatments.

4- When looking into the CD34+ cells, the data is presented in the form of percentages after 7 days of culture. It would be critical to show the final total number of cells obtained after each treatment. This would help to understand the impact of the treatment, how comparable is to control conditions and any significant change in the culture conditions that could significantly impact the results.

The requested information will be included in new supplementary material.

5- Previous work has shown that Putrescine impacts epidermal cells proliferation (Takigawa et al. 1977). In the experiments shown here N1-AcSpd increases putrescine to levels even higher than those used in the Putrescine treatment. The authors should discuss why they associate the changes in cell cycle progression only to N1-AcSpd, and clarify how they can discern the specific effects of N1-AcSpd from those of Putrescine.

This is an important conceptual point that we will address in further detail. In short, we assign the observed effects to N1-AcSpd because additional putrescine supplementation in DENSpm treated cultures only partially rescued stem cell maintenance and de-differentiation. The only common change upon these treatments is the elevation of N1-AcSpd. Still, we are aware that we cannot exclude a contribution of putrescine to the effects observed upon N1-AcSpd, based on the current data. Therefore, we will analyze cell cycle progression upon putrescine supplementation. Further, we will include topical application of putrescine in our in vivo experiments.

6- Given that the authors center the study around the potential polyamine regulation of translation and its impact on cell fate, a much more thorough characterization of puromycin incorporation and translation should be made throughout the study.

As pointed out above, we will carefully track translation rates with the various treatments.

7- Given the association made by the authors between polyamine treatment and HFSC fate, it would be ideal to have information about how cells behave upon several passages. One would want to know whether the bias in cell fate is something acquired by the cells upon treatment, or whether it can be reversed. Similarly, it would be interesting to know whether the treatment affects long-term stem cell exhaustion. This data would be of translational relevance if this treatment was to be used to increase the regenerative capacity of epithelial cells.

We highly appreciate this suggestion and will perform additional experiments to address this point. We will perform long-term organoid cultures to test if the cell fate changes are reversible and if the cultured stem cells become depleted with time. We are excited about this project as it will inform the in vivo depilation experiments we are planning.

8- This study identifies N1-acetylspermidine as a novel regulator of cell fate decisions using a 3D culture system. However, additional data supporting as to whether this treatment impacts on the response of skin cells in vivo is missing. Treatment of the skin upon wounding (via topical/local application) would represent an ideal model were to explore this further.

As detailed above, we have decided to use serial depilation as the in vivo paradigm to test the relevance of our in vitro work. In contrast to wound healing, depilation experiments will allow us to investigate behavior of the cells in their natural niche environment. Further, it enables us to not only investigate HFSC activation, but also ORS cell de-differentiation.

9- Figure 4 B is too packed and gene names are difficult to read unless the image is significantly magnified.

Figure 4 will be update to make it more reader-friendly.

References

Chacon-Martinez CA, Klose M, Niemann C, Glauche I, Wickstrom SA (2017) Hair follicle stem cell

© 2021. Published by The Company of Biologists under the terms of the Creative Commons Attribution License (https://creativecommons.org/licenses/by/4.0/).

cultures reveal self-organizing plasticity of stem cells and their progeny. EMBO J 36: 151-164
Takigawa M, Inoue H, Gohda E, Asada A, Takeda Y, Mori Y. The role of putrescine in cell proliferation of the skin of mice induced by ethylphenylpropriolate. Exp Mol Pathol. 1977;27(2):183-196. doi:10.1016/0014-4800(77)90029- 6
Tian Y, Wang S, Wang B, Zhang J, Jiang R, Zhang W. Overexpression of SSAT by DENSPM treatment induces cell detachment and apoptosis in glioblastoma. Oncol Rep. 2012;27(4):1227-1232. doi:10.3892/or.2011.1592

Reviewer #2 (Significance):
Understanding how single-cell behaviour is coordinated at the population level, and the specific mechanisms driving changes in epithelial stem cell fate will provide valuable knowledge on how complex tissues are maintained and regenerated. Previous studies have shown that low translation functionally contributes to maintain stem cells (Blanco 2016; Kim 2019; Pegg 2016; Roux 2018; Tahmasebi 2019). This manuscript reinforces an already reported finding using an in vitro organoid system to grow HFSCs.
In this manuscript, Allmeroth et. al. present N1-acetylspermidine as a potential regulator of stem cell proliferation and stemness, providing new insights that might be potentially valuable in regenerative medicine. This study, however, does not go further than a mere characterization of the observation using a 3D culture system. Additional data revealing whether this treatment impacts on the response of skin to tissue challenges, such as the wounding, would enrich the significance of the present work.

We agree with the reviewer's assessment that reduced translation has been associated with stem cell maintenance. Going beyond the current state of the art, we show, first, that the 3D- 3C organoid system can faithfully recapitulate this in vivo phenomenon. Second, we demonstrate a role of polyamines in stemness via mRNA translation. Finally, we extend this understanding by demonstrating a new role of N1-acetylspermidine in stem cell maintenance that goes beyond regulation of mRNA translation. Using further experiments in mice we now seek to demonstrate the in vivo relevance of these findings. With this, the manuscript will go well beyond reinforcing a previous observation but will instead move the needle when it comes to the understanding of metabolic regulation of stem cell states.

References
Blanco S, Bandiera R, Popis M, et al. Stem cell function and stress response are controlled by protein synthesis. Nature. 2016;534(7607):335-340. doi:10.1038/nature18282
Dever TE, Ivanov IP. Roles of polyamines in translation. J Biol Chem. 2018;293(48):18719-18729. doi:10.1074/jbc.TM118.003338
Kim HJ. Cell Fate Control by Translation: mRNA Translation Initiation as a Therapeutic Target for Cancer Development and Stem Cell Fate Control. Biomolecules. 2019;9(11):665. Published 2019 Oct 29. doi:10.3390/biom9110665
Medema, J., Vermeulen, L. Microenvironmental regulation of stem cells in intestinal homeostasis and cancer. Nature 474, 318-326 (2011).
Pegg AE. Functions of Polyamines in Mammals. J Biol Chem. 2016;291(29):14904-14912. doi:10.1074/jbc.R116.731661
Roux PP, Topisirovic I. Signaling Pathways Involved in the Regulation of mRNA Translation. Mol Cell Biol. 2018;38(12):e00070-18. Published 2018 May 29. doi:10.1128/MCB.00070-18
Saygin C, Matei D, Majeti R, Reizes O, Lathia JD. Targeting Cancer Stemness in the Clinic: From Hype to Hope. Cell Stem Cell. 2019;24(1):25-40. doi:10.1016/j.stem.2018.11.017
Tahmasebi S, Amiri M, Sonenberg N. Translational Control in Stem Cells. Front Genet. 2019;9:709. Published 2019 Jan 15. doi:10.3389/fgene.2018.00709

Reviewer #3

In their manuscript Allmeroth and colleagues describe novel roles for polyamines in regulating stem cell fate. Using hair follicle stem cell organoids, the authors find that specific depletion of polyamines enhanced stem cell function by lowering translation rates. In contrast, inhibition of N1-acetylspermidine regulated cell proliferation without decreasing mRNA translation. Thus, this study linked polyamine metabolism to distinct aspects of stem cell fate.

© 2021. Published by The Company of Biologists under the terms of the Creative Commons Attribution License (https://creativecommons.org/licenses/by/4.0/).

It is well known that polyamines contribute to efficiency and fidelity of protein synthesis within cells, yet their role in regulating stem cell fate remains largely unknown. Here, the authors dissect the roles of polyamines in regulating protein synthesis and cell proliferation during hair follicle differentiation. Several recent studies demonstrated that the tight regulation of protein synthesis is required for proper stem cell function in blood, skin and brain for example. **However, the precise molecular mechanisms how de novo protein synthesis rate is controlled in stem and differentiated cells remained largely unclear.** While the authors confirm roles of polyamines during stem cell differentiation in organoids, the data remain purely descriptive and do not inform about the underlying mechanisms. Moreover, the authors do not provide direct evidence that it is indeed repression of translation that actively promotes the stem cell state of CD34+/ITGA6+ stem cells in their organoid culture. The regulatory roles of spermidine and spermine in mRNA translation are known, yet the authors do not provide any evidence that similar mechanisms apply to their study.

To address the reviewer's key comment, we will carefully analyze mRNA translation in the organoid cultures. We will analyze translation rates across all treatments in the manuscript. More importantly, we will perform ribosome foot printing to mechanistically understand how de novo protein synthesis affects cell identity. We disagree with the reviewer on the point that our paper lacks the evidence for a direct role of repressed translation to promote the stem cell state. To address this point, we have pharmacologically inhibited translation and find this to be sufficient for an increased stem cell ratio. Further, the reviewer's summary leaves out the important finding of the paper regarding the role of N1-acetylspermidine, which we find to affect cell identity through a mechanism that does not involve altered mRNA translation rates. This new and more differentiated view of polyamine mediated control of stem cell identity is an important element of the paper.

Specific comments:

1. Page 7; lines 136-138: "It has been previously described that stem cells display lower translation rates than their differentiated counterparts (Tahmasebi et al., 2018a). To test if this was also true in epidermal stem cells, we sorted freshly isolated mouse epidermal cells using three different markers..." The authors should acknowledge that this has been shown before in vivo (Blanco et al., 2016). The concept that tissue stem cells display lower translation rates than their differentiated counterparts has been demonstrated for at least blood, brain and the epidermis (Baser et al., 2019; Blanco et al., 2016; Signer et al., 2014). Yet, this may not be the case for all tissues and the author should differentiate the studies better by citing the original studies.

The respective modifications and references will be introduced into the manuscript to better reflect the current state of the literature.

2. The authors use a previously established hair follicle stem cell organoid culture system for their further analyses. While the protocols are established and the behaviour of the hair follicle stem cells as organoids have been defined (Chacon-Martinez et al., 2017), the authors should provide evidence that the organoids behave similar in their hands by at least showing some examples and by providing the raw data from the flow sorting experiment.

We have closely collaborated with the team of Prof. Sara Wickström, who is also a co-author of the study, to make sure that this complex tissue culture system works well in our hands, and we are confident that it does. We will provide additional data to support this.

3. Moreover, the authors make the statement: "Based on their transcriptome and marker expression analysis, these progenitor cells represent HF outer root sheath (ORS) cells and inner bulge cells (Kim et al., 2019),.." The citation refers to a reference submitted to publication. This is not acceptable and the authors must remove unpublished citations and provide this evidence by experiments or remove these claims from the manuscript.

We agree with the reviewer and will only cite this paper once it is published.

4. The authors perform RNA-seq experiments to confirm that CD34+/ITGA6- cells are ORS progenitors. However, the data provided do not confirm identity. The markers solely provide evidence that CD34+/ITGA+ cells are less differentiated than CD34-/ITGA6+ cells.

© 2021. Published by The Company of Biologists under the terms of the Creative Commons Attribution License (https://creativecommons.org/licenses/by/4.0/).

We will re-word out conclusions to be more conservative in our interpretation of the data.

5. Figure 1G: the authors do not provide convincing evidence that translation elongation is reduced upon G418 application, the results shown in Figure S1D are not significant.

As pointed out above, mRNA translation rates will be re-evaluated and the claim will only be made if this can be supported with significant data.

6. Figure 1H is misleading as the populations are not 100% or 0%. The authors do not provide evidence that it is indeed differentiation driving the differences as opposed to survival for instance.
To make sure that the populationsare pure, we use FACS sorting before initiating the culture. Thus, we can certain as to the population at the starting point. We will provide additional data to assess whether better survival or higher frequency of cell division are potential confounding factors.

7. Figure 2: The authors do not provide evidence that DENSpm-treated cells indeed reduce global translation rates.

We have first experimental evidence to support this point and will add it to the revised version of the manuscript. As pointed out above, mRNA translation rates will be evaluated with all treatments used in this study.

8. The data shown in Figure S2C do not cohere. CD34+/ITGA+ seem more proliferative than CD34-/ITGA+ cells, yet administration of DENSpm does not increase the number of EdU+ cells when all live cells are considered. However, the authors argument is that DENSpm enriches the number of CD34+/ITGA+. A similar argument applies for Figure S2D. It also worrying that the overall survival rate of CD34+/ITGA6+ cells is generally higher indicating that survival might be a driver for the results. For instance, cell stress reduces global translation and CD34+/ITGA6+ cells may simply cope better with stress.

We agree with the reviewer that an increase in the number of ▫6+/CD34+ cells should reflect on higher proliferation of all live cells upon DENSpm. Probably, the long incubation period (24h) prohibits to detect these differences. We will adjust the incubation time for EdU to 2h, as shown for N1-AcSpd in Fig. 4, and re-assess proliferation. For Fig. S2D all live cells do show a trend towards reduced apoptosis, according to the increase in ▫6+/CD34+ cells. However, DENSpm rather increases apoptosis in both cell population. Thus, we would not expect a different result here.
We agree with the reviewer that ▫6+/CD34+ cells show lower rates of apoptosis and at the same time higher proliferation rates. However, this also applies to untreated cultures. The self- organizing plasticity of the cultures results in a 50:50 balance, suggesting that this advantage is compensated. The balance is influenced by self-renewal and differentiation of stem cells, as well as proliferation and de-differentiation of progenitor cells. Our results presented in Figure S2 clearly demonstrate, that DENSpm treatment did not affect cell proliferation or apoptosis, thereby showing that our results are not due to the fact that stem cells cope better with stress.

9. Page 9: The authors state: "Taken together, these data suggest that reduced translation is not merely a consequence of stemness. Instead, decreasing translation actively promotes the stem cell state." While this has been shown before in several adult tissues in vivo including skin, it is an overstatement regarding the authors own data because no evidence is provided that the treatment indeed resulted in either global or more specific mRNA translation.

We will provide additional experimental results and will only make this claim if it is supported by solid evidence.

10. In addition to directly acting on the translation apparatus to stimulate protein synthesis, polyamine spermidine has been shown to regulate specific translation due to its essential role for eukaryotic translation factor 5A (eIF5A). Multiple studies have also linked lower levels of polyamines with increased misreading during translation elongation. The authors need to provide evidence for a change in global and/or specific protein translation, for instance by performing polysome profiling or ribosome footprinting.

We will use ribosome foot printing to characterize translation in the cell populations present in the

© 2021. Published by The Company of Biologists under the terms of the Creative Commons Attribution License (https://creativecommons.org/licenses/by/4.0/).

culture. This will add relevant additional data and will bring the field forward as the role of specific mRNA translation for stem cell identity remains unclear at this point.

11. It has already been demonstrated that over-expression of spermidine/spermine N1-acetyltransferase 1 (SAT1) in mammalian cells was sufficient to suppress protein synthesis via spermidine and spermine (Mandal et al., 2013). The authors should acknowledge these findings.

We are aware of this work and will make sure to cite it appropriately.

12. Similar to my comments above, the finding that N1-acetylspermidine enhances cell proliferation without reducing translation is potentially interesting but unlikely to be independent of a role in translation. The finding that cell cycle regulators are up-regulated do not point to any underlying mechanism.

We will look at the effects of N1-acetylspermidine not only regarding the transcriptome but will also test if there is an effect on specific mRNA translation via ribosome foot printing. In turn we will also look at cell cycle regulators in the DENSpm treated cells to see if there are overlapping effects.

References:
Baser, A., Skabkin, M., Kleber, S., Dang, Y., Gulculer Balta, G.S., Kalamakis, G., Gopferich, M., Ibanez, D.C., Schefzik, R., Lopez, A.S., et al. (2019). Onset of differentiation is post-transcriptionally controlled in adult neural stem cells. Nature 566, 100-104.
Blanco, S., Bandiera, R., Popis, M., Hussain, S., Lombard, P., Aleksic, J., Sajini, A., Tanna, H., Cortes-Garrido, R., Gkatza, N., et al. (2016). Stem cell function and stress response are controlled by protein synthesis. Nature 534, 335-340.
Chacon-Martinez, C.A., Klose, M., Niemann, C., Glauche, I., and Wickstrom, S.A. (2017). Hair follicle stem cell cultures reveal self-organizing plasticity of stem cells and their progeny. The EMBO journal 36, 151-164.
Mandal, S., Mandal, A., Johansson, H.E., Orjalo, A.V., and Park, M.H. (2013). Depletion of cellular polyamines, spermidine and spermine, causes a total arrest in translation and growth in mammalian cells. Proc Natl Acad Sci U S A 110, 2169-2174.
Signer, R.A., Magee, J.A., Salic, A., and Morrison, S.J. (2014). Haematopoietic stem cells require a highly regulated protein synthesis rate. Nature 509, 49-54.

Significance:
Several recent studies demonstrated that tissue stem cells often reduce de novo protein synthesis, and that this repression of translation is required for stem cell functions. Polyamines are known to regulate global and specific mRNA translation through several mechanisms but their function in regulating stem cell fate has not been studied yet.

We want to highlight the reviewer's perspective on the significance of our work. By adding additional data, most importantly in vivo experiments as well as ribosome foot printing, we will be able to better support the claims we make in the paper. We aim to provide a link between polyamine metabolism and specific mRNA translation in the control fate of adult stem cells.

First decision letter

MS ID#: JOCES/2020/252767

MS TITLE: Polyamine-controlled proliferation and protein biosynthesis are independent determinants of hair follicle stem cell fate

© 2021. Published by The Company of Biologists under the terms of the Creative Commons Attribution License (https://creativecommons.org/licenses/by/4.0/).

AUTHORS: Kira Allmeroth, Christine S Kim, Andrea Annibal, Andromachi Pouikli, Carlos Andres Chacon-Martinez, Christian Latza, Adam Antebi, Peter Tessarz, Sara A Wickstrom, and Martin Sebastian Denzel
ARTICLE TYPE: Research Article

We have now reached a decision on the above manuscript.

I have now reviewed the transferred reviews and your proposed revision plan. I consider the manuscript to be interesting and well within the scope of JCS. You are proposing to respond comprehensively to all reviewer concerns and I read their reviews as representing significant interest in a revised manuscript. I therefore invite revisions and will promptly return a revised manuscript to these same reviewers. Please let me know if you need more time for experimental revisions or if any other issues arise where I can provide clarity.

We are aware that you may currently be unable to access the lab to undertake experimental revisions. If it would be helpful, we encourage you to contact us to discuss your revision in greater detail. Please send us a point-by-point response indicating where you are able to address concerns raised (either experimentally or by changes to the text) and where you will not be able to do so within the normal timeframe of a revision. We will then provide further guidance. Please also note that we are happy to extend revision timeframes as necessary.

Please ensure that you clearly highlight all changes made in the revised manuscript. Please avoid using 'Tracked changes' in Word files as these are lost in PDF conversion.

I should be grateful if you would also provide a point-by-point response detailing how you have dealt with the points raised by the reviewers in the 'Response to Reviewers' box. Please attend to all of the reviewers' comments. If you do not agree with any of their criticisms or suggestions please explain clearly why this is so.

First revision

Author response to reviewers' comments

Dear Dr. Ewald,
We are happy to resubmit our manuscript to the Journal of Cell Science with major revisions. Please find below a point-by-point response to the reviewers' comments.
Best regards,
Martin Denzel

Reviewer #1:
The authors utilize an in vitro cell culture system to examine cells sorted from the skin via CD34+/Sca1-/a6+ as hair follicle stem cells and CD34-/Sca1-/a6+ as potential progenitor cells from the outer root sheath. Using biochemical assays in cell culture they determine that the presumed stem cell population is increased when translation is inhibited and is also affected by manipulating the polyamine content of the cells. They find that these manipulations affect the cell cycle progression of the presumed stem cells and their clonal ability.
The study is generally well executed, with the caveat of a pure in vitro model system, and well written with sufficient clarity for the non-specialist reader. The main concern is that the cell culture system clearly perturbs the cell division patterns of hair follicle stem cells, which are normally quiescent and rampantly dividing like we see them in vivo. The changes that hair follicle stem cells undergo in cell culture are substantial and the assays and markers used here are insufficient to examine cell fate decisions without validation in vivo.

© 2021. Published by The Company of Biologists under the terms of the Creative Commons Attribution License (https://creativecommons.org/licenses/by/4.0/).

Reviewer #1 (Significance (Required)):
While I find the idea of the study and the approach interesting, the study is quite minimal and preliminary. It suffers from chronic lack of in vivo data from the live tissue, and lack of genetic approaches that would allow solid conclusions to bring significant advancement in the field. The conclusions are diffuse and do not seem to go beyond what we largely seem to know of stem cells in general and the applicability to hair follicle in particular is questionable due to the lack of in vivo data.

We appreciate the reviewer's summary of our study. We have now added substantial data to our paper that address the reviewer's concerns.
1- We have further validated the 3C organoid system and show that it is an appropriate model for the analysis of mRNA translation in epidermal stem cells. Previously, the 3C organoid system for epidermal stem cells had been validated extensively and it is known that the cells maintain their identity. Consistently, they are able to repopulate and regenerate the appropriate niche, as demonstrated by hair growth after cell transplantation to nude mice (Chacon- Martinez et al., 2017). We have now added additional RNA sequencing data confirming that, likewise, in our hands, the 3C system faithfully maintains the delicate balance of stem and precursor cells. We further concur with the reviewer that the lack of in vivo data left some of our data unsubstantiated for their role outside the organoidsystem.
2- To address this point, we have performed in vivo experiments in mice. After careful consideration we decided to perform depilation experiments. Here, the hair is removed along with the inner bulge layer, a treatment that triggers regeneration. We found that this synchronized regeneration goes along with a specific elevation of N1-acetylspermidine, consistent with our findings from the organoid culture.
3- To gain deeper insights into the mechanisms of translational control in stem cell maintenance, and to go beyond what was already understood from similar work, we have performed a ribosome sequencing experiment on sorted cells and find important cell-type specific differences.
4- Based on new data we were able to sharpen our conclusions and find that while reduced translation is a feature of epidermal stem cells, a forced reduction is not always sufficient for stem cell maintenance. Instead, polyamines emerge as a key regulator of stemness, affecting both translation and proliferation.

Reviewer #2:
In this manuscript, Allmeroth et al. investigate whether the increase in mRNA translation associated with stem cell activation and differentiation is regulated via polyamines. To this end, the authors use the well-defined stem cell model of the Hair Follicle in the skin. Most of the work is performed by implementing an in vitro organoid culture system (3D-3C) developed by Chacon-Martinez et al. (2017) that allows for the long-term culture of Hair Follicle Stem Cells (HFSCs) and their direct progeny.
The authors start by validating the levels of translation in the CD34+/- basal cell population of in vivo hair follicles, and in agreement with the literature they found reduced translation rates in stem compared to progenitor cells. This observation was reproducible when using 3D-3C in vitro cultures, justifying the use of this in vitro system for the study.
To test the role of polyamines in both stem cell maintenance and cell fate decision, the authors compare the effect of a translation inhibitor (G418) with that of a DENSpm, a spermine analogue which depletes natural polyamines resulting in decreased mRNA translation. The authors conclude that decreased translation promotes the stem cell state. Interestingly, when depleting all polyamines upon DFMO treatment, it did not have a significant impact on the cell fate. This made authors hypothesize that DENSpm treatment may be exerting its effect through a mechanism independent from mRNA translation. This led to the realization that the increased levels of N1-acteylspermidine (N1-AcSpd) in response to DENSpm or putrescine treatment may be the cause of the tilt towards stem cell fate (a6+/CD34+) in a translation independent fashion. The authors conclude that an acceleration in cell cycle progression is the mechanism by which N1-AcSpd shifts the balance towards the stem cell state in their HFSC organotypic cultures, and that this happens in a translation independent fashion.

In this study the authors claim that polyamines mediate HFSC maintenance and function through different mechanisms. One of the mechanisms explored is the role of polyamines as regulators of mRNA translation, for which a precedent has already been established. Based on previous observations revealing that an increase in translation is associated with HFSC activation and

© 2021. Published by The Company of Biologists under the terms of the Creative Commons Attribution License (https://creativecommons.org/licenses/by/4.0/).

differentiation, the authors aim at identifying whether polyamines mediate given increase of translation. However, despite this representing a major aim of the study translation is not assessed routinely to check the impact of each treatment. For instance, the authors compare the observations made upon inhibition of translation (G418 treatment), to those upon DENSpm treatment without further confirming whether this treatment actually impacts on translation. Similarly, despite the strong conclusions that they draw from the DFMO experiment, suggesting that reduced mRNA translation through decreased polyamine was not the only determinant of the cell fate changes observed upon DENSpm treatment, the authors never confirm whether this treatment impacted or not on translation. And, in fact, they do not do it either for putrescine. Other than the initial experiment where they test the inhibitor of translation G418, they do not perform a puromycin incorporation assay up until looking into the effect of N1-AcSpd, the proposed intermediate promoting the proliferative effect of DENSpm treatment. In my opinion, these conclusions need to be reinforced with further experimental evidence to validate the connections made throughout the study.

We thank the reviewer for the careful analysis of our work. We agree that monitoring mRNA translation rates at each step of the manuscript's experimental progression is essential before drawing conclusions. We have now included the puromycin incorporation assay at all stages of the manuscript. This has indeed led us to alter the conclusions of the paper as we did not observe a reduction in translation upon DENSpm treatment. This made our findings much more interesting and pointed to a finely balanced role of the various polyamines that we now characterize in depth.

Some additional experiments should be performed to confirm the strong conclusions drawn by the authors. Similarly, a more detailed account of the experimental conditions used for each and every dataset would help clarify the observations made.

We have expanded the methods section and delineated all experimental conditions for the various data sets.

1. The dose and duration of the treatments should be clearly specified in the methods section. The authors should indicate whether treatment of 3C-3D cultures was initiated before or after they had reached equilibrium in the ratio of CD34+ and CD34- cells, which according to Chacon-Martinez et al (2017) occurs around day 10-14.
In general, the method section should be more detailed. Dose, duration and details of the treatments are often missing. The validations performed to determine the experimental conditions used need to be included to assess specificity of the treatment.

The manuscript has been modified to include this important information.

2. Additionally, most of the results shown for G418 corresponded to the last 4h of culture. However, when analysing the effect on sorted pure populations of either CD34- or CD34+ cells, the authors state they were cultured for one week with or without treatment. It is critical to specify the experimental conditions and end points for all datasets. For example, were G418, DENSpm, Putrescine, or N1-AcSpd present in the cultures during the 7 days?; were the treatments added several times? Likewise, the authors should justify the criteria used to determine the doses used for polyamine supplementation. For example, 500μm N1-AcSpd and 5μm N1-AcSpd in Figure 3; are these based in physiological concentrations, literature, or previous titration experiments.

We have modified the paper to include all of these points. The selection of concentrations was based on titration experiments that we are referring to in the updated version of the manuscript.

3. Targeting an essential cellular process as translation must undoubtedly have several implications in cell survival, signaling and adhesion. It has been shown that DENSpm treatment induces cell detachment and apoptosis in glioblastoma cells (Tian et al. 2012). It would be informative to asses if the size or morphology of the organoids changed after the different treatments. Similarly, any changes in cell attachment and cell shedding could impact on the apoptosis quantification (performed via Annexin V staining). It would be important to ensure that this quantification has not missed any apoptotic detached cells from organoids. Authors should additionally check that cell death did not occur throughout the 7/14 days of treatment, especially if repeated doses were added.

© 2021. Published by The Company of Biologists under the terms of the Creative Commons Attribution License (https://creativecommons.org/licenses/by/4.0/).

In the new supplementary data, we provide images of the organoid cultures. We also repeated the analysis of apoptosis including detached cells in the supernatant. We performed this analysis 24h after treatment to monitor acute consequences on apoptosis.

4. When looking into the CD34+ cells, the data is presented in the form of percentages after 7 days of culture. It would be critical to show the final total number of cells obtained after each treatment. This would help to understand the impact of the treatment, how comparable is to control conditions and any significant change in the culture conditions that could significantly impact the results.

The requested information is now included. While some treatments significantly affect cell viability, our main conclusions are not affected by these differences.

5. Previous work has shown that Putrescine impacts epidermal cells proliferation (Takigawa et al. 1977). In the experiments shown here N1-AcSpd increases putrescine to levels even higher than those used in the Putrescine treatment. The authors should discuss why they associate the changes in cell cycle progression only to N1-AcSpd, and clarify how they can discern the specific effects of N1-AcSpd from those of Putrescine.

This is an important conceptual point that we have addressed in further detail. In short, we assigned the observed effects to N1-AcSpd because additional putrescine supplementation in DENSpm treated cultures only partially rescued stem cell maintenance and de-differentiation. The only common change upon these treatments is the elevation of N1-AcSpd. Still, the data of the previous version of the paper could not exclude a contribution of putrescine to the effects observed upon N1-AcSpd. Therefore, we have used a PAOX inhibitor, MDL72527, to prevent putrescine production from N1-acetylspermidine, a treatment that was per se sufficient to affect HFSC fate in the organoid culture (Figure 4). Together with the new data from the mouse depilation experiments we are confident that we observe a previously undiscovered role of N1- acetylspermidine.

6. Given that the authors center the study around the potential polyamine regulation of translation and its impact on cell fate, a much more thorough characterization of puromycin incorporation and translation should be made throughout the study.

We have now carefully tracked translation rates with the various treatments using puromycin incorporation, and have performed ribosome foot printing.

7. Given the association made by the authors between polyamine treatment and HFSC fate, it would be ideal to have information about how cells behave upon several passages. One would want to know whether the bias in cell fate is something acquired by the cells upon treatment, or whether it can be reversed. Similarly, it would be interesting to know whether the treatment affects long-term stem cell exhaustion. This data would be of translational relevance if this treatment was to be used to increase the regenerative capacity of epithelial cells.

We highly appreciate this suggestion and have performed additional experiments to address this point. We performed long-term organoid culture and repeatedly analyzed the HFSC ratio. We did not observe exhaustion of the stem cell population under continuous treatment. Withdrawal of the treatment did not reverse cell fate, suggesting a “memory” of the treatment. We included these data in the updated version of the manuscript.

8. This study identifies N1-acetylspermidine as a novel regulator of cell fate decisions using a 3D culture system. However, additional data supporting as to whether this treatment impacts on the response of skin cells in vivo is missing. Treatment of the skin upon wounding (via topical/local application) would represent an ideal model were to explore this further.

We thank the reviewer for the suggestion. To address this point, we have used depilation as the in vivo paradigm to test the relevance of our in vitro work. We observed a general reduction of polyamines with age in the mouse epidermis and observed a strong increase in N1- acetylspermidine levels upon forced regeneration. This was most pronounced in the young mice, correlating with faster hair regrowth.

9. Figure 4 B is too packed and gene names are difficult to read unless the image is significantly

© 2021. Published by The Company of Biologists under the terms of the Creative Commons Attribution License (https://creativecommons.org/licenses/by/4.0/).

magnified.

Figure 4 has been updated to make it more reader-friendly.

References
Chacon-Martinez CA, Klose M, Niemann C, Glauche I, Wickstrom SA (2017) Hair follicle stem cell cultures reveal self-organizing plasticity of stem cells and their progeny. EMBO J 36: 151-164
Takigawa M, Inoue H, Gohda E, Asada A, Takeda Y, Mori Y. The role of putrescine in cell proliferation of the skin of mice induced by ethylphenylpropriolate. Exp Mol Pathol. 1977;27(2):183-196. doi:10.1016/0014-4800(77)90029- 6
Tian Y, Wang S, Wang B, Zhang J, Jiang R, Zhang W. Overexpression of SSAT by DENSPM treatment induces cell detachment and apoptosis in glioblastoma. Oncol Rep. 2012;27(4):1227-1232. doi:10.3892/or.2011.1592

Reviewer #2 (Significance):
Understanding how single-cell behaviour is coordinated at the population level, and the specific mechanisms driving changes in epithelial stem cell fate will provide valuable knowledge on how complex tissues are maintained and regenerated. Previous studies have shown that low translation functionally contributes to maintain stem cells (Blanco 2016; Kim 2019; Pegg 2016; Roux 2018; Tahmasebi 2019). This manuscript reinforces an already reported finding using an in vitro organoid system to grow HFSCs.
In this manuscript, Allmeroth et. al. present N1-acetylspermidine as a potential regulator of stem cell proliferation and stemness, providing new insights that might be potentially valuable in regenerative medicine. This study, however, does not go further than a mere characterization of the observation using a 3D culture system. Additional data revealing whether this treatment impacts on the response of skin to tissue challenges, such as the wounding, would enrich the significance of the present work.

We agree with the reviewer's assessment that reduced translation has been associated with stem cell maintenance. Going beyond the current state of the art, we show, first, that the 3D- 3C organoid system faithfully recapitulates this in vivo phenomenon. Second, we demonstrate a role of polyamines in stemness. Finally, we extend this understanding by demonstrating a new role of N1-acetylspermidine in stem cell maintenance that goes beyond regulation of mRNA translation. Using further experiments in mice we now demonstrate the potential in vivo relevance of these findings, showing that N1-acetylspermidine is strongly induced during regeneration. With this, the new manuscript goes well beyond reinforcing a previous observation but is instead moving the needle further when it comes to the understanding of metabolic regulation of stem cell states.

References
Blanco S, Bandiera R, Popis M, et al. Stem cell function and stress response are controlled by protein synthesis. Nature. 2016;534(7607):335-340. doi:10.1038/nature18282
Dever TE, Ivanov IP. Roles of polyamines in translation. J Biol Chem. 2018;293(48):18719-18729. doi:10.1074/jbc.TM118.003338
Kim HJ. Cell Fate Control by Translation: mRNA Translation Initiation as a Therapeutic Target for Cancer Development and Stem Cell Fate Control. Biomolecules. 2019;9(11):665. Published 2019 Oct 29. doi:10.3390/biom9110665
Medema, J., Vermeulen, L. Microenvironmental regulation of stem cells in intestinal homeostasis and cancer. Nature 474, 318-326 (2011).
Pegg AE. Functions of Polyamines in Mammals. J Biol Chem. 2016;291(29):14904-14912. doi:10.1074/jbc.R116.731661
Roux PP, Topisirovic I. Signaling Pathways Involved in the Regulation of mRNA Translation. Mol Cell Biol. 2018;38(12):e00070-18. Published 2018 May 29. doi:10.1128/MCB.00070-18
Saygin C, Matei D, Majeti R, Reizes O, Lathia JD. Targeting Cancer Stemness in the Clinic: From Hype to Hope. Cell Stem Cell. 2019;24(1):25-40. doi:10.1016/j.stem.2018.11.017
Tahmasebi S, Amiri M, Sonenberg N. Translational Control in Stem Cells. Front Genet. 2019;9:709. Published 2019 Jan 15. doi:10.3389/fgene.2018.00709

© 2021. Published by The Company of Biologists under the terms of the Creative Commons Attribution License (https://creativecommons.org/licenses/by/4.0/).

Reviewer #3

In their manuscript Allmeroth and colleagues describe novel roles for polyamines in regulating stem cell fate. Using hair follicle stem cell organoids, the authors find that specific depletion of polyamines enhanced stem cell function by lowering translation rates. In contrast, inhibition of N1-acetylspermidine regulated cell proliferation without decreasing mRNA translation. Thus, this study linked polyamine metabolism to distinct aspects of stem cell fate.

It is well known that polyamines contribute to efficiency and fidelity of protein synthesis within cells, yet their role in regulating stem cell fate remains largely unknown. Here, the authors dissect the roles of polyamines in regulating protein synthesis and cell proliferation during hair follicle differentiation. Several recent studies demonstrated that the tight regulation of protein synthesis is required for proper stem cell function in blood, skin and brain for example. However, the precise molecular mechanisms how de novo protein synthesis rate is controlled in stem and differentiated cells remained largely unclear. While the authors confirm roles of polyamines during stem cell differentiation in organoids, the data remain purely descriptive and do not inform about the underlying mechanisms. Moreover, the authors do not provide direct evidence that it is indeed repression of translation that actively promotes the stem cell state of CD34+/ITGA6+ stem cells in their organoid culture. The regulatory roles of spermidine and spermine in mRNA translation are known, yet the authors do not provide any evidence that similar mechanisms apply to their study.

We thank the reviewer for this challenging summary of our work that has helped us to revise the paper with significant new data.
1. We have carefully delineated the role of polyamines in epidermal stem cells by tuning the pathway with additional inhibitor compounds and monitoring of the state of translation. Doing this, we now find that full depletion of polyamines, while inhibiting translation does not favor the HFSC ratio. This points to very specific roles of particular polyamines. In fact, the reviewer's summary leaves out the important finding of the paper regarding the role of N1- acetylspermidine, which we find to affect cell identity through a mechanism that does not involve reduced mRNA translation rates. This new and more differentiated view of polyamine mediated control of stem cell identity is an important element of the paper.
2. To gain a deeper insight into the state of mRNA translation, we have performed ribosome foot printing in stem and precursor cells providing an exciting new data set that shows that protein biosynthetic genes are regulated at the translational level.
3. We have performed in vivo analyses to test if our observations from the organoid system might translate to the intact organ and find that indeed N1-acetylspermidine is strongly induced when regeneration is triggered by depilation.
Together, the updated version of the paper provides new data that provide mechanistic insights into polyamine-regulated stem cell fate through translation and proliferation.

1. Page 7; lines 136-138: "It has been previously described that stem cells display lower translation rates than their differentiated counterparts (Tahmasebi et al., 2018a). To test if this was also true in epidermal stem cells, we sorted freshly isolated mouse epidermal cells using three different markers..." The authors should acknowledge that this has been shown before in vivo (Blanco et al., 2016). The concept that tissue stem cells display lower translation rates than their differentiated counterparts has been demonstrated for at least blood, brain and the epidermis (Baser et al., 2019; Blanco et al., 2016; Signer et al., 2014). Yet, this may not be the case for all tissues and the author should differentiate the studies better by citing the original studies.

The respective modifications and references have been introduced into the manuscript to better reflect the current state of the literature.

2. The authors use a previously established hair follicle stem cell organoid culture system for their further analyses. While the protocols are established and the behaviour of the hair follicle stem cells as organoids have been defined (Chacon-Martinez et al., 2017), the authors should provide evidence that the organoids behave similar in their hands by at least showing some examples and by providing the raw data from the flow sorting experiment.

© 2021. Published by The Company of Biologists under the terms of the Creative Commons Attribution License (https://creativecommons.org/licenses/by/4.0/).

We have closely collaborated with the team of Prof. Sara Wickström, who is also a co-author of the study, to make sure that this complex tissue culture system works well in our hands, and we are confident that it does. To further demonstrate this, we now provide comparisons between published and our own RNA seq data in Figure S1. Additionally, we included dot plots showing that the culture establishes the expected 50:50 ratio between cell types.

3. Moreover, the authors make the statement: "Based on their transcriptome and marker expression analysis, these progenitor cells represent HF outer root sheath (ORS) cells and inner bulge cells (Kim et al., 2019),.." The citation refers to a reference submitted to publication. This is not acceptable and the authors must remove unpublished citations and provide this evidence by experiments or remove these claims from the manuscript.

We agree with the reviewer and are now citing this published paper.

4. The authors perform RNA-seq experiments to confirm that CD34+/ITGA6- cells are ORS progenitors. However, the data provided do not confirm identity. The markers solely provide evidence that CD34+/ITGA+ cells are less differentiated than CD34-/ITGA6+ cells.

We have re-worded our conclusions.

5. Figure 1G: the authors do not provide convincing evidence that translation elongation is reduced upon G418 application, the results shown in Figure S1D are not significant.

To streamline the manuscript and given the unclear results with G418, we now focus on the polyamine pathway and its role in HFSC fate decisions and removed the G418 data from the manuscript.

6. Figure 1H is misleading as the populations are not 100% or 0%. The authors do not provide evidence that it is indeed differentiation driving the differences as opposed to survival for instance.

To make sure that the populations are pure, we used FACS sorting before initiating the culture. Thus, we can be certain that we start from pure populations. We provide additional data to quantify proliferation (Figure S2) that show no differences in EdU incorporation, suggesting active self-renewal of stem cells.

7. Figure 2: The authors do not provide evidence that DENSpm-treated cells indeed reduce global translation rates.

We have carefully quantified mRNA translation using puromycin incorporation and we indeed did not observe changes in allover translation rates upon DENSpm treatment (Figure 2H and I). This has led us to reconsider the conclusions of the paper. This key data piece suggests that, while polyamines can control translation as seen in the DMFO treatment, specific changes in the ratios of the polyamines can have other effects. We thus further focus the investigation on N1-acetylspermidine.

8. The data shown in Figure S2C do not cohere. CD34+/ITGA+ seem more proliferative than CD34-/ITGA+ cells, yet administration of DENSpm does not increase the number of EdU+ cells when all live cells are considered. However, the authors argument is that DENSpm enriches the number of CD34+/ITGA+. A similar argument applies for Figure S2D. It also worrying that the overall survival rate of CD34+/ITGA6+ cells is generally higher indicating that survival might be a driver for the results. For instance, cell stress reduces global translation and CD34+/ITGA6+ cells may simply cope better with stress.

We agree with the reviewer that an increase in the number of ¤6+/CD34+ cells would be expected to reflect on higher proliferation of all live cells upon DENSpm. Presumably, the long incubation period (24h) prohibited a detection of these differences in our first data set. We have adjusted the incubation time for EdU to 2h, as done for N1-AcSpd, and have re-assessed proliferation. Our new data in Figure S2 show that allover proliferation does not differ greatly between ¤6+/CD34+ and ¤6+/CD34- cells. Thus, a shift towards ¤6+/CD34+ cells in the DENSpm treatment would not be expected to change the mixed culture proliferation rates.

© 2021. Published by The Company of Biologists under the terms of the Creative Commons Attribution License (https://creativecommons.org/licenses/by/4.0/).

Similarly careful re-analysis of apoptosis reveals no difference between ¤6+/CD34+ and ¤6+/CD34- cells.

9. Page 9: The authors state: "Taken together, these data suggest that reduced translation is not merely a consequence of stemness. Instead, decreasing translation actively promotes the stem cell state." While this has been shown before in several adult tissues in vivo including skin, it is an overstatement regarding the authors own data because no evidence is provided that the treatment indeed resulted in either global or more specific mRNA translation.

We do not include the G418 data in the updated version of the manuscript and updated the passage accordingly. Also, we have provided additional experimental evidence regarding protein biosynthesis rates. Further, given our new and unexpected observations with DENSpm treatment, we found that this conclusion was not supported any more by our data. This was an important realization as it means that reducing the natural polyamines affects fate decisions but, importantly, this does not occur through reduced translation but rather through N1- acetylspermidine.

10. In addition to directly acting on the translation apparatus to stimulate protein synthesis, polyamine spermidine has been shown to regulate specific translation due to its essential role for eukaryotic translation factor 5A (eIF5A). Multiple studies have also linked lower levels of polyamines with increased misreading during translation elongation. The authors need to provide evidence for a change in global and/or specific protein translation, for instance by performing polysome profiling or ribosome footprinting.

To address the reviewer's comment, we have performed polysome foot printing from isolated ¤6+/CD34+ and ¤6+/CD34- cells, providing a very valuable data set. As suggested by the reviewer, it provides evidence of changes in specific protein translation: ¤6+/CD34+ cells show an enrichment in GO terms associated with protein quality control while ¤6+/CD34- cells reveal elevated translation of protein biosynthetic genes.

11. It has already been demonstrated that over-expression of spermidine/spermine N1-acetyltransferase 1 (SAT1) in mammalian cells was sufficient to suppress protein synthesis via spermidine and spermine (Mandal et al., 2013). The authors should acknowledge these findings.

We are aware of this work and are now citing it appropriately.

12. Similar to my comments above, the finding that N1-acetylspermidine enhances cell proliferation without reducing translation is potentially interesting but unlikely to be independent of a role in translation. The finding that cell cycle regulators are up-regulated do not point to any underlying mechanism.

We thank the reviewer for this insightful comment that is now fully supported by our new ribosome foot printing data. Figure 5 investigates the role of N1-acetylspermidine by transcriptomics and translatomics. As shown before, RNA sequencing shows a regulation of proliferative genes by N1-acetylspermidine in which the treatment enhanced the differences between ¤6+/CD34+ and ¤6+/CD34- cells. In addition, our new data show that N1- acetylspermidine also changes specific translation: we detected changes in the translational efficiency of mRNAs involved in cell division and growth.

References:
Baser, A., Skabkin, M., Kleber, S., Dang, Y., Gulculer Balta, G.S., Kalamakis, G., Gopferich, M., Ibanez, D.C., Schefzik, R., Lopez, A.S., et al. (2019). Onset of differentiation is post-transcriptionally controlled in adult neural stem cells. Nature 566, 100-104.
Blanco, S., Bandiera, R., Popis, M., Hussain, S., Lombard, P., Aleksic, J., Sajini, A., Tanna, H., Cortes-Garrido, R., Gkatza, N., et al. (2016). Stem cell function and stress response are controlled by protein synthesis. Nature 534, 335-340.
Chacon-Martinez, C.A., Klose, M., Niemann, C., Glauche, I., and Wickstrom, S.A. (2017). Hair follicle stem cell cultures reveal self-organizing plasticity of stem cells and their progeny. The EMBO journal 36, 151-164.
Mandal, S., Mandal, A., Johansson, H.E., Orjalo, A.V., and Park, M.H. (2013). Depletion of cellular

© 2021. Published by The Company of Biologists under the terms of the Creative Commons Attribution License (https://creativecommons.org/licenses/by/4.0/).

polyamines, spermidine and spermine, causes a total arrest in translation and growth in mammalian cells. Proc Natl Acad Sci U S A 110, 2169-2174.
Signer, R.A., Magee, J.A., Salic, A., and Morrison, S.J. (2014). Haematopoietic stem cells require a highly regulated protein synthesis rate. Nature 509, 49-54.

Significance:
Several recent studies demonstrated that tissue stem cells often reduce de novo protein synthesis, and that this repression of translation is required for stem cell functions. Polyamines are known to regulate global and specific mRNA translation through several mechanisms but their function in regulating stem cell fate has not been studied yet.

We want to highlight the reviewer's perspective on the significance of our work. By adding additional data, most importantly in vivo experiments and ribosome foot printing, we reach a better understanding of the role of polyamines and translation in stem cell fate decisions.

Second decision letter

MS ID#: JOCES/2020/252767

MS TITLE: N1-acetylspermidine is a determinant of hair follicle stem cell fate

AUTHORS: Kira Allmeroth, Christine S Kim, Andrea Annibal, Andromachi Pouikli, Janis Koester, Maxime J Derisbourg, Carlos Andres Chacon-Martinez, Christian Latza, Adam Antebi, Peter Tessarz, Sara A Wickstrom, and Martin Sebastian Denzel
ARTICLE TYPE: Research Article

We have now reached a decision on the above manuscript.

To see the reviewers' reports and a copy of this decision letter, please go to: https://submit-jcs.biologists.org and click on the 'Manuscripts with Decisions' queue in the Author Area. (Corresponding author only has access to reviews.)

As you will see, the reviewers gave generally favourable reports and agree that you have been substantially responsive in the revision process. However, theyraised some critical points about data interpretation and presentation and argue in several instances that your conclusions are different from or in disagreement with your data. I believe that you likely address these issues with careful rewriting of the text and potentially some limited new data analysis and amendments to the figures. I hope that you will be able to carry these out, because I would like to be able to accept your paper. In the interests of time, I will attempt to assess the completeness of your response myself and so please provide a full response to reviews and both a "clean" version of the manuscript and one in the supplement that uses a different color font for changes.

We are aware that you may be experiencing disruption to the normal running of your lab that makes experimental revisions challenging. If it would be helpful, we encourage you to contact us to discuss your revision in greater detail. Please send us a point-by-point response indicating where you are able to address concerns raised (either experimentally or by changes to the text) and where you will not be able to do so within the normal timeframe of a revision. We will then provide further guidance. Please also note that we are happy to extend revision timeframes as necessary.

Please ensure that you clearly highlight all changes made in the revised manuscript. Please avoid using 'Tracked changes' in Word files as these are lost in PDF conversion.

I should be grateful if you would also provide a point-by-point response detailing how you have dealt with the points raised by the reviewers in the 'Response to Reviewers' box. Please attend to

© 2021. Published by The Company of Biologists under the terms of the Creative Commons Attribution License (https://creativecommons.org/licenses/by/4.0/).

all of the reviewers' comments. If you do not agree with any of their criticisms or suggestions please explain clearly why this is so.

Reviewer 1

Advance summary and potential significance to field

See first review.

Comments for the author

The authors have provided more data to substantiate their claims, but this is on cost of clarity. The main message is confused.
It seems that their observed effects are not due to changes in global translation rates, but this raises the question of what is the underlying reason or function of N1-acetylspermidine in this context.
Other potential roles in metabolism are not considered although polyamines have been linked to mitochondrial respiration for instance (Puleston et al., 2019). This is somewhat surprising as it does come up in their sequencing data in figure 1 and it is also extensively described in the discussion.

1. The authors claim that "Figure 2. Manipulation of polyamine availability demonstrates that mRNA translation rates and stemness do not correlate in the 3D-3C organoids". The figure does not show that translation rates and stemness do not correlate. In fact, the authors show in length that it does in Figure 1. The conclusion of these data rather seems to be that reduction of global protein synthesis is not sufficient to change the differentiation state. Contrary to the authors claim on page 10 line 216, this is not entirely unexpected because cells reduce their protein synthesis in response to many stimuli such as stress and are not expected enter a more undifferentiated state by doing so.
2. In figure 4, the authors claim that "N1-acetylspermidine affects cell fate by increasing cell cycle progression". This statement does not seem to be supported in the figure. While figure 4F shows that ITGA6+/CD34+ positive cells are not depleted in the long-term culture, this data also means that corresponding ITGA6+/CD34-
population also stays constant. Thus, cell fate was not changed at all and that N1-acetylspermidine has the same effect on both populations.
3. Page 18, lines 432-434: Why is it surprising that translation is enhanced? All of Figure 4 shows that cell cycle is increased and protein synthesis does increase with proliferation.
4. It is appreciated that the authors attempt to confirm their findings in vivo but it is entirely unclear to me how a difference of N1-acetylspermidine in young and old mice in late anagen confirms a role in regulating hair follicle stem cell fate.

Puleston, D.J., Buck, M.D., Klein Geltink, R.I., Kyle, R.L., Caputa, G.,
O'Sullivan, D., Cameron, A.M., Castoldi, A., Musa, Y.,
Kabat, A.M., et al. (2019). Polyamines and eIF5A

Reviewer 2

Advance summary and potential significance to field

The authors have addressed the suggested revisions. They added additional data examining the level of acetylated polyamines in vivo and also provided additional control experiments throughout the paper, included more experimental details in the material and methods and went into more depth in discussing their findings. I believe the paper is now suitable for publication in JCS.

Comments for the author

One suggestion would be to consider if possible to normalize between samples and plot data from young and old mice together (Fig. 5d&e). This way a direct comparison of what happens to these

© 2021. Published by The Company of Biologists under the terms of the Creative Commons Attribution License (https://creativecommons.org/licenses/by/4.0/).

levels in aging could be also achieve, in addition to examining changes in response to stem cell activation and hair growth.

Second revision

Author response to reviewers' comments

Reviewer 1 Advance Summary and Potential Significance to Field:
See first review.

Reviewer 1 Comments for the Author:
The authors have provided more data to substantiate their claims, but this is on cost of clarity. The main message is confused. It seems that their observed effects are not due to changes in global translation rates, but this raises the question of what is the underlying reason or function of N1-acetylspermidine in this context. Other potential roles in metabolism are not considered although polyamines have been linked to mitochondrial respiration for instance (Puleston et al., 2019). This is somewhat surprising as it does come up in their sequencing data in figure 1 and it is also extensively described in the discussion.
Puleston, D.J., Buck, M.D., Klein Geltink, R.I., Kyle, R.L., Caputa, G., O'Sullivan, D., Cameron, A.M., Castoldi, A., Musa, Y., Kabat, A.M., et al. (2019). Polyamines and eIF5A

Our response:
We thank the reviewer for the careful re-evaluation of our manuscript. We would like to point out that our new data did not only substantiate our original claims. In fact, guided by the insightful reviewer comments on our manuscript, we performed new experiments that resulted in a re-interpretation of our data that we carefully delineated in the previous response to the reviewer comments and in our paper. Going beyond our first conclusions, we now identify distinct conditions that separate the functions of mRNA translation and polyamine levels in cell fate decisions. We confirm a link between stem cells and low translation and we also describe a role of polyamines that act without reducing translation. We clearly distinguish these two lines of thought throughout the paper for most clarity.
We further thank the reviewer for pointing out the important connection between polyamine metabolism and mitochondrial function. For clarity of the paper, we decided not to also focus on mitochondrial metabolism as the polyamine effects we later observe are explained by effects on the cell cycle. Figure 1G serves to characterize the epidermal stem cells and changes in mitochondrial metabolism were expected. The figure, however, focuses on mRNA translation, consistent with our research question.
Puleston et al. (2019) found that the expression of mitochondrial genes is particularly dependent on the hypusination of eIF5A, but the effects of polyamines we see are explained through other mechanisms. Nevertheless, we cite the paper (Puleston et al., 2019) in the discussion (page 23 lines 537-540) of the re-revised paper to fully cover the potential roles of polyamines in this context.

1- The authors claim that “Figure 2. Manipulation of polyamine availability demonstrates that mRNA translation rates and stemness do not correlate in the 3D-3C organoids”. The figure does not show that translation rates and stemness do not correlate. In fact, the authors show in length that it does in Figure 1. The conclusion of these data rather seems to be that reduction of global protein synthesis is not sufficient to change the differentiation state. Contrary to the authors claim on page 10 line 216, this is not entirely unexpected because cells reduce their protein synthesis in response to many stimuli, such as stress and are not expected enter a more undifferentiated state by doing so.

Our response:
We thank the reviewer for pointing out this important conceptual point. Whereas Figure 1 clearly shows the correlation, Figure 2 shows that low translation does not enhance stemness when polyamines are depleted. Whether the reduction in protein synthesis is insufficient or if

© 2021. Published by The Company of Biologists under the terms of the Creative Commons Attribution License (https://creativecommons.org/licenses/by/4.0/).

polyamines act independently to enhance stemness is further investigated in Figure 2. Data show that DENSpm treatment was itself sufficient for enhanced stemness, without changes in translation. This argues against a contributing role of reduced translation in stemness. We updated the figure title (Figure 2: Changes in polyamine availability enhance stemness independent from reduced mRNA translation.) and the text (page 13 lines 293-297) accordingly.

Given the results shown in figure 1, we found it surprising that a reduction of mRNA translation was not sufficient to affect cell fate. However, we agree with the reviewer that not every stimulus that reduces translation rates would be expected to affect stemness. We updated the text accordingly (page 10 lines 213-215).

2- In figure 4, the authors claim that “N1-acetylspermidine affects cell fate by increasing cell cycle progression”. This statement does not seem to be supported in the figure. While figure 4F shows that ITGA6+/CD34+ positive cells are not depleted in the long-term culture, this data also means that corresponding ITGA6+/CD34- population also stays constant. Thus, cell fate was not changed at all and that N1-acetylspermidine has the same effect on both populations.

Our response:
We thank the reviewer for highlighting this important point. We now highlight in the text that N1-acetylspermidine does indeed affect both populations (page 17 lines 407-409; page 18 line 417). We mention several times throughout the paper that the cells in the organoid culture automatically form a balance between ITG6A+/CD34- and ITG6A+/CD34+ cells (page 7 lines 134-136; page 10 lines 214-218; page 11 lines 241-243). This was also reported by Chacón- Martínez before. This balance is influenced by proliferation and de-differentiation of ITG6A+/CD34- cells and self-renewal and differentiation of ITG6A+/CD34+ cells. Thus, cell fate changes do not only affect HFSCs, but also the ITG6A+/CD34- cells in the culture. Higher proliferation favors stem cell fate by enhancing stem cell maintenance and elevating de- differentiation, which is shown in Figure 3E and discussed on page 25 in lines 575-584. In sum, this results in an enrichment of HFSCs in the culture. We show in Figure 4C that EdU incorporation is increased in both populations. Since proliferation of ITG6A+/CD34- cells was also elevated, we did not expect a depletion of these cells in the long-term culture.
Summarizing, if N1-acetylspermidine had had no effect on the balance of cells in the 3C culture, we would have observed the steady-state 50:50 ratio between stem cells and progenitors. We argue that the effect of N1-acetylspermidine on both populations results in elevated stemness through effects on the cell cycle.

3- Page 18, lines 432-434: Why is it surprising that translation is enhanced? All of Figure 4 shows that cell cycle is increased and protein synthesis does increase with proliferation.

Our response:
We thank the reviewer for highlighting this correlation between cell cycle progression and translation rates. It was surprising to us that an increase in translation, which has been described previously to poise stem cells to differentiate, did not reduce the number of stem cells. Instead, and surprisingly, we found that high translation rates and stemness are not mutually exclusive. However, we agree with the reviewer that based on the results presented in Figure 4 an increase in translation is not that surprising. We edited the text accordingly (page 18 lines 427-428).

4- It is appreciated that the authors attempt to confirm their findings in vivo but it is entirely unclear to me how a difference of N1-acetylspermidine in young and old mice in late anagen confirms a role in regulating hair follicle stem cell fate.

Our response:
We thank the reviewer for appreciating the value of in vivo data in the paper. Whereas the investigation of polyamine levels in HFSCs directly after activation would be desirable, this was impossible due to technical limitations. Nevertheless, the results presented in Figure 5 indicate that the acetylated polyamines play a role in the anagen growth phase, which is affected by cell fate decisions during the telogen/anagen transition and by proliferation. We updated the text to highlight the correlative character of our findings (page 21 lines 495-496). Importantly, our in vivo data reveal an important and specific regulation of N1-acetylspermidine in hair growth, which is in line with our organoid data. For this reason, they strongly support the conclusions of the

© 2021. Published by The Company of Biologists under the terms of the Creative Commons Attribution License (https://creativecommons.org/licenses/by/4.0/).

manuscript.

Reviewer 2 Advance Summary and Potential Significance to Field:
The authors have addressed the suggested revisions. They added additional data examining the level of acetylated polyamines in vivo and also provided additional control experiments throughout the paper, included more experimental details in the material and methods and went into more depth in discussing their findings. I believe the paper is now suitable for publication in JCS.

Reviewer 2 Comments for the Author:
One suggestion would be to consider if possible, to normalize between samples and plot data from young and old mice together (Fig. 5d&e). This way a direct comparison of what happens to these levels in aging could be also achieve, in addition to examining changes in response to stem cell activation and hair growth.

Our response:
The polyamine levels in old mice shown in Figure 5E are already normalized to the levels in young control epidermis. Unfortunately, we had not properly described the normalization in the figure legend. We updated the figure legend accordingly. We decided to not plot the data together because the direct comparison is also possible between the two graphs. Furthermore, the interpretation of the data is easier this way, since the effect of aging is separated from the effect of depilation.

Third decision letter

MS ID#: JOCES/2020/252767

MS TITLE: N1-acetylspermidine is a determinant of hair follicle stem cell fate

AUTHORS: Kira Allmeroth, Christine S Kim, Andrea Annibal, Andromachi Pouikli, Janis Koester, Maxime J Derisbourg, Carlos Andres Chacon-Martinez, Christian Latza, Adam Antebi, Peter Tessarz, Sara A Wickstrom, and Martin Sebastian Denzel
ARTICLE TYPE: Research Article

Thank you for sending your manuscript to Journal of Cell Science through Review Commons.

I am happy to tell you that your manuscript has been accepted for publication in Journal of Cell Science, pending standard ethics checks.

© 2021. Published by The Company of Biologists under the terms of the Creative Commons Attribution License (https://creativecommons.org/licenses/by/4.0/).
